# Manganese-Catalyzed Hydrogenation of Amides and Polyurethanes: Is Catalyst Inhibition an Additional Barrier to the Efficient Hydrogenation of Amides and Their Derivatives?

**DOI:** 10.1021/acs.organomet.3c00399

**Published:** 2024-01-09

**Authors:** James Luk, Conor L. Oates, José A. Fuentes Garcia, Matthew L. Clarke, Amit Kumar

**Affiliations:** EaStCHEM, School of Chemistry, University of St. Andrews, North Haugh, St. Andrews KY16 9ST, U.K.

## Abstract

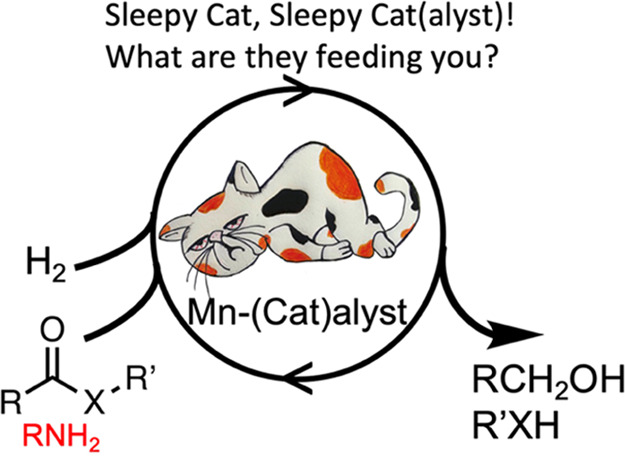

The hydrogenation of amides and other less electrophilic carbonyl derivatives with an N–C=O functionality requires significant improvements in scope and catalytic activity to be a genuinely useful reaction in industry. Here, we report the results of a study that examined whether such reactions are further disadvantaged by nitrogen-containing compounds such as aliphatic amines acting as inhibitors on the catalysts. In this case, an enantiomerically pure manganese catalyst previously established to be efficient in the hydrogenation of ketones, *N*-aryl-imines, and esters was used as a prototype of a manganese catalyst. This was accomplished by doping a model ester hydrogenation with various nitrogen-containing compounds and monitoring progress. Following from this, a protocol for the catalytic hydrogenation of amides and polyurethanes is described, including the catalytic hydrogenation of an axially chiral amide that resulted in low levels of kinetic resolution. The hypothesis of nitrogen-containing compounds acting as an inhibitor in the catalytic hydrogenation process has also been rationalized by using spectroscopy (high-pressure infrared (IR), nuclear magnetic resonance (NMR)) and mass spectrometry studies.

## Introduction

**Scheme 1:**
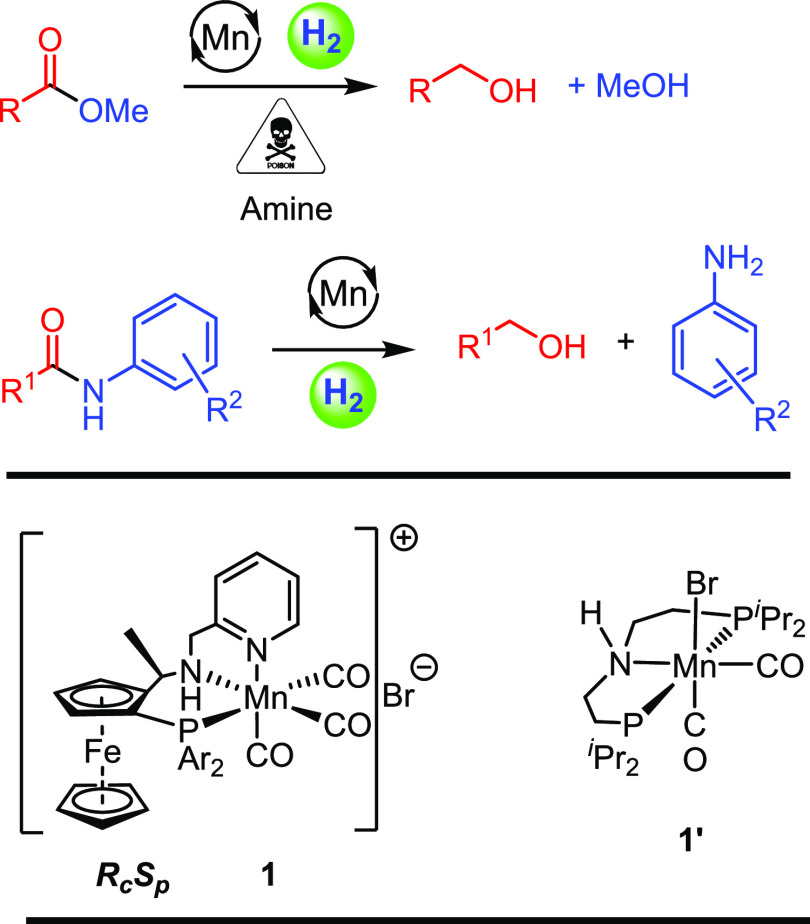
Studies on the Hydrogenation of Esters and Amides Reported Here and the Structure of Manganese Precatalyst **1** (Ar = 3,5-Me_2_C_6_H_3_) and **1′**

Catalytic hydrogenation of carbonyl compounds has important applications in the production of pharmaceutical drugs, fine chemicals, agrochemicals, flavors, fragrances, and even depolymerizations of polymers.^1−5^ The homogeneously catalyzed hydrogenation of esters by well-defined transition-metal complexes has become a synthetically useful alternative to the use of stoichiometric reagents.^4,6^ Various reports using Ru and Mn catalysts have demonstrated both highly chemoselective examples and reactions operating at low catalyst loading (e.g., TON >10,000).^7,8^ The homogeneous hydrogenation of amides, however, has proven to be rather more difficult to develop into a reaction of wide utility. Hydrogenation with C–O bond cleavage only occurs under relatively forcing conditions and uses high catalyst loadings, with the inclusion of additives.^7,9^ Hydrogenolysis of amides with C–N bond cleavage could have several applications, including deprotection of protecting or directing groups,^10^ depolymerization,^11,12^ and possibly for enantioselective hydrogenolysis reactions. However, the reactivity is generally relatively lower compared to the hydrogenation of other carbonyl compounds, such as ketones and esters. More significantly, many types of substrates do not react at all or exhibit poor reactivity.^13^ For example, the literature on the hydrogenolysis of amides using a homogeneous catalyst such as ruthenium,^10,14−19^ iron,^20−25^ and manganese^26^ complexes reports (significantly) lower reactivity of *N*-alkyl amides to form amines and alcohols in comparison to those of *N*-aryl amides. The obvious explanation for this is that the more electron-rich nitrogen of an *N*-alkyl amino group lowers the electrophilicity of the carbonyl group. While it seemed very likely that this is the primary cause of this limitation, there are various examples where free primary amino and hydroxyl groups caused incompatibility in various reactions involving catalytic hydrogenation or dehydrogenation.^27−30^ A research study that investigated the inhibitory or poisoning effect of nitrogen-containing functional groups on the catalytic hydrogenation of carbonyls was, therefore, overdue in our view. Studies on the inhibitory/promotional effects of additives such as thiols,^31^ alcohols,^32,33^ and base^32^ in catalytic hydrogenation reactions have been reported recently. Here, we report not only a study quantifying the inhibition effects of the nitrogen-containing compounds but also a new protocol for amide hydrogenation using the Mn precatalyst, **1** (Scheme 1).

## Results and Discussion

Given that earth-abundant metal complex **1** and various derivatives with differing heterocyclic substituents appear to be very promising for various types of catalytic hydrogenation, this seemed the most timely choice of a catalyst with which to conduct the study.^8,27,34−38^ The hydrogenation of methyl benzoate was chosen as a relatively simple ester hydrogenation that leads to the clean formation of benzyl alcohol and methanol upon hydrogenation.

Previous studies have shown that a range of esters can be reduced at around 50 °C at high pressure (50 bar) showing pseudo-first-order kinetics relative to the ester (e.g., for ethyl-*p*-fluoro-benzoate) using a precatalyst analogous to **1**.^35^ Here, an alternative setup at lower pressure was used with the conditions chosen such that the reaction would occur at a suitable rate, and several samples could be taken during the early stage of the reaction. The hydrogenation reactions were studied using 0.25 mol % manganese complex **1**, 2.5 mol % KO^*t*^Bu (in ^*t*^BuOH), 80 °C, 30 bar H_2_, and 6 mL of MeOH solvent. The experiments were carried out in a pressure vessel, from which small samples could be taken without releasing the hydrogen gas or cooling and then analyzed by ^1^H NMR spectroscopy using an internal standard.

Using this methodology, it was possible to take several samples in the initial 5.5 h of each reaction and evaluate any inhibition and/or change in the induction period. After the fifth sample was taken at 5.5 h, the reactions were continued, and conversion after 22.5 h was measured as a simple measure of catalyst longevity. While this experimental setup is not suited to detailed kinetic analysis, as will be discussed, it proved highly suitable to probe the inhibitory effect.

**Figure 1:**
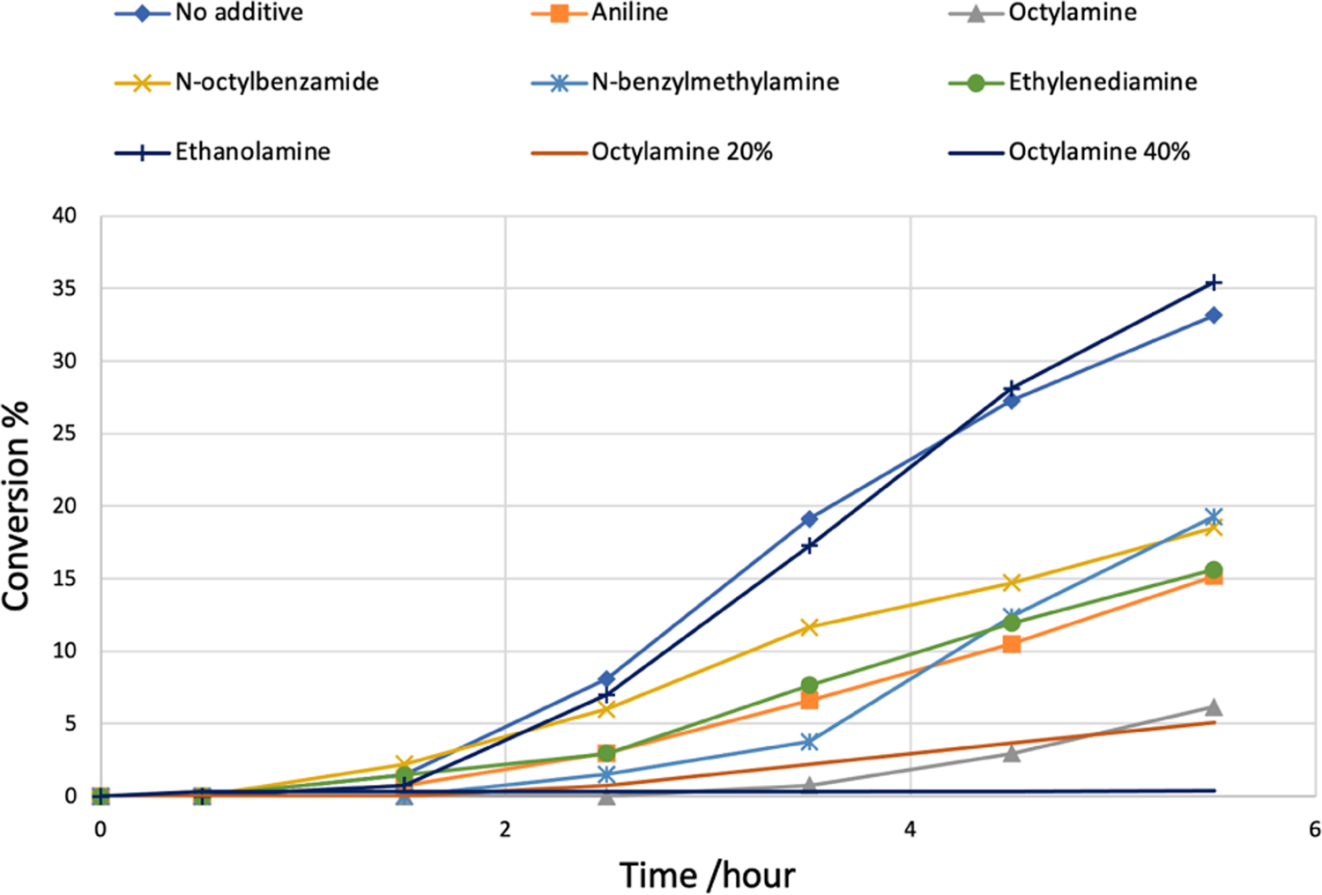
Plot of conversion of methyl benzoate over time upon hydrogenation in the presence of complex **1** and additives as described in Table 1.

All experiments were repeated twice, and the averages of their data are described in Table 1. The TON after 5.5 h are directly proportional to the average TOF for the initial state of the reaction (i.e., between 4 and 35% conversion depending on the inhibitor). Since complex **1** is a precatalyst, there is an induction period for the formation of the active species, which also varies in length from 1 to 2 h with no inhibitor to needing 3–4 h before the rate of conversion curve is at its steepest, depending on the inhibitor studied. Consequently, the discussion of rates any earlier in the reaction is not meaningful. Performing the hydrogenation reaction in the absence of any additive under the aforementioned catalysis conditions shows that the reaction takes 1–2 h to get started. The reaction resulted in 33% conversion of methyl benzoate to benzyl alcohol and methanol in 5.5 h (TON_5.5 h_ = 132). This corresponds to an average TOF over the first 5.5 h of 24 h^–1^. The reaction continues, with 81% conversion being measured after 22 h (Table 1, entry 1). Having set this result as our baseline, we studied the effect of various amine derivatives as potential inhibitors in the hydrogenation of methyl benzoate. Using 10 mol % *N*-octylbenzamide resulted in only a slightly lower rate of conversion of methyl benzoate (19% after 5.5 h, av. TOF = 14 h^–1^, Table 1, entry 2). The *N*-alkyl secondary amine, *N-*benzylmethylamine, and aromatic amine, aniline, show a modest inhibitory effect on the rate of ester hydrogenation (Table 1, entries 3 and 4, orange and light blue lines in Figure 1). Some of this reduced average rate can be ascribed to a longer induction period. Our conclusion is that these *N*-containing compounds exhibit a modest inhibitory effect that might require slightly more forcing conditions. The primary *N-*alkyl amine, octylamine shows a more severe inhibitory effect and a longer induction period as well (Table 1, entry 5, Figure 1). The reaction takes more than 3.5 h before any conversion happens and then is significantly slower than the control reaction (without using any additive). The average TOF over the first 5.5 h using 10 mol % octylamine is around 6 times less than the control reaction. None of these amines appear to irreversibly poison the catalyst at 10 mol % inhibitor loading; for example, using the strongest inhibitor, *n*-octylamine, the reaction kept on producing the product with an increase from around 6 to 40% conversion between when the sampling was terminated (5.5 h) and the reaction stopped (22.5 h, Table 1, entry 5). Two further experiments were carried out with octylamine using 20 and 40 mol % additive loading. In these cases, conversion dropped significantly to 5% and 4%, respectively, in 5.5 h and 12% and 8% in 22.5 h (Table 1, entries 6 and 7). We, therefore, conclude that high concentrations of this primary amine might inhibit the catalyst to such a degree it appears poisoned and inactive. Given the literature is scattered with quite a few examples of potentially chelating products inhibiting hydrogenation catalysts,^27−29,38^ it is interesting here that ethylene diamine just has a modest inhibitory effect and ethanolamine has no inhibitory effect whatsoever.

**Table 1:**
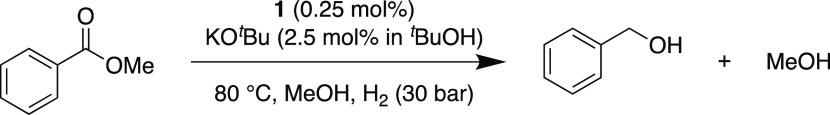
Manganese-Catalyzed Hydrogenation of Methyl Benzoate in the Presence of Various Additives^a^

entry	additive	amount of additive (mol %)	yield (%) (5.5 h)^b^	TON_5.5 h_	yield (%) (22.5 h)^b^
1	no additive		33	132	81
2	*N*-octylbenzamide	10	19	76	55
3	*N*-benzylmethylamine	10	19	76	82
4	aniline	10	15	60	68
5	octylamine	10	6	24	40
6	octylamine	20	5	20	12
7	octylamine	40	4	16	8
8	ethylene diamine	10	16	64	45
9	ethanolamine	10	35	140	83

a Reaction conditions: ester (6 mmol), complex **1** (0.015 mmol), KO^*t*^Bu (0.15 mmol) in ^*t*^BuOH, MeOH (6 mL), 80 °C, 30 bar H_2_.

b Yield is in relation to the formation of benzyl alcohol (found to be the same as the conversion of methyl benzoate) as determined by ^1^H NMR spectroscopy using cyclooctane as an internal standard. No other compound was detected by ^1^H NMR spectroscopy or GCMS.

To investigate if the inhibitory effect of amines is more general to other catalysts or specific to the precatalyst **1**, we conducted the hydrogenation of methyl benzoate using the Mn-MACHO pincer complex **1′** (3 mol %), KO*^t^*Bu (10 mol %), in THF (130 °C, 50 bar H_2_, 24 h) which is a more forcing condition, as is generally used with the complex **1**. Analogous pincer complexes and **1**′ have been studied for the hydrogenation of esters by Beller.^39^ Interestingly, the hydrogenation reaction showed 42% conversion of methyl benzoate in the absence of any additive, whereas the conversion dropped to 28% when 10 mol % aniline was used and 11% when 10 mol % octylamine was used (Table S2). The products detected by ^1^H NMR spectroscopy and GCMS were benzyl alcohol and benzyl benzoate. These observations are suggestive of the generality of our findings to other catalyst systems.

We do not suggest that the strong inhibition of ester hydrogenation in the presence of a primary *N*-alkyl amine additive means that this is the only reason for the low reactivity of the hydrogenation of amides derived from *N*-alkyl amines. However, it is likely that if a more hydridic catalyst is found to accomplish the stoichiometric reduction of such amides, it would still remain a difficult reaction for efficient catalysis unless the catalyst was immune from this inhibition effect. These experiments have utilized only 10–40 mol % inhibitors, but a substrate containing an amine functional group contains by definition 100 mol % amine. Additionally, in an amide hydrogenation, more and more amine is given off, approaching 100 mol % amine as the reaction proceeds to completion. It can be envisaged that significant difficulty would occur in getting the reactions to completion in the case of hydrogenation of amides derived from *N*-alkylamines.

**Figure 2:**
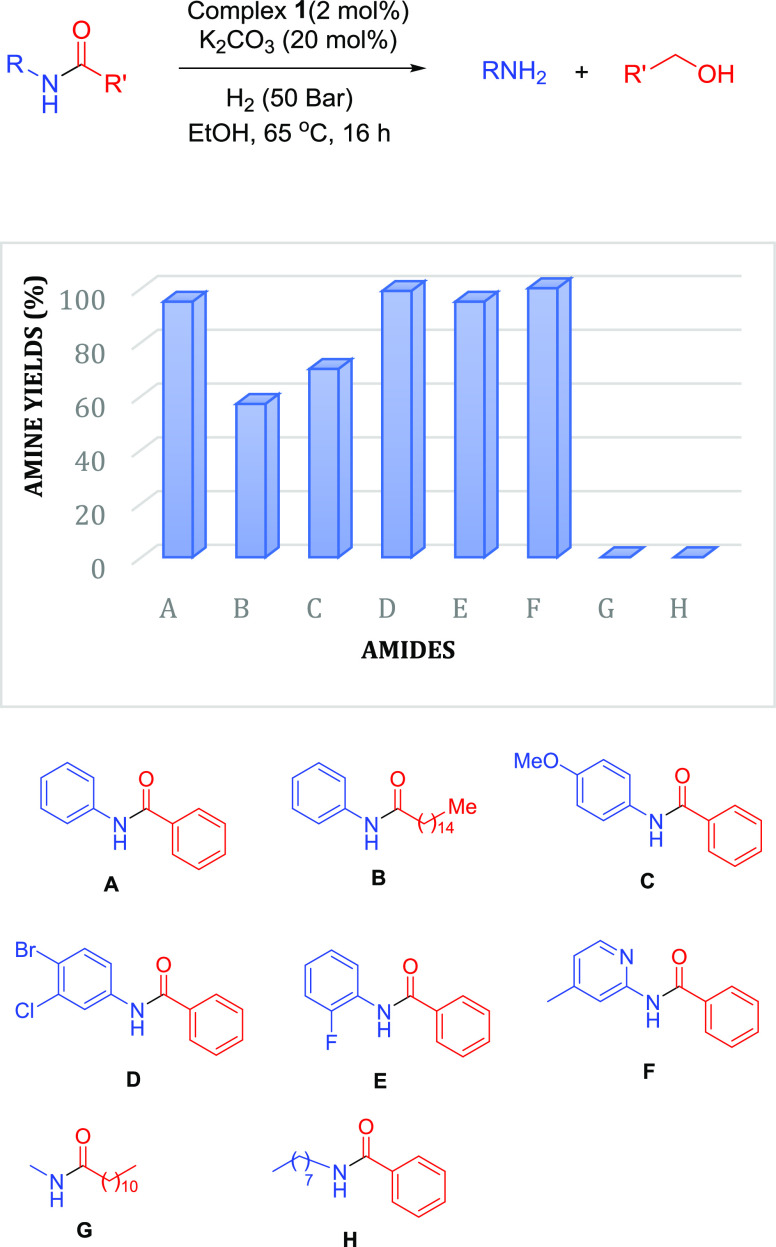
Substrate scope for the hydrogenation of amides. Yield is in relation to the formation of benzyl alcohol as determined by ^1^H NMR spectroscopy using cyclooctane as an internal standard. Products were also detected by GCMS.

Since the above study showed only modest inhibition of both an amide and aniline on the ester hydrogenation catalysis (Table 1, entries 3 and 4), we hypothesized that the hydrogenation of *N*-aryl amides might be viable and not overly impacted by inhibition. Given that catalyst **1** and its derivatives have proven to be unusually reactive in a range of hydrogenations,^8,27,34,35,38,40^ we speculated that **1** might be able to hydrogenate amides to amines and alcohols at relatively modest temperatures, which might have relevance for chiral substrates, and at lower catalyst loadings. There is a literature precedence for manganese-catalyzed amide hydrogenation to alcohols and amines delivering high yields for *N*-aryl amides (and low conversion for *N*-alkyl amides).^26^ This report uses 2–5 mol % of a Mn catalyst at 100 °C for this transformation. Interestingly, the conversion of benzanilide falls to just 18% when 2 mol % of catalyst is used at the lower temperature of 80 °C. To investigate if the high reactivity of catalyst **1** in ketone and ester hydrogenation carried through to amide hydrogenolysis, experiments were carried out using benzanilide at 65 °C. The catalytic conditions were optimized by the variation of manganese precatalyst (**1**), base (KO^*t*^Bu, and K_2_CO_3_), temperature (65 and 50 °C), and concentration (1 and 0.5 M) as described in Table S2. The optimum conditions were found to be 1 mol % complex **1**, 10 mol % K_2_CO_3_, 16 h, 65 °C, and EtOH solvent that led to the quantitative hydrogenation of benzanilide to form benzyl alcohol and aniline. However, under these conditions, hydrogenation of various *N*-aryl amides such as **B**, **C**, and **D** resulted in low conversion (see Table S3). Increasing the loading of complex **1** to 2 mol % and K_2_CO_3_ to 20 mol % while keeping the remaining conditions the same led to the excellent conversion of various *N*-aryl amides (**A**–**F**) to the corresponding aryl amines and alcohols as shown in Figure 2 (more details in Table S3). Amides **B** and **C** are expected to be less electrophilic than amides **A**, **D**, **E**, and **F** and also present somewhat lower conversions. This is consistent with the amide electrophilicity being the primary factor in determining reactivity. Moreover, even less electrophilic *N*-methyldodecanamide (**G**) and N-octylbenzamide (**H**) derived from alkyl amines did not show any conversion under these conditions. This is likely to be mainly due to the lower electrophilicity described earlier. Catalyst inhibition would also come into play if different conditions were employed that did start to convert these *N*-alkyl amides. No side products were observed in these reactions, and the yields of corresponding alcohols and amines were similar to those of conversions (Table S3).

**Scheme 2:**
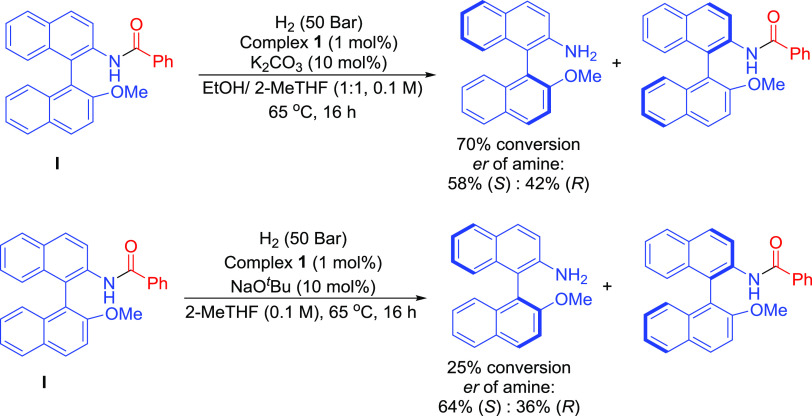
Amide Hydrogenolysis with Kinetic Resolution Using Manganese Precatalyst **1**

Given that complex **1** is enantiomerically pure, we wondered if it would be possible to conduct amide hydrogenolysis with kinetic resolution. This could then become a useful way to obtain axially chiral binaphthyl amine derivatives which are relatively difficult to synthesize in enantiomerically pure form. We are not aware of studies on hydrogenolysis of axially chiral amides (or esters) with kinetic resolution; only one precedent on the enantioselective hydrogenation of amides is available, but with C-centered chirality via dynamic kinetic resolution,^41^ while a conceptually related desymmetrization of Bringmann lactones to produce axially chiral diols has been reported.^42,43^ In order to explore this idea, hydrogenation of axially chiral racemic amide **I** was attempted. While it was pleasing that this hindered substrate could also be hydrogenated with 70% conversion obtained, based on analysis of the amine product, there was no strong indication of significant enantioselectivity, even when the reaction was stopped or operated such that it only reached conversions well below 50% (Scheme 2 and Table S5). The use of a related method to prepare enantiomerically pure binaphthyl amine derivatives using hydrogenolysis with kinetic resolution would be a worthwhile goal in the future but is likely to need different catalysts and/or substrates.

Manganese catalysts have also been studied for the hydrogenative depolymerization of plastics such as polycarbonates^44,45^ and polyurethanes.^46−48^ Motivated by the high activity toward the hydrogenation of aromatic amides using complex **1**, we attempted to hydrogenatively depolymerize an aromatic polyamide, Kevlar (commercial fabric). However, we did not observe any conversion at 130 °C, 50 bar H_2_, using 2 mol % complex **1** and 20 mol % K_2_CO_3_ due to the insolubility of the polymers in the solvents of choice (EtOH, THF, see Table S4). To further compare the reactivity of *N*-aryl and *N-*alkyl amine-derived substrates, we made two polyurethanes (**PU1** and **PU2**) containing aliphatic and aromatic diamines and studied their hydrogenation using complex **1** (2 mol %), K_2_CO_3_ (20 mol %), 130 °C, 50 bar H_2_ for 16 h (Table 2). Performing the hydrogenation of polyurethanes in EtOH solvent led to the formation of *N*-ethylated amines as one of the byproducts presumably from the reaction of the EtOH solvent with intermediates/products obtained from the hydrogenation. We, therefore opted for THF as a more inert solvent. The products obtained from the hydrogenation of polyurethanes in THF under the abovementioned catalytic conditions were diol, diamine, and formamide (mono- and diformamide) as detected by GCMS and ^1^H NMR spectroscopy. In the case of **PU1**, 1,4-butanediol was obtained in a 20% yield. A combined yield of 20% was also obtained for 4,4′-methylenedianiline, *N*-(4-(4-(methylamino)benzyl)phenyl)formamide, and *N*,*N*′-(methylenebis(4,1-phenylene))diformamide. However, interestingly, no conversion was obtained, and the starting material was completely recovered in the case of **PU2** which is made of 1,4-butanediol and hexamethylenediisocyanate.

**Table 2:**
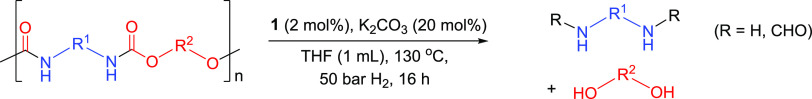

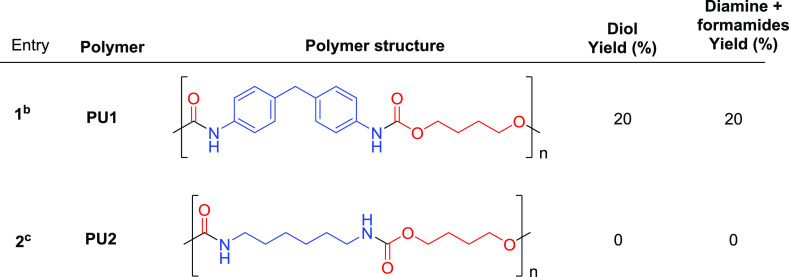
Hydrogenative Depolymerization of Polyurethanes^a^

a Reaction conditions: polyurethane (0.25 mmol), complex **1** (2 mol %), K_2_CO_3_ (20 mol %), THF (1 mL), 130 °C, 50 bar H_2_, 16 h. Products were identified using GCMS, and yields were estimated by ^1^H NMR spectroscopy using 1,1-diphenylethylene as an internal standard.

b ∼75% of the polymer/oligomer was recovered by weight.

c 100% of the polymer/oligomer was recovered by weight.

To obtain explicit evidence that the amine inhibition would be relevant in amide or carbamate hydrogenation, experiments were carried out to observe the impact on the reaction outcome in the presence of an amine additive. We performed the hydrogenation of benzanilide under the reaction conditions described in Figure 2 (2 mol % complex **1** and 20 mol % K_2_CO_3_, 65 °C, 50 bar, 16 h) in the presence of 40 mol % aniline and 40 mol % octylamine. The reaction conducted in the presence of octylamine showed higher inhibition resulting in 74% yield of benzyl alcohol, whereas that in the presence of aniline led to 83% yield of benzyl alcohol (Table S4). It is noteworthy that without adding any additional amine, the hydrogenation reaction under the same conditions leads to >99% yield of benzyl alcohol (using either 1 or 2 mol % of catalyst) suggestive of an inhibitory effect of amines on the hydrogenation of amides.

**Figure 3:**
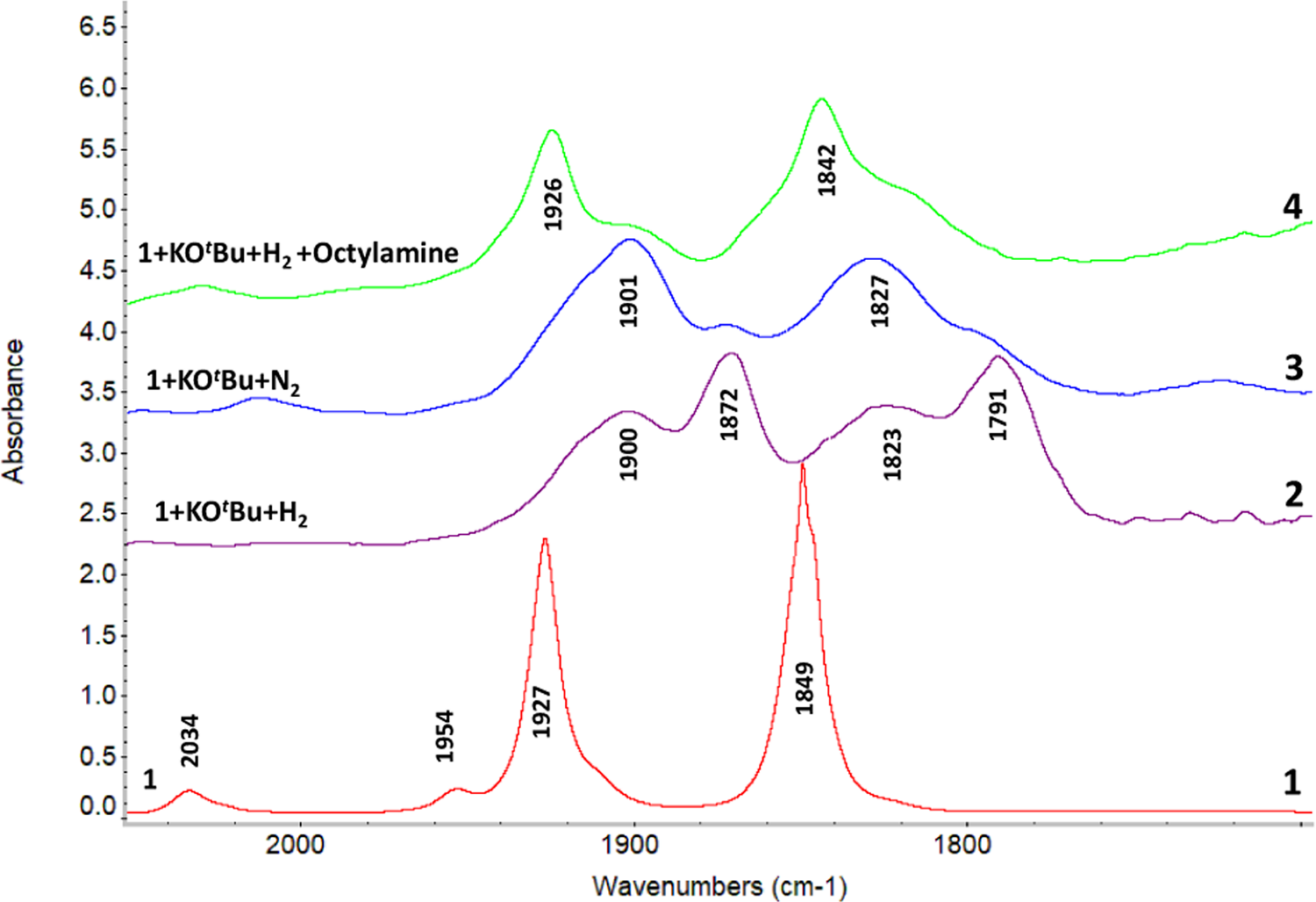
HPIR spectra of complex **1** (spectrum 1), complex **1** after the addition of KO*^t^*Bu in the presence of hydrogen (1 h) (spectrum 2), complex **1** after the addition of KO*^t^*Bu in the presence of nitrogen (1 h) (spectrum 3), followed by the addition of octylamine in the presence of H_2_ (4 h) (spectrum 4). *T* = 38 °C.

**Scheme 3:**
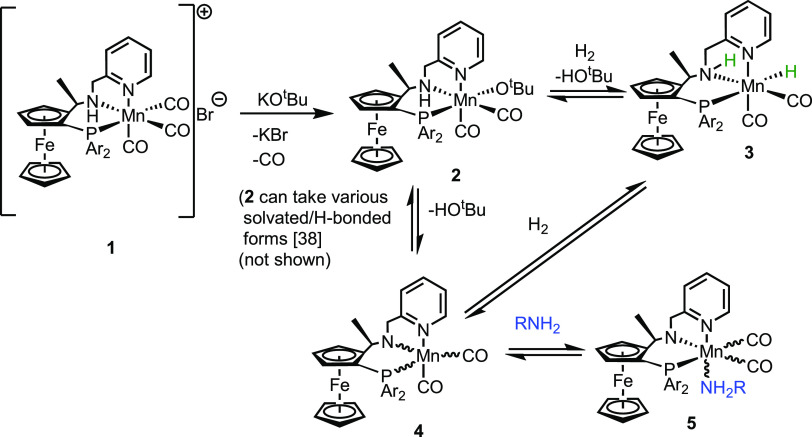
Proposed Pathway for the Formation of Complex **5** Note: metal stereochemistries for **2**, **4**, and **5** are unknown, and the reactive isomer of complex **3** is drawn. For a discussion of this and the base-assisted mechanism of H_2_ activation, see ref (38). R = *n*-octyl.

To rationalize the hypothesis of catalyst inhibition by amines, we attempted to identify organometallic intermediates that might be formed in the hydrogenation process in the presence of an octylamine as an additive. Previous studies have suggested that complex **1** reacts in the presence of a base to form a manganese amido complex **4** that can immediately react with an alcohol to form a more stable alkoxide complex such as **2**. Complexes **2** or **4** can activate H_2_ via metal–ligand cooperation^49^ to form a manganese hydride complex **3** (Scheme 3).^38^ However, in previous work, preliminary attempts at detecting the manganese(I) hydride species using NMR spectroscopy failed, with free ligand being the dominant product from reactions of complex **1** with base and hydrogen (1 bar).^50^ We speculate that a high pressure of hydrogen gas might be needed to observe a manganese(I) hydride complex. In order to study the reaction of **1** with base and hydrogen and octylamine, an experiment was conducted using *in situ* high-pressure infrared (HPIR) spectroscopy. While this technique is less informative for structure elucidation, it can be conducted under significant pressure of hydrogen and otherwise kept free of air and moisture that might lead to decomposition of the active species. The aim was to study the effect of octylamine on the manganese hydride complex that can be distinguished among three likely outcomes from the treatment with octylamine: (i) the spectra associated with the hydride or other resting state is unchanged or barely changed, (ii) the presence of octylamine leads to complete decomposition leading to several new complexes, or (iii) the formation of Mn-octylamine coordination complexes from the Mn-hydride. Studies conducted in methyl THF showed a poor signal/noise ratio in the carbonyl region because of which we moved to study other solvents. No change in the IR spectrum was obtained when complex **1** was heated with KO*^t^*Bu in dodecane (80 °C) or with K_2_CO_3_ in DCM (38 °C) in the presence of hydrogen likely due to the lack of solubility of bases under these conditions. Interestingly, heating a solution of complex **1** and KO*^t^*Bu (10 equiv relative to **1**, in *^t^*BuOH) in DCM at 38 °C under 20 bar of hydrogen resulted in the appearance of new signals in the carbonyl region in comparison to complex **1**. The main bands observed (Figure 3) are two sets of somewhat broadened overlapping bands: one pair at 1900 and 1823 cm^–1^ and the other pair at 1872 and 1791 cm^–1^. An experiment carried out with 10 bar technical grade D_2_ gas in place of 20 bar H_2_ gas initially showed very similar spectra (see Figures S20–S22). After time, the pair of bands at 1900 and 1823 were predominant with similar relative intensity. This strongly suggests that the pairs of bands are from CO and not from a Mn–H vibration. A control experiment carried out under a nitrogen atmosphere in the presence of KO*^t^*Bu (in *^t^*BuOH) leads to mainly the pair of bands at 1901 and 1827 cm^–1^. We suggest that this species is either some form of Mn-alkoxide such as **2** or an Mn-amido complex (i.e., with pincer ligand deprotonated, **4**). The remaining pair of bands at 1872 and 1791 cm^–1^, observed as the major species from the spectrum obtained under a hydrogen atmosphere, cannot be assigned with 100% confidence as either an Mn-hydride, **3** (which could exist as either mer or fac isomers) or one of either **2** or **4**. Irrespective of the exact structures of these two resting states, the important question is what effect does octylamine have on these complexes, which could be resting states in the catalysis.

After treatment with 20 equiv of octylamine (to complex **1** + KO*^t^*Bu in the presence of H_2_), the two complexes seem to undergo a reaction to give a manganese–carbonyl compound(s) that displays just two main bands in the Mn–CO region with quite high intensity (1926 and 1842 cm^–1^), alongside unreacted catalyst resting states at 1901/1827 cm^–1^. When a similar experiment was conducted but using the much weaker inhibitor aniline, a similar type of spectra was observed with two Mn-carbonyl bands with high intensity at 1926 and 1844 cm^–1^.

While it is not possible to fully elucidate the structure of this complex, a number of useful conclusions can be made. The HPIR experiments suggest that octylamine does not cause complete decomposition of the manganese complexes present in the reaction mixture. Such reactivity would lead to either a complex metal carbonyl region or a nearly empty one, and it would not be expected for aniline to show the same behavior since it is only a weak inhibitor. The formation of a single complex from the two resting states suggests that the amine is bound to the Mn. The IR bands observed are therefore tentatively assigned to a complex of formula, [Mn(L–H)(CO)_2_(octylamine)] (complex **5**), with (L–H) representing the deprotonated amido, *P*,*N*,*N* ligand, or a complex in which the octylamine is deprotonated instead, [Mn(L)(CO)_2_(octylamido)] or a cationic species, [Mn(L)(CO)_2_(octylamine)]^+^ with a counteranion and L representing the neutral *P*,*N*,*N* ligand (the latter two constitutional isomers of **5** are not drawn).

Aniline forms an analogous complex, and it would be expected that a primary alkyl amine would be more strongly coordinating and therefore less easily displaced by dihydrogen to regenerate the Mn-hydride and continue catalysis. This would fit with the observations that the octylamine and aniline inhibit catalysis extensively for the former, but the cycle still turns over even after 1 day of reaction time. We also conducted the hydrogenation of methyl benzoate in DCM/MeOH (1:1) solvent mixture using 1 mol % **1**, 10 mol % KO*^t^*Bu, 80 °C, and 50 bar H_2_. A quantitative yield of benzyl alcohol was obtained at the end of 24 h, suggesting that DCM does not poison the hydrogenation reaction.

To further probe the formation of complex **5**, we performed a reaction of complex **1** (0.02 mol, toluene-*d*_8_) with KO*^t^*Bu (0.06 mmol) in a Young’s NMR tube and studied the reaction progress at room temperature by NMR spectroscopy. A ^31^P{^1^H} NMR spectrum taken after 10 min at room temperature showed the complete consumption of the signal corresponding to complex **1** (δ 87.8 ppm). The main new signal was observed at δ 69.2 ppm, likely to be either complex **2** or **4**. The presence of some free ligand (δ −26.2 ppm) and unidentified metal complexes was also observed (Figure S2). The addition of 0.025 mmol of octylamine to the reaction mixture led to the complete disappearance of the signal at δ 69.2 ppm and the appearance of a new signal at δ 86.6 ppm in 3 h at room temperature. It was not possible to isolate a pure compound from the reaction mixture. Interestingly, conducting an ESI-MS analysis of the resulting reaction mixture showed a signal at 882.25 Da, which was identified to be complex **5**(+Na). These observations are suggestive of the possibility of the coordination of an amine to the manganese center in the presence of hydrogen that could block the active site inhibiting the catalytic hydrogenation.

## Conclusions

In conclusion, from a variety of nitrogen functional groups studied as potential inhibitors of Mn-catalyzed hydrogenation reactions, it is only the primary alkyl amine that shows a severe inhibitory effect, approximately reducing the rate of product formation by around 4-fold. Other amine derivatives might have more modest impacts that could come into play in preventing the reaction from reaching full conversion. Based on an HPIR study, we suggest that alkyl amine might coordinate with the catalyst’s resting state strongly inhibiting the formation of a manganese hydride complex eventually leading to poor turnover (Scheme 3). Although we chose the Mn complex **1** as a model precatalyst to examine this phenomenon, the low activity for the hydrogenation of N-alkyl amides using other catalysts in the literature suggests that the amine inhibition could be a more general phenomenon to other catalysts. In agreement with this, brief experiments using the Mn-MACHO pincer complex (**1**′) showed the same inhibition by octylamine. It is therefore likely that even if more hydridic catalysts were available that enable hydrogenation of less electrophilic N-alkyl amides and polyurethanes, high temperatures and relatively high catalyst loadings or removal of the primary alkyl amine from the reacting solution would likely be needed for the efficient hydrogenation.

We have also demonstrated that precatalyst **1** operates under milder conditions than those of the previously reported manganese catalysts for amide hydrogenation. This suggests that complex **1** presents lower energy barriers for amide reduction than the literature systems. Most of the substrates investigated could be reduced at moderate temperatures, provided that the amide was an *N*-aryl amide. While the attempt at kinetic resolution during amide hydrogenolysis was not enantioselective, it is hoped either changing substrate or catalysts could make this an interesting approach to molecules that generally rely on classical resolutions to be made in enantiomerically pure form.

## Supporting Information Available

Catalytic studies and characterization details (PDF)

## Notes

The research data supporting this publication can be accessed at https://doi.org/10.17630/38acf31f-5dc4-48ba-9182-aa5f56c31a53.
