# Supplementary material for: Manganese-Catalyzed Hydrogenation of Amides and Polyurethanes: Is Catalyst Inhibition an Additional Barrier to the Efficient Hydrogenation of Amides and Their Derivatives?
Source: Organometallics. 2024 Jan 9;43(2):85–93. doi: 10.1021/acs.organomet.3c00399 (PMC10806803; doi:10.1021/acs.organomet.3c00399)
Supplement: Supplementary file 1 — om3c00399_si_001.pdf [file om3c00399_si_001.pdf]

Manganese catalysed hydrogenation of amides and polyurethanes: Is catalyst inhibition an additional barrier to efficient hydrogenation of amides and their derivatives?

James Luk, Conor L. Oates, José Fuentes Garcia, Matthew L. Clarke*, and Amit Kumar*

School of Chemistry, University of St. Andrews, North Haugh, St. Andrews, KY169ST, UK.

* Matthew L. Clarke (mc28@st-andrews.ac.uk)
* Amit Kumar (ak336@st-andrews.ac.uk)

Table of Contents

**1. General information**	**S3**
**2. Monitoring the reaction of complex 1 with base and amine**	**S3**
**2.1. General procedure for mechanistic investigations**	**S3**
**2.2. Analytical data for mechanistic catalytic investigations**	**S4**
**3. Hydrogenation of methyl benzoate in the presence of an inhibitor**	**S6**
**3.1. General procedure for the hydrogenation of methyl benzoate in the presence of an inhibitor**	**S6**
**3.2. Methyl benzoate hydrogenation**	**S7**
**3.3. Characteristic data for the hydrogenation of methyl benzoate**	**S8**
**3.4. Kinetic plots for the hydrogenation of esters in the presence of various inhibitors**	**S9**
**4. High pressure infrared spectroscopy**	**S15**
**4.1. General procedure for HPIR spectroscopy**	**S15**
**4.2. HPIR spectroscopic study of Mn complex 1 in the presence of base and D_2**	**S17**
**5. Control experiment of hydrogenation of methyl benzoate in MeOH and DCM**	**S19**
**5.1. General procedure for hydrogenation of methyl benzoate in presence of DCM**	**S19**
**5.2. NMR and GCMS data for hydrogenation of methyl benzoate in presence of DCM**	**S19**
**6. Hydrogenation of amides**	**S20**
**6.1. General Procedures used in the hydrogenation of amides**	**S20**
**6.2. Optimisation studies for the catalytic hydrogenation of benzanilide**	**S21**
**6.3. Hydrogenation of amides in the presence of manganese complex 1**	**S22**
**6.4. Characterisation data from the hydrogenation of amides in the presence of manganese complex 1**	**S24**
**7. The kinetic resolution of N-aryl amides**	**S39**

**7.1. General procedure from the kinetic resolution of N-aryl amides**	**S39**
**7.2. NMR spectra for the kinetic resolution of N-aryl amides**	**S42**
**7.3. Hydrogenolysis of N-aryl amide with kinetic resolution using manganese complex 1**	**S46**
**8. Hydrogenation of polyurethanes**	**S47**
**8.1. General procedures for hydrogenation of polyurethanes**	**S47**
**8.2. Results from the hydrogenation of polyurethanes**	**S47**
**8.3. NMR and IR data for model polyurethanes**	**S48**
**8.4. NMR and GCMS data for model polyurethanes hydrogenation**	**S53**
**9. References**	**S56**

1. General Information

All experiments were carried out under an inert atmosphere of purified nitrogen using standard Schlenk techniques unless specified. The catalytic experiments were carried out using high-pressure vessels rated to contain pressures well in excess of those used in the paper. The catalytic reactions were carried out in a vessel equipped with a bursting disc set well below the pressure vessel threshold (100 bars). Reproduction of these experiments or similar ones should only be carried out using appropriate equipment. A blast shield should be used when manipulations are carried out and appropriate risk assessment. Hydrogen is a flammable gas, so should also be kept away from sources of ignition. Complexes **1**[1] and **2**[2] were prepared as described by the method reported in the literature. All esters, amines, amides (except N-octylbenzamide), K_2CO_3, and KO^{t}Bu were purchased from Sigma- Aldrich, Alfa Aesar, Strem, or TCI and used as received. THF and toluene were dried using a solvent purification system and degassed by Freeze-Pump-Thaw under nitrogen. Anhydrous Me-THF and EtOH were purchased from Sigma-Aldrich and used as received. Deuterated solvents – $CDCl_3$, were purchased from Sigma-Aldrich and used as received.

NMR spectra were recorded on a Bruker AVIII-HD 500 MHz and 400 MHz NMR spectrometer at 298 K unless otherwise specified. All chemical shifts (δ) are quoted in ppm and coupling constants (*J*) in Hz.

GC-MS samples were prepared in HPLC grade DCM and data were collected in an Agilent 8860 GC system coupled to an Agilent 5977B EI instrument.

2. Monitoring the reaction of complex 1 with base and amine

2.1. General procedure for mechanistic investigations

Complex **1** (15.2 mg, 0.02 mmol) and deuterated toluene (0.5 mL) were added to a Young's NMR tube in a glovebox and a $^{31}P\{^1H\}$ NMR of the mixture was taken after 10 min at room temperature. KO^{t}Bu (6.7 mg, 0.06 mmol) was then added to the NMR tube and another $^{31}P\{^1H\}$ NMR spectrum was taken after 5 h (room temperature). Octylamine (3.3 µL, 0.025 mmol) was then added in the same way and a $^{31}P\{^1H\}$ NMR spectrum was taken after 3 h (room temperature). Recrystallisation was then attempted by layering the mixure with hexane and leaving the mixture in a glovebox freezer (-30 °C) for a week but no crystals were obtained. Volatiles were then removed *in vacuo*

and the remaining mixture was dissolved in degassed MeCN and analysed by the ESI mass spectrometry.

2.2. Analytical data for mechanistic catalytic investigations

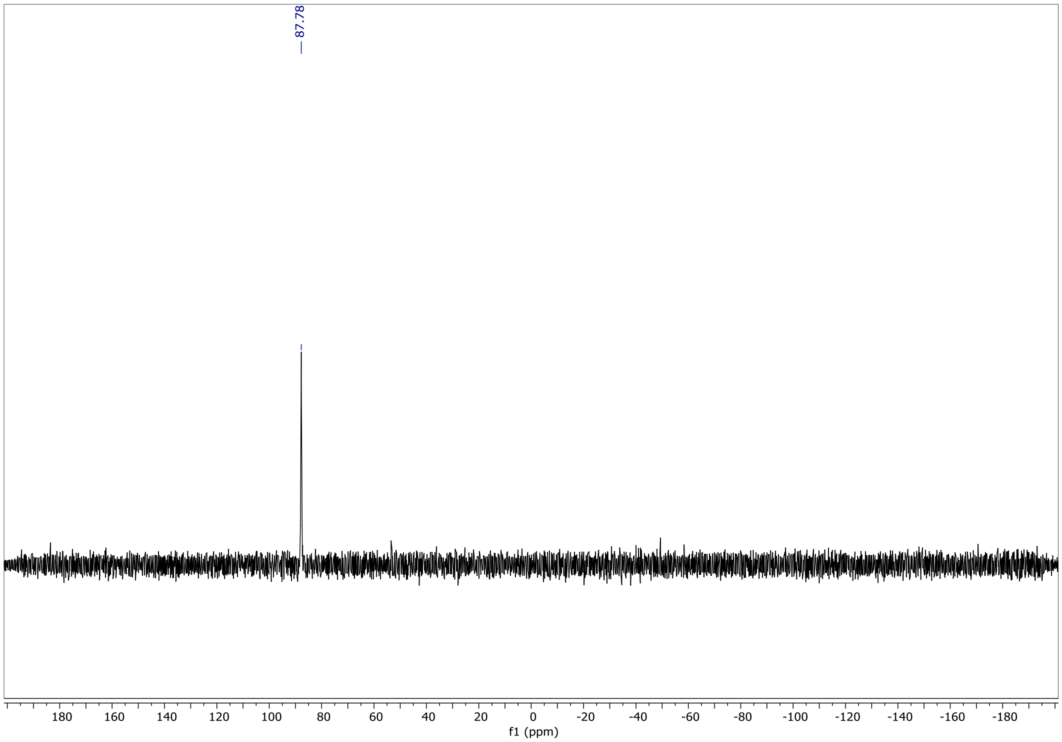

Figure S1: $^{31}P\{^1H\}$ NMR spectrum of complex **1**.

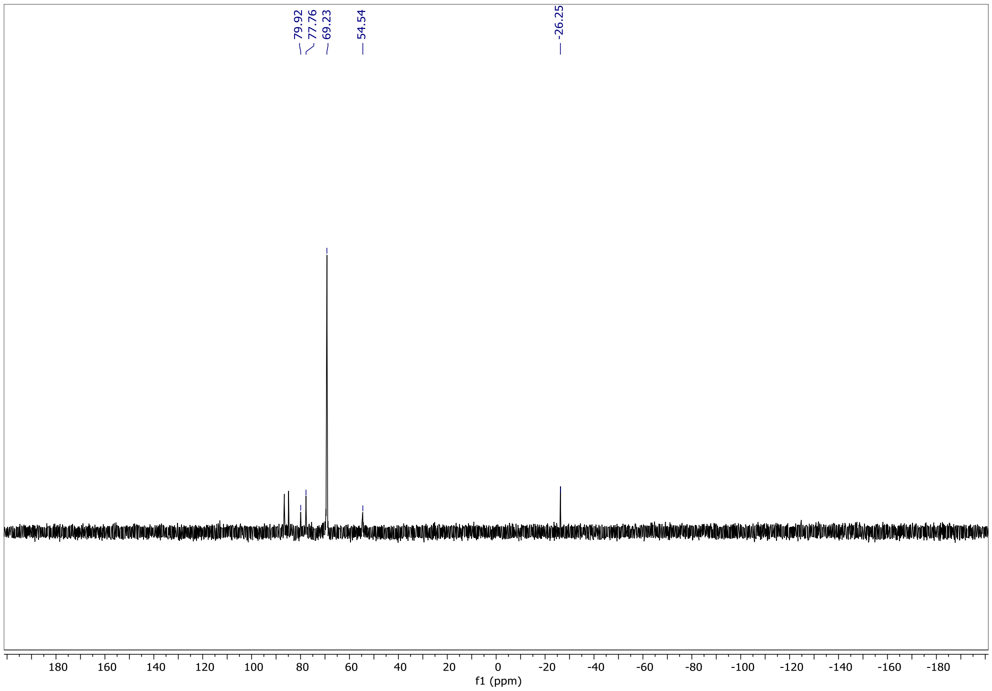

Figure S2: $^{31}P\{^1H\}$ NMR spectrum from the reaction of complex **1** with KO^tBu.

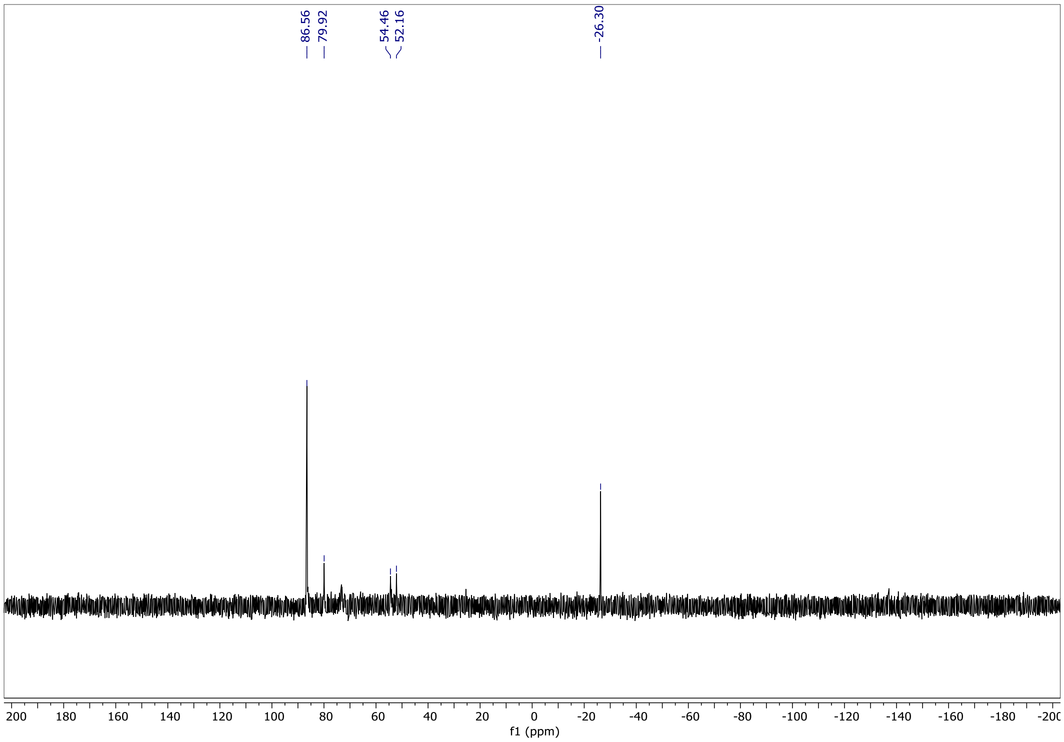

Figure S3: $^{31}P\{^{1}H\}$ NMR spectrum from the reaction of complex **1** with KO^{t}Bu and octylamine.

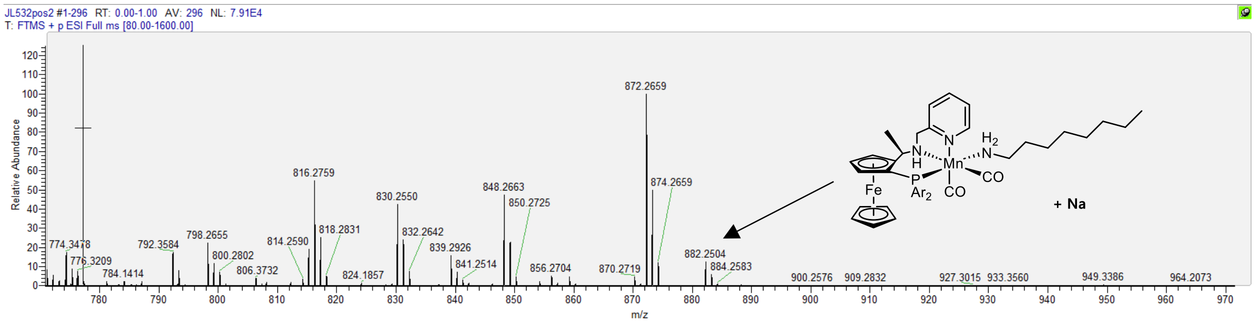

Figure S4: ESI MS spectrum from the reaction of complex **1**, KO^{t}Bu, and octylamine.

3. Hydrogenation of methyl benzoate in the presence of an inhibitor

3.1. General procedure for the hydrogenation of methyl benzoate in the presence of an inhibitor

General Procedure A: Hydrogenation of methyl benzoate in sampling vessel

Complex **1** (11.4 mg, 0.015 mmol) was added to the sampling vessel alongside the appropriate additive amine or amide in its desired quantity as given in Table S1. The vessel was evacuated and purged with nitrogen three times before adding methyl benzoate (756 µL, 6 mmol), cyclooctane (202 µL, 1.5 mmol), and methanol (6 mL). Finally, potassium *t*-butoxide (0.15 mL, 1 mol dm^{-3} (in *t*-butanol), 0.15 mmol) was added to the vessel. The vessel was then charged with 30 bar of H_2 pressure and stirred and heated at 80 °C taking measurements at t = 0 h, 0.5 h, 1.5 h, 2.5 h, 3.5 h, 4.5 h, 5.5 h, and 22.5 h. The yield of the product (benzyl alcohol) was then calculated using the integral of the CH_2 signal of Ph-CH_2-OH at 4.66 ppm relative to the integral of the substrate -OCH_3 signal at 3.89 ppm, using cyclooctane as an internal standard. All experiments were repeated thrice, and their average values have been reported in the paper.

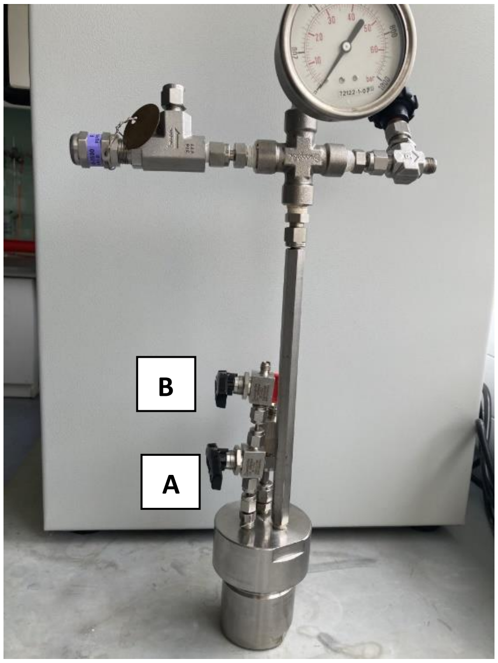

A picture of the sampling vessel is shown here. Under the reaction condition, knob A is opened which fills the tube between both knobs with an aliquot of the reaction mixture. Knob A is then shut followed by opening knob B through which the sample is withdrawn using a syringe.

3.2. Methyl Benzoate Hydrogenation

Table S1: Hydrogenation of methyl benzoate in the presence of various inhibitors

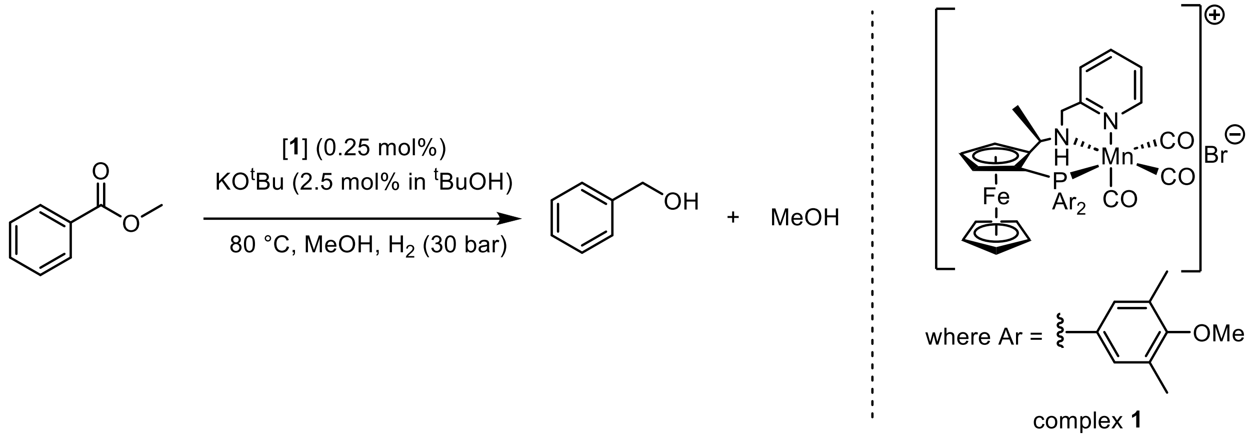

Entry	Additive	Amount of additive	Yield (22.5 h)*	Yield (5.5 h)*	$TON_{5.5\,h}$
**1.**	No additive		81	33	132
**2.**	N-octylbenzamide	10 mol%	55	19	76
**3.**	N-benzylmethylamine	10 mol%	82	19	76
**4.**	Aniline	10 mol%	68	15	60
**5.**	Octylamine	10 mol%	40	6	24
**6.**	Octylamine	20 mol%	12	5	20
**7.**	Octylamine	40 mol%	8	4	16
**8.**	Ethylenediamine	10 mol%	45	16	64
**9.**	Ethanolamine	10 mol%	83	35	140

Table S2: Hydrogenation of methyl benzoate in presence of complex 2[a]

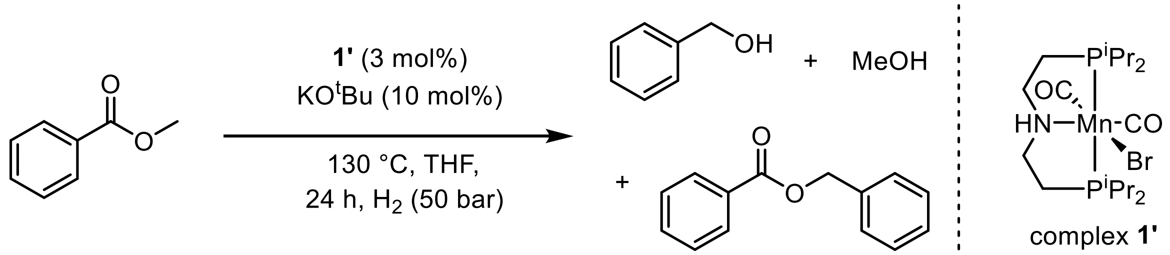

Entry	Additive	Amount of additive	Conversion (%)	Yield benzyl alcohol (%)	Yield benzyl benzoate (%)
**1.**	No additive		42	10	17
**2.**	Aniline	10 mol%	28	4	12
**3.**	Octylamine	10 mol%	11	5	3

[a]standard conditions: 1 mmol methyl benzoate, 3 mol% **1’**, 130 °C, 24 h, 50 bar H_2, 1 mL THF. The products were detected by GC-MS and the conversions/yields estimated by 1H NMR spectroscopy using cyclooctane as an internal standard.

3.3. Characteristic data for the hydrogenation of methyl benzoate

3.3.1. Representative ^{1}H NMR and GCMS spectra for the hydrogenation of methyl benzoate

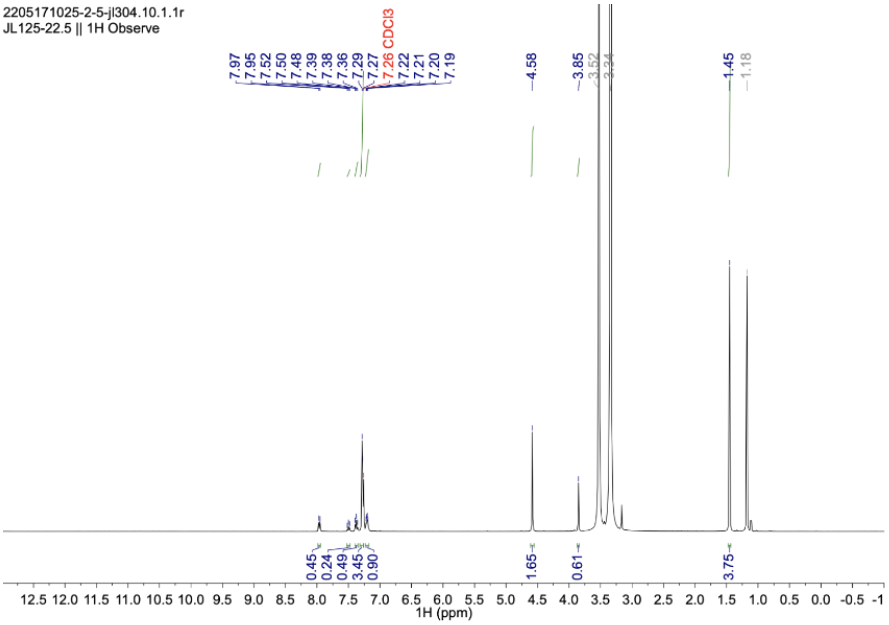

Figure S5: ^{1}H NMR spectrum ($CDCl_3$, 298K) of hydrogenation of methyl benzoate in the presence of no additive.

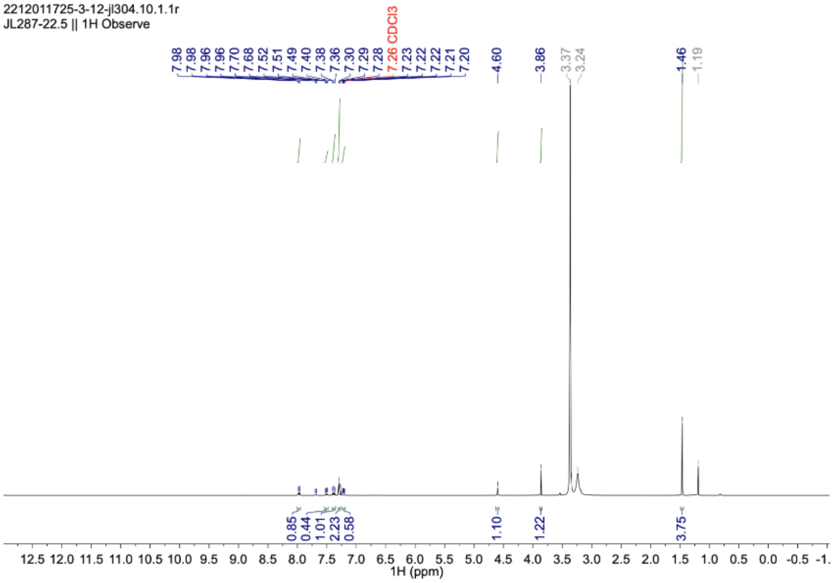

Figure S6: ^{1}H NMR spectrum ($CDCl_3$, 298K) of hydrogenation of methyl benzoate in the presence of N-octylbenzamide.

3.3.2. Representative GCMS data for the hydrogenation of methyl benzoate in the presence of an inhibitor (N-octylbenzamide)

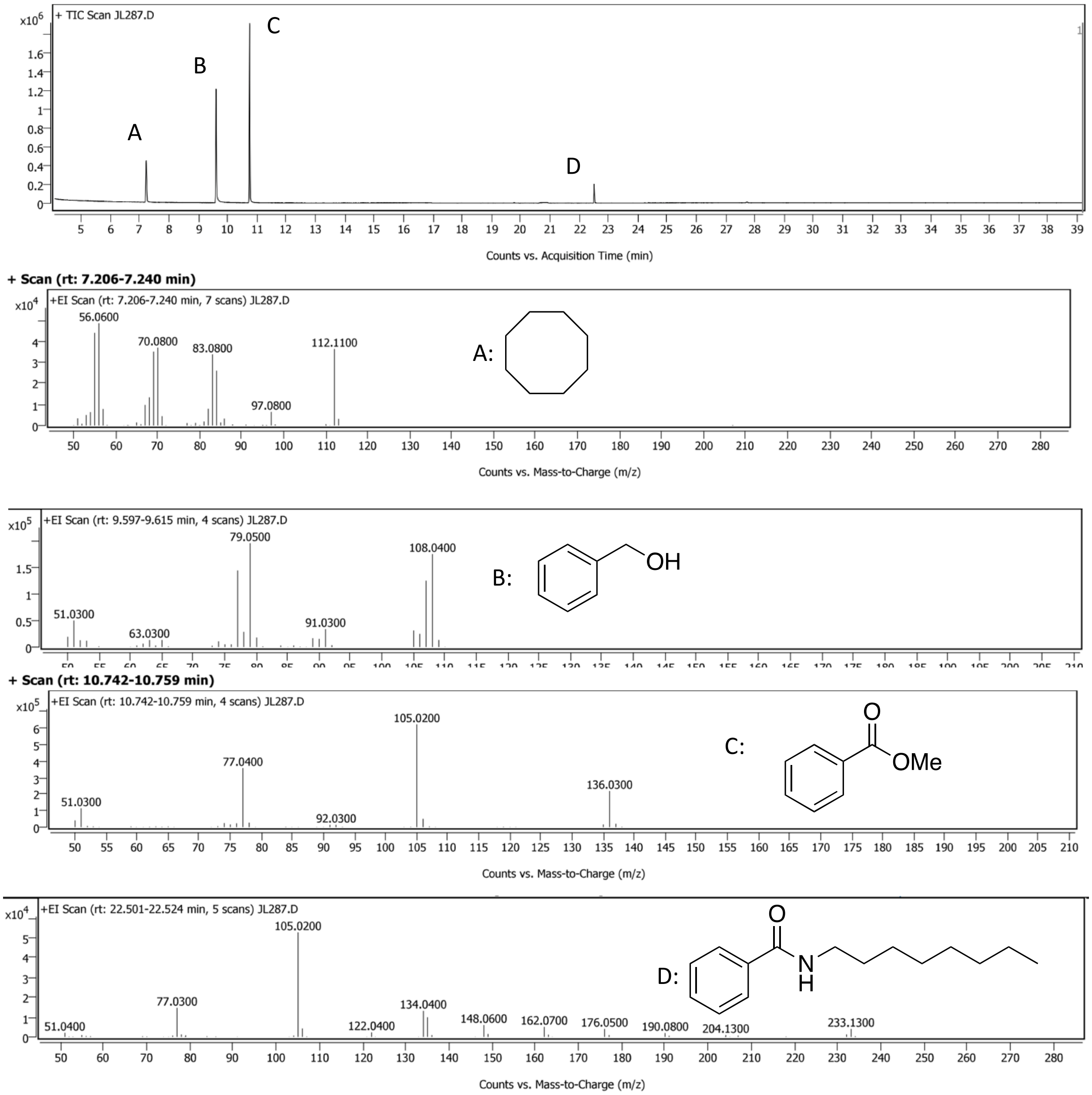

Figure S7: GCMS spectrum of hydrogenation of methyl benzoate in the presence of N-octylbenzamide (10 mol%).

3.4. Kinetic plots for the hydrogenation of esters in the presence of various inhibitors

The central blue line is the average of two trials in the following diagrams.

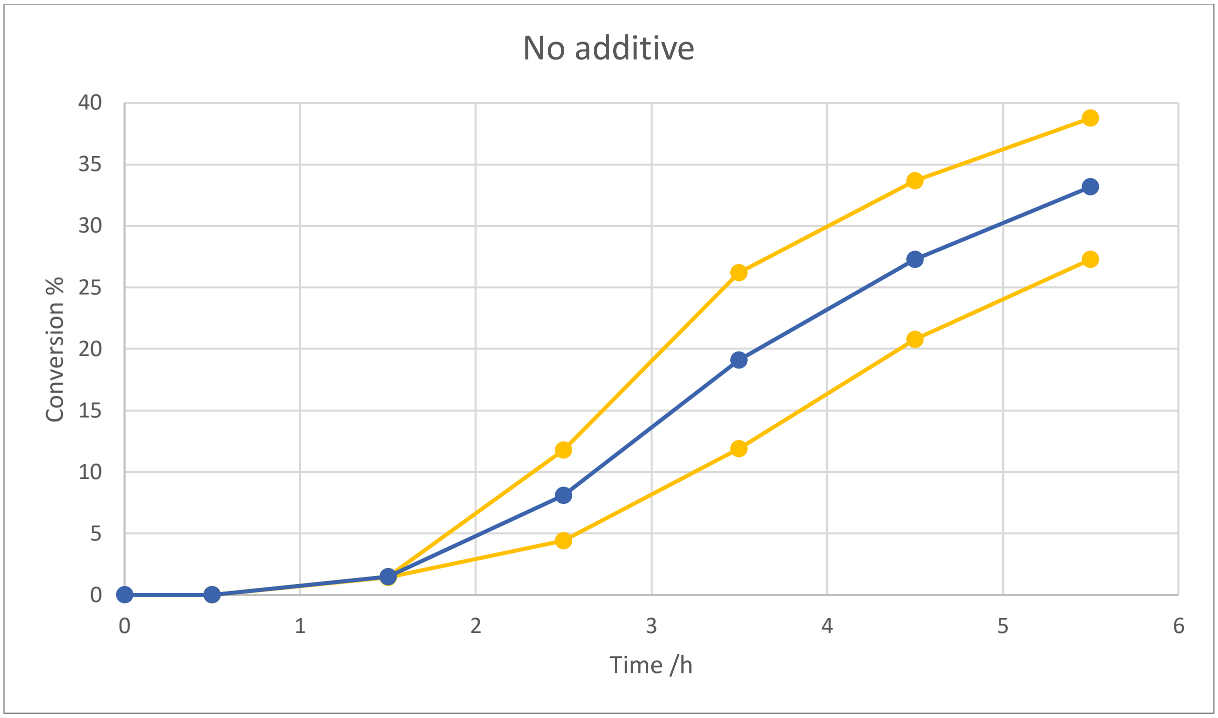

Figure S8: Kinetic graph of hydrogenation of methyl benzoate following general procedure A with no additive present (Table S1, entry 1).

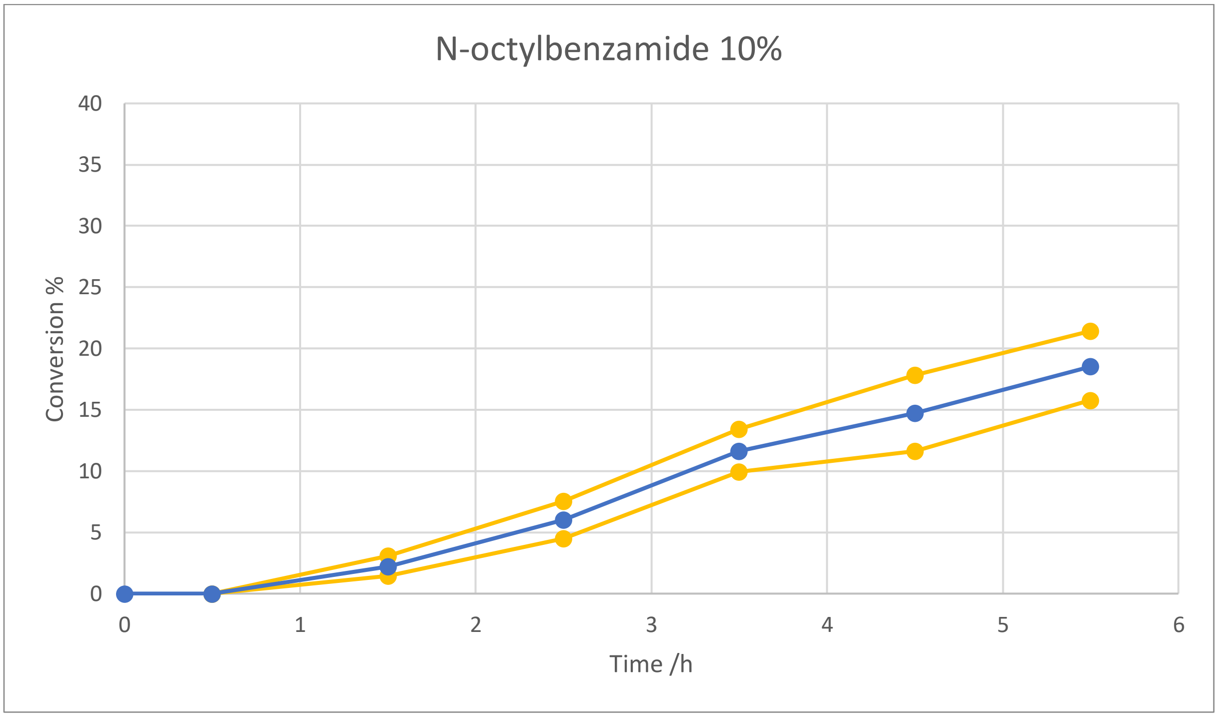

Figure S9: Kinetic graph of hydrogenation of methyl benzoate following general procedure A in the presence of N-octylbenzamide (10 mol%) (Table S1, entry 2).

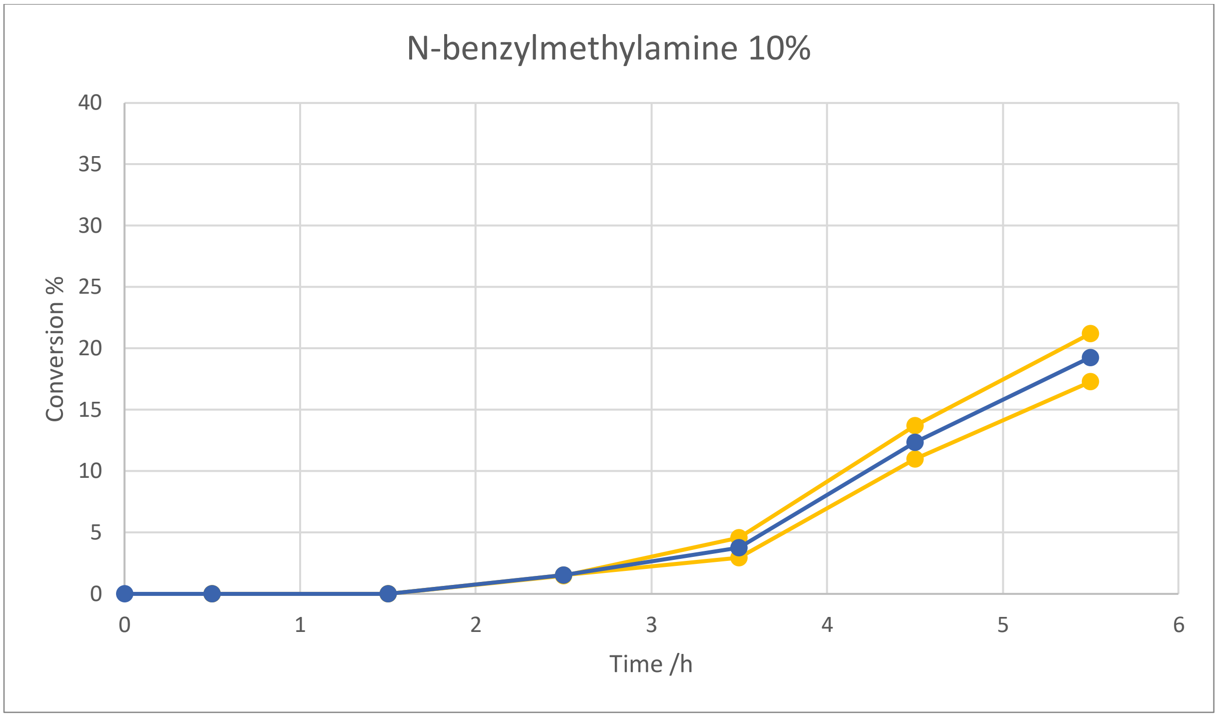

Figure S10: Kinetic graph of hydrogenation of methyl benzoate following general procedure A in the presence of N-benzylmethylamine (10 mol%) (Table S1, entry 3).

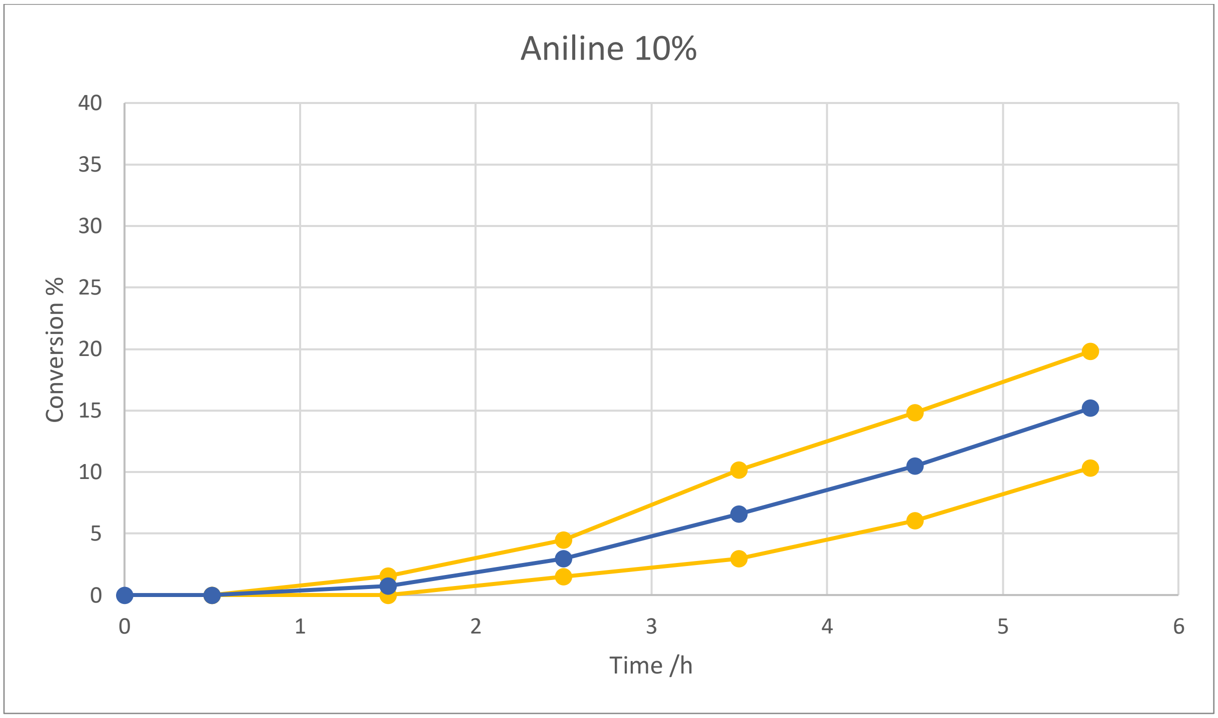

Figure S11: Kinetic graph of hydrogenation of methyl benzoate following general procedure A in the presence of aniline (10 mol%) (Table S1, entry 4).

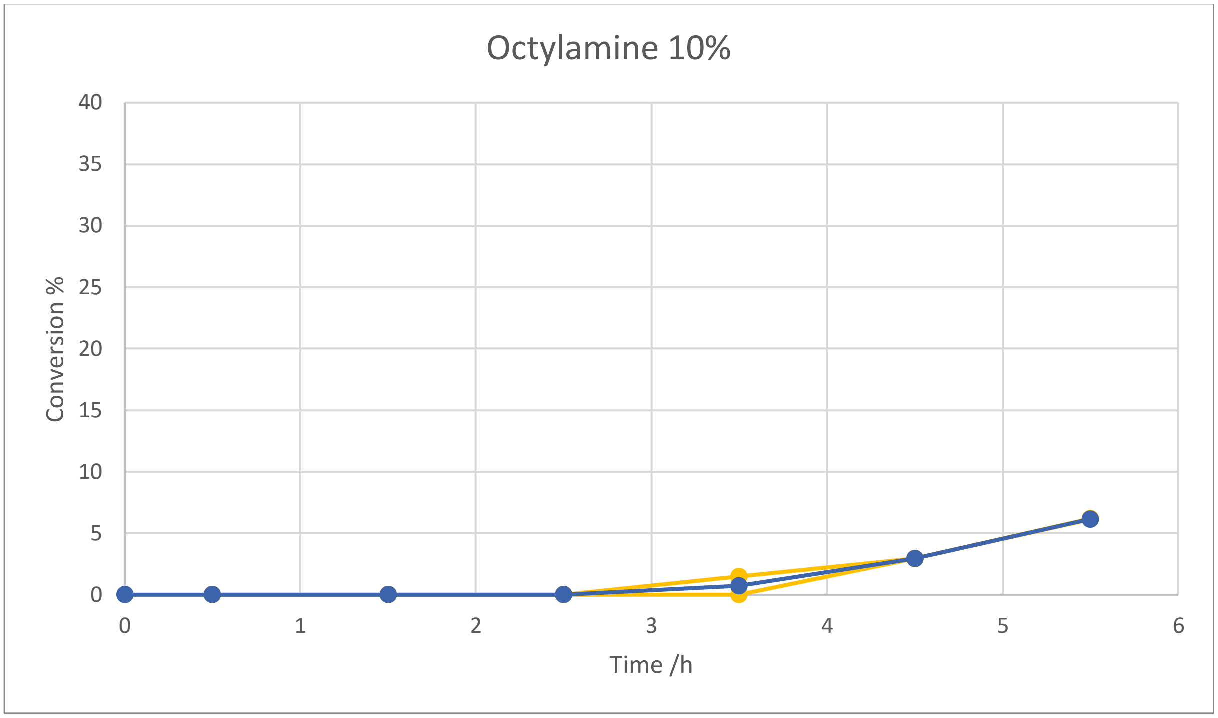

Figure S12: Kinetic graph of hydrogenation of methyl benzoate following general procedure A in the presence of octylamine (10 mol%) (Table S1, entry 5).

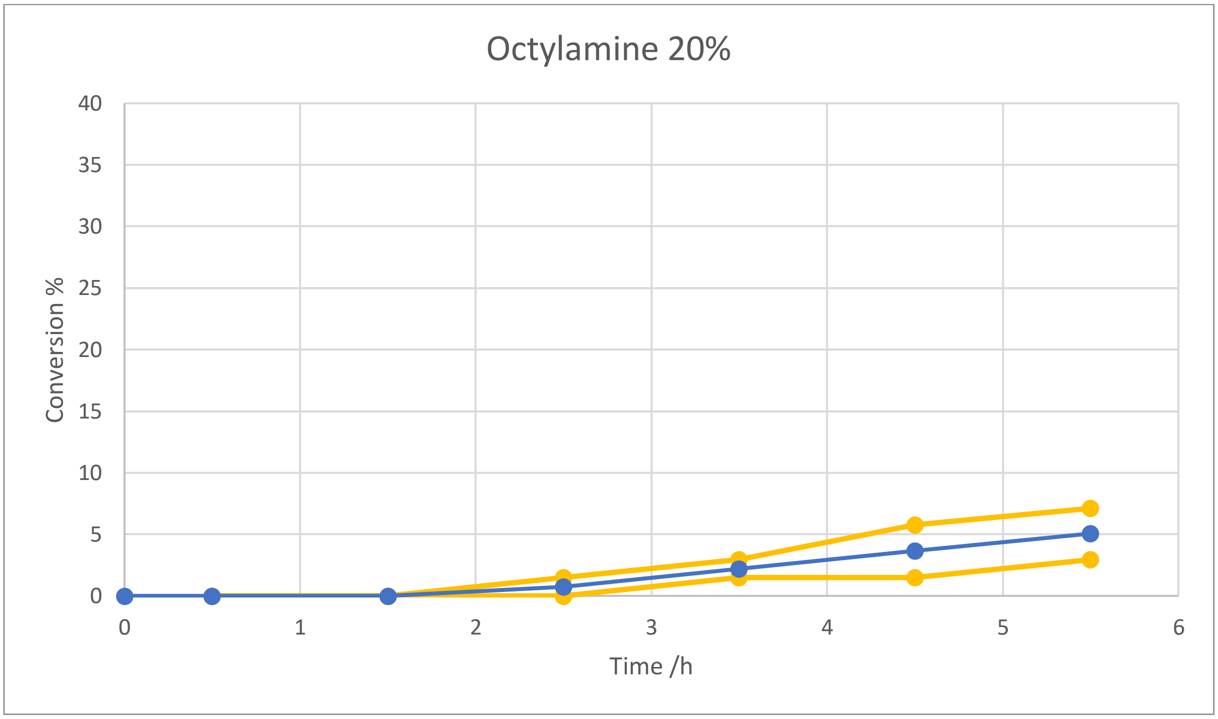

Figure S13: Kinetic graph of hydrogenation of methyl benzoate following general procedure A in the presence of octylamine (20 mol%) (Table S1, entry 6).

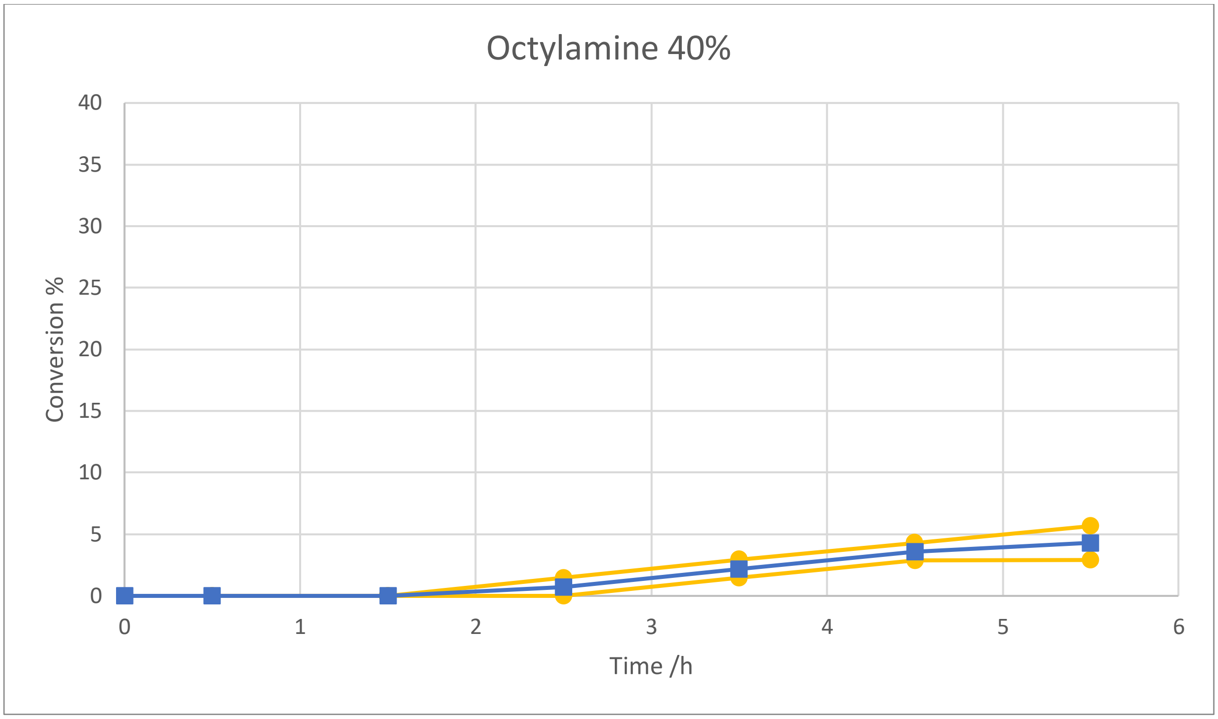

Figure S14: Kinetic graph of hydrogenation of methyl benzoate following general procedure A in the presence of octylamine (40 mol%) (Table S1, entry 7).

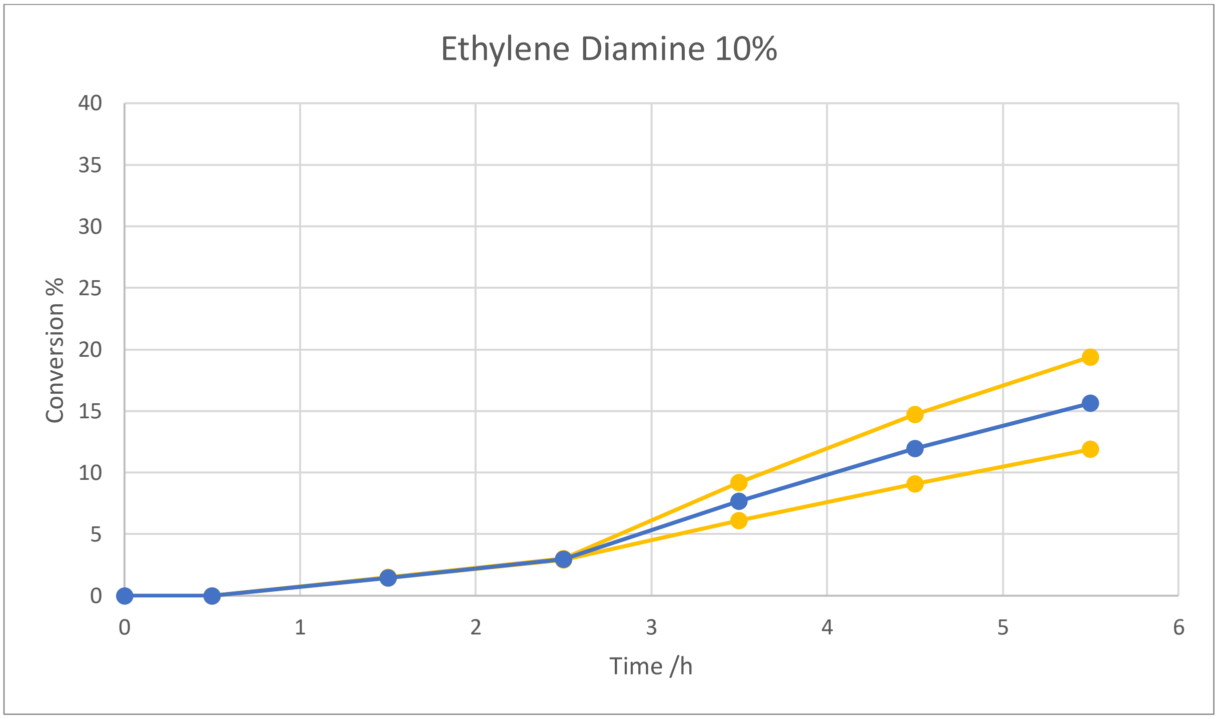

Figure S15: Kinetic graph of hydrogenation of methyl benzoate following general procedure A in the presence of ethylene diamine (10 mol%) (Table S1, entry 8).

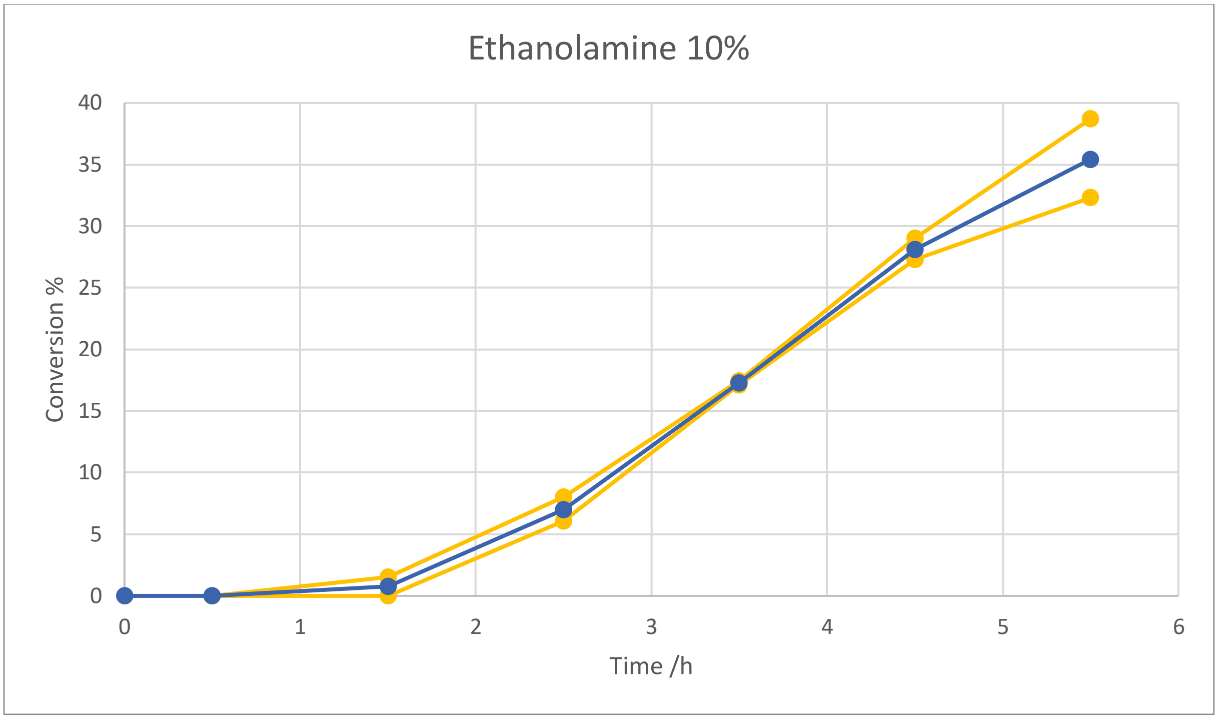

Figure S16: Kinetic graph of hydrogenation of methyl benzoate following general procedure A in the presence of ethanolamine (10 mol%) (Table S1, entry 9).

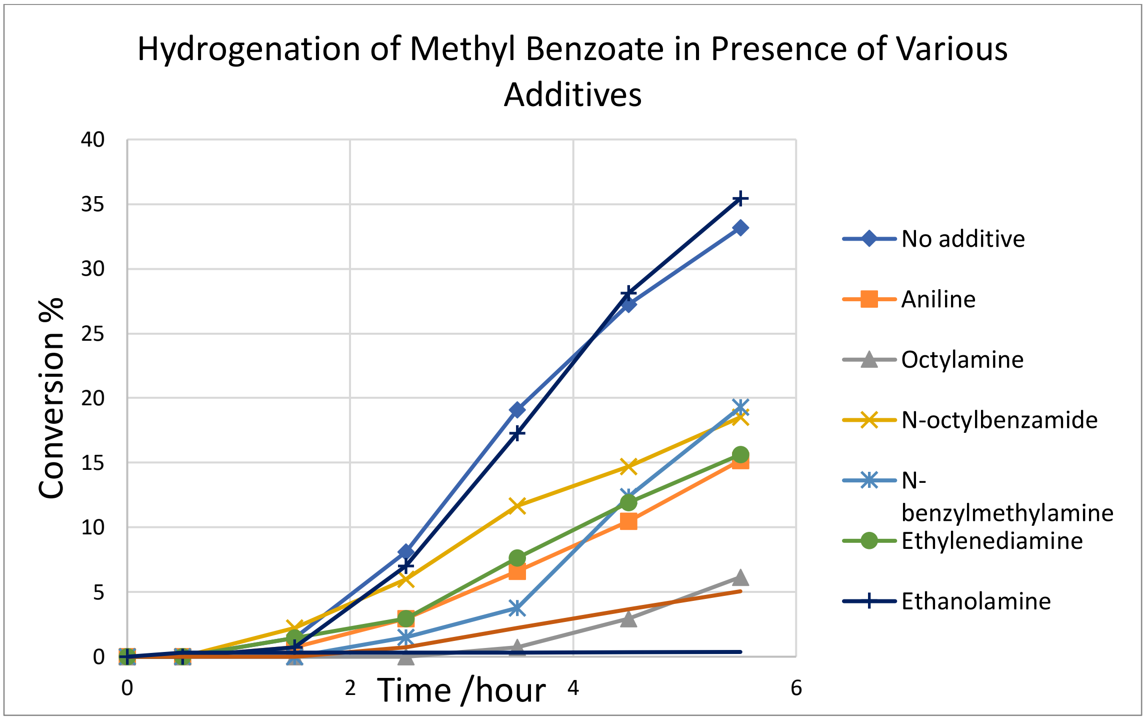

Figure S17: Kinetic graph of hydrogenation of methyl benzoate following general procedure A in the presence of various additives over 5.5 hours.

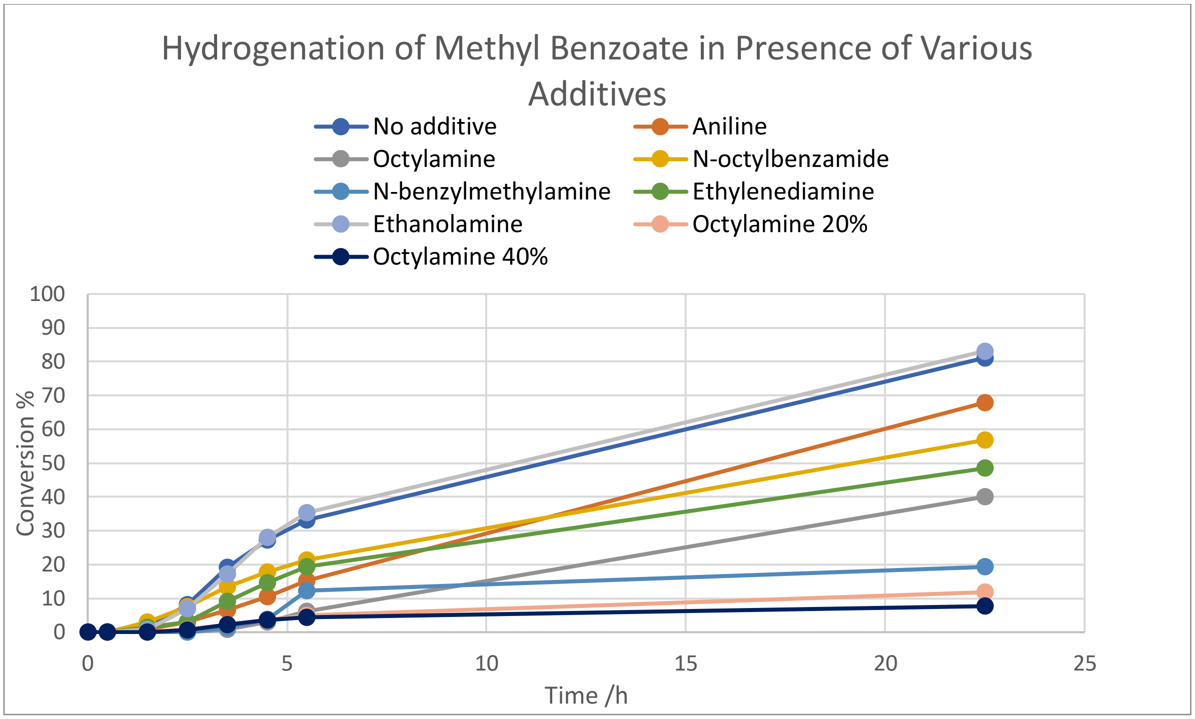

Figure S18: Kinetic graph of hydrogenation of methyl benzoate following general procedure A in the presence of various additives over 22.5 hours.

4. High pressure infrared spectroscopy

4.1. General procedure for HPIR spectroscopy

High pressure infrared spectroscopy was performed in a Parr high pressure IR CSTR vessel constructed from Hastelloy C, fitted with CaF_2 windows and rated to 275 bar. The adjustable path length was set to 4 mm. The high pressure IR spectra were recorded using an Avatar 360 FT-IR. The equipment has been described in a previous publication.[3]

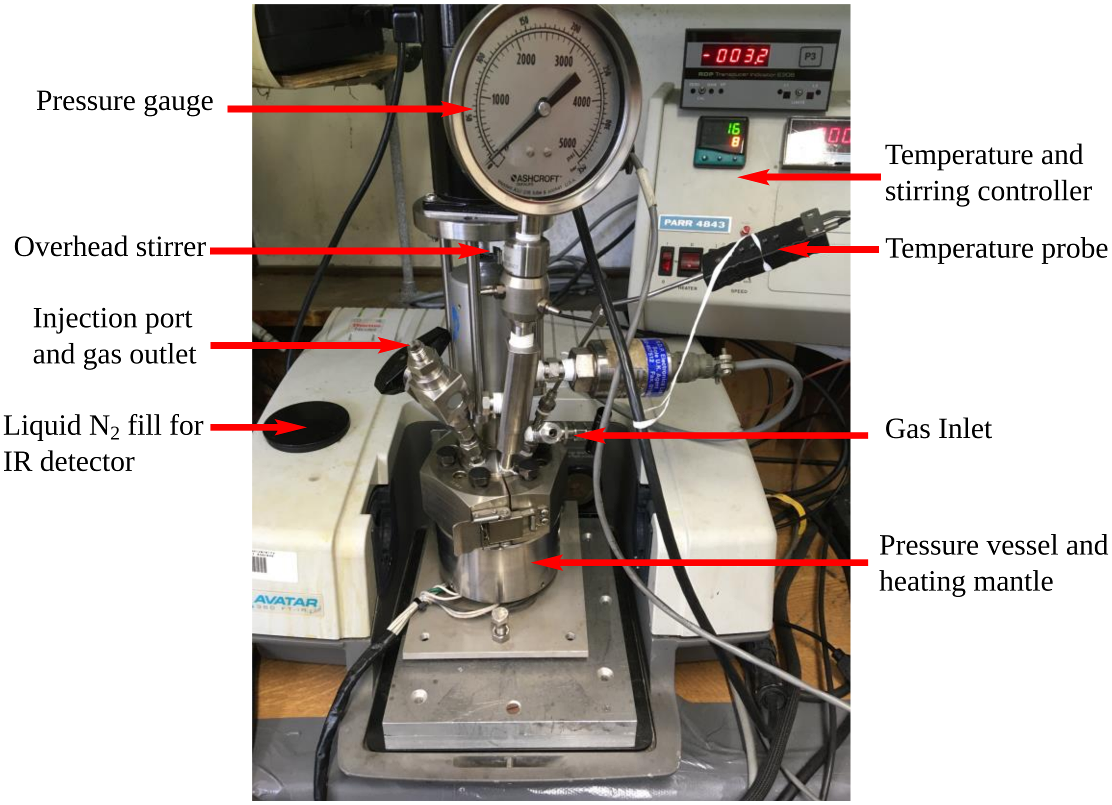

Figure S19. High pressure infrared spectroscopy apparatus

Procedure for monitoring the reaction of Manganese complex 1 with base and octylamine in the presence of Hydrogen using HPIR spectroscopy.

The HPIR spectroscopy pressure vessel was purged with H_2 three times and then dichlomethane (17 mL) was injected into the pressure vessel. The heating jacket was set to 38 °C. Once the required temperature was reached, the vessel was pressurised by 20 bar of H_2 and a background spectrum was recorded (512 scans). Mn complex **1**, (38 mg, 0.05 mmol) was added to a flame-dried Schlenk tube, which was then purged with nitrogen. DCM (3 mL) was added to dissolve the Mn complex **1** and stirred for 10 minutes. The solution containing complex **1** was then transferred *via* syringe to the pressure vessel (which had been depressurised before addition) through the injection port. H_2 (20 bar) was added and the IR spectrum was recorded, 128 scans per spectrum. The starting complex displayed bands at 2034, 1927 and 1849 cm^{-1} as reported previously using solid-state IR spectroscopy,[4] alongside unidentified smaller bands at 2011 and 1954 cm^{-1}. *t*BuOK, (0.5 mL (1M in *t*BuOH), 0.5 mmol) was added to a flame-dried schlenk tube, which was then purged with nitrogen. DCM (2 mL) was added to dilute the base. The base solution was then transferred *via* syringe to the pressure vessel (which had been depressurised before addition) through the injection port. H_2 (20 bar) was added and the IR spectrum was recorded, 128 scans per spectrum. The

reaction progress was monitored by IR spectroscopy (as described in Figure 3, main manuscript).

Octylamine, (0.33 mL, 2 mmol) was added to a flame-dried Schlenk tube, which was then purged with nitrogen. DCM (2 mL) was added and the amine solution was then transferred *via* syringe to the pressure vessel (which had been depressurised before addition) through the injection port. H_2 (20 bar) was added and the IR spectrum was recorded, 128 scans per spectrum (as described in Figure 3, main manuscript). A similar procedure was used using aniline as octylamine.

4.2. HPIR spectroscopic study of Mn complex 1 in the presence of base and D_2

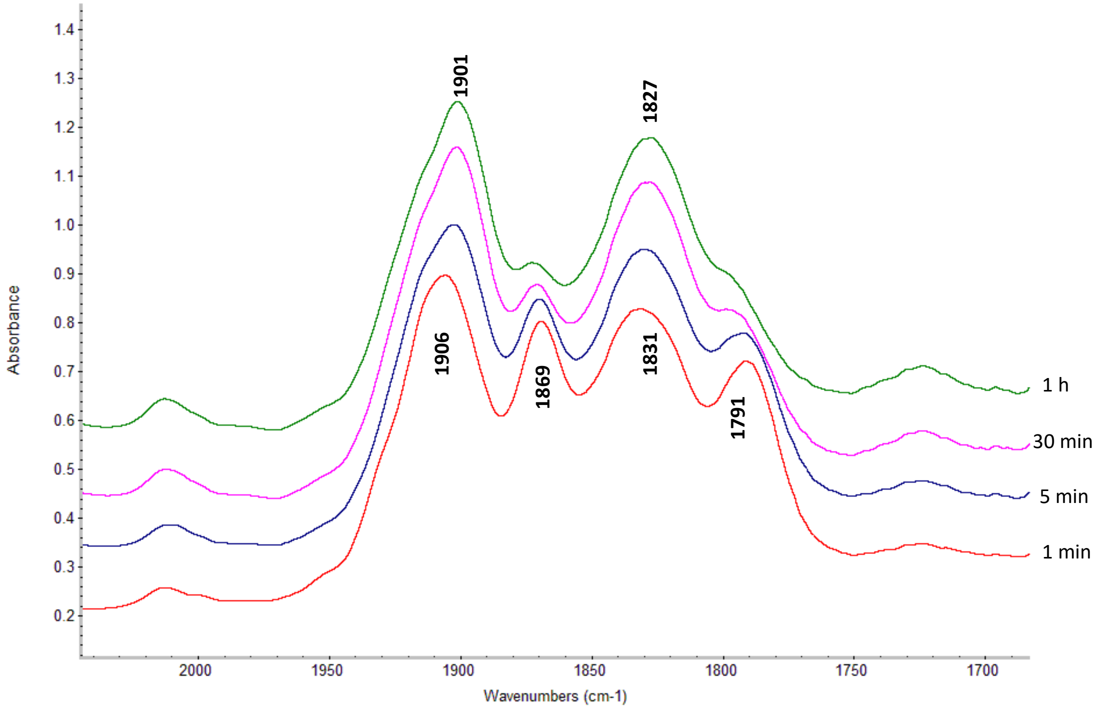

Figure S20: HPIR spectra of **1** in DCM with KO^{t}Bu using D_2. Conditions: as described in the general procedure, T = 38 °C, = 10 bar, D_2.

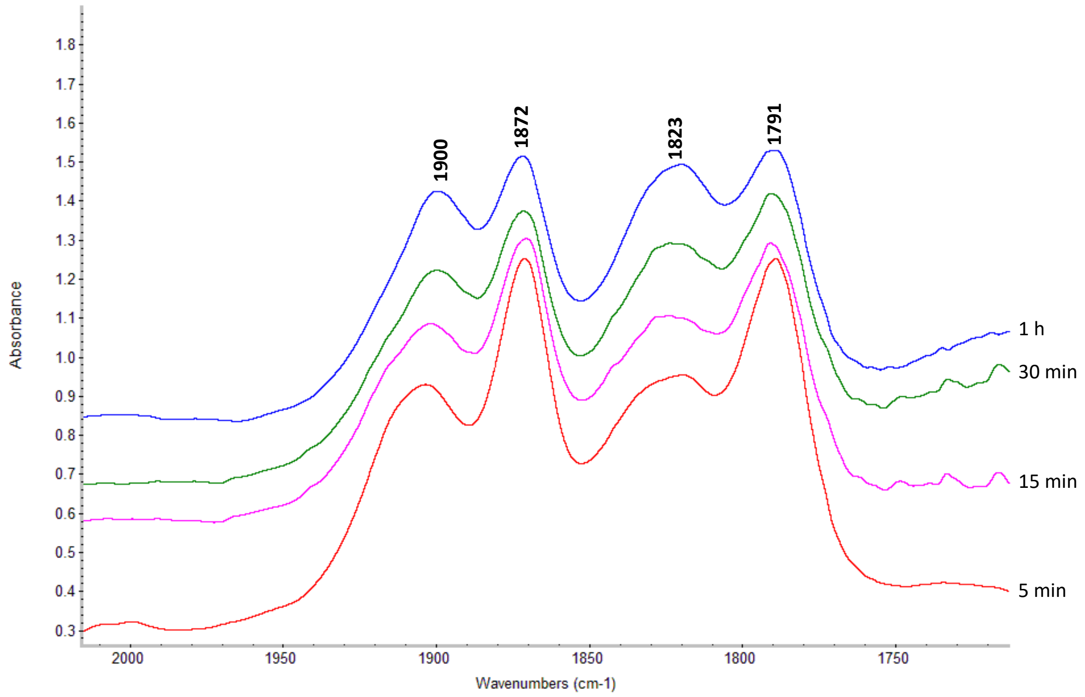

Figure S21: HPIR spectra of **1** in DCM with KO^{t}Bu using H_2. Conditions: as described in the general procedure, T = 38 °C, = 10 bar, D_2.

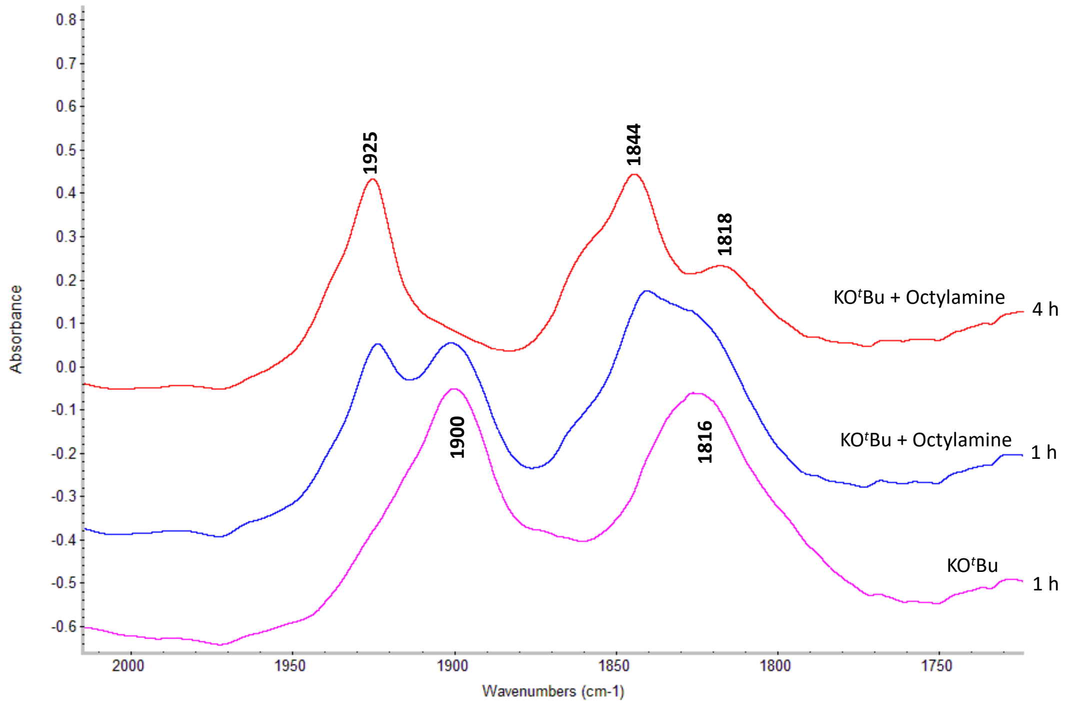

Figure S22: HPIR spectra of **1** in DCM with KO^{t}Bu and then octylamine (using D_2). Conditions: as described in the general procedure, T = 38 °C, = 10 bar, D_2.

5. Control experiment of hydrogenation of methyl benzoate in presence of DCM

5.1. General procedure for hydrogenation of methyl benzoate in presence of DCM

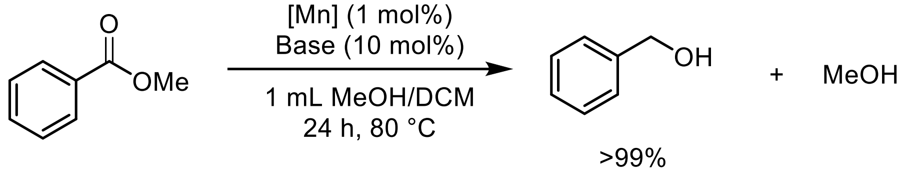

Complex **1** (7.6 mg, 0.01 mmol) was added to a microwave vial. The vial was evacuated and purged with nitrogen three times before adding methyl benzoate (126 µL, 1 mmol), cyclooctane (28 µL, 0.25 mmol), methanol (0.5 mL) and DCM (0.5 mL). Finally, potassium *t*-butoxide (0.1 mL, 1 mol dm^{-3} (in *t*-butanol), 0.1 mmol) was added to the vessel. The vial was then pierced with two needles and placed in an autoclave that had been vacuum cycled 3 times with nitrogen. The autoclave was then purged 3 times with hydrogen before being filled to the desired hydrogen pressure (50 bar). The yield (>99%) of the product (benzyl alcohol) was assigned in the same way as section 3.

5.2. NMR and GCMS data for hydrogenation of methyl benzoate in presence of DCM

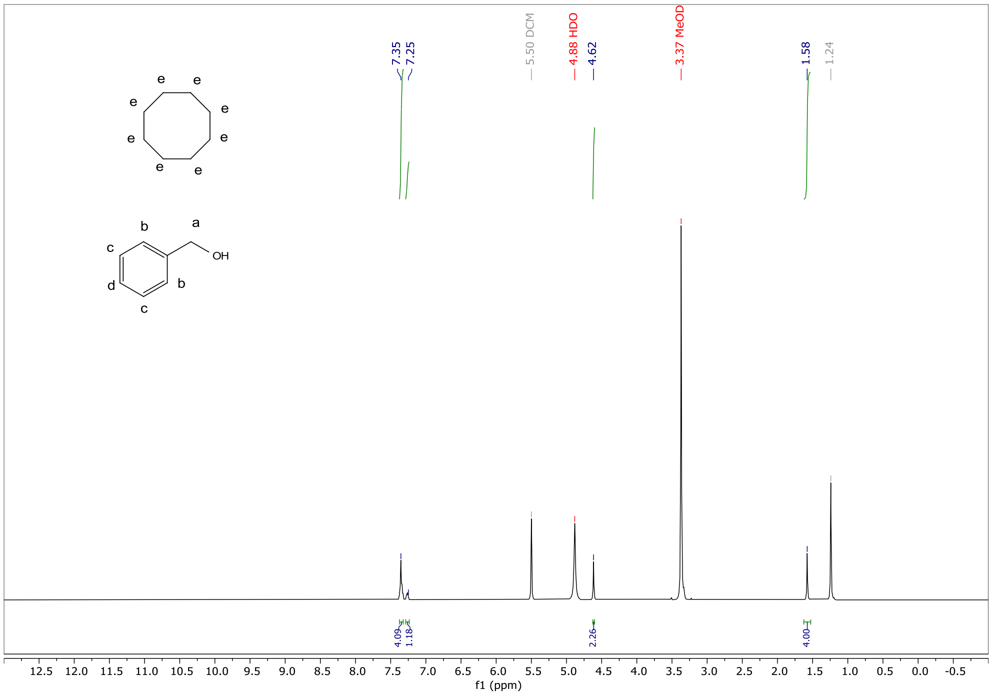

Figure S23: ^1H NMR spectrum (CD_3OD, 298K) of hydrogenation of methyl benzoate in the presence of DCM.

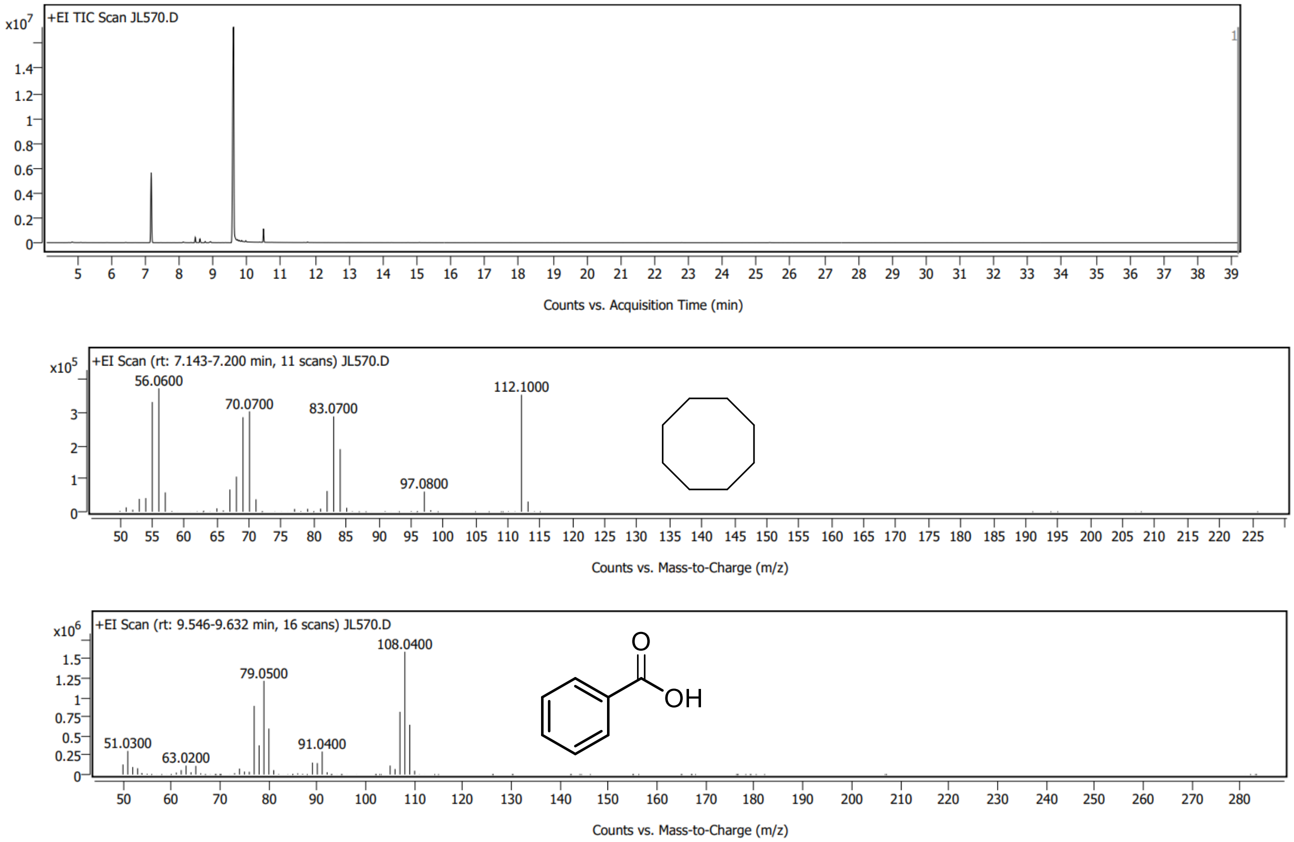

Figure S24: GCMS spectra of hydrogenation of methyl benzoate in the presence of DCM.

6. Hydrogenation of amides

6.1. General procedures used in the hydrogenation of amides

General procedure for the preparation of benzanilide and N-octylbenzamide

Triethylamine (1464 µL, 10.5 mmol) and the desired amine (10.0 mmol) in DCM (20 mL) were added to benzoyl chloride (1220 µL, 10.5 mmol) dropwise before being allowed to stir for 16 hours. The mixture was then quenched with water and extracted into ethyl acetate. The organic phase was washed with 1M $HCl_{(aq)}$, saturated aqueous sodium bicarbonate, and then with brine. It was then dried over magnesium sulfate and the solvent was removed *in vacuo*. The crude residue was washed with hexane and filtered to afford the pure product.

General procedure for the hydrogenation of amides

Amide substrate (0.25 mmol), potassium carbonate (6.9 mg, 0.05 mmol), and complex **1** (3.8 mg, 0.005 mmol, 2 mol%) were added to a flame-dried microwave vial before the vial was evacuated and purged

with argon. Ethanol (1 mL) was then added to the vial before the vial was pierced with two needles and added to an autoclave, which had already been purged with argon. The autoclave was charged with 50 bar of H_2 pressure, before being heated at 65 ℃ and allowed to stir for 16 hours. The conversions of the substrates to the products and their yields were then calculated by the ^{1}H NMR spectroscopy using the integrals of signals attributed to the product relative to those attributed to the reactant, using 1,4-dimethoxybenzene as an internal standard. Isolated amines were purified by flash column chromatography (DCM:MeOH, 95:5).

6.2. Optimisation studies for the catalytic hydrogenation of benzanilide

Table S3. Optimisation studies for the catalytic hydrogenation of benzanilide

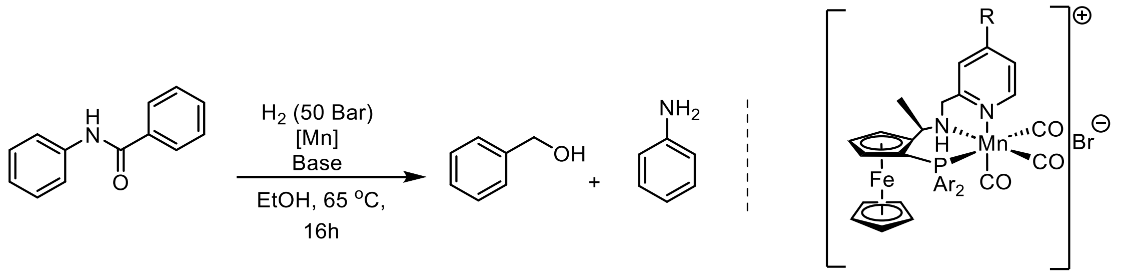

Entry	Cat./ loading (mol%)	Base/ loading (mol%)	T (°C)	t (h)	Conc. (M)	Conversion (%)	BnOH (%)	$PhNH_2$ (%)
**1**	**1**/1 mol%	KO^{t}Bu/10	65	16	1	100	>99	>99
**2**	**2**/1 mol%	KO^{t}Bu/10	65	16	1	70	70	70
**3**	**1**/1 mol%	K_2CO_3/10	65	16	1	100	>99	>99
**4**	**1**/0.5 mol%	K_2CO_3/10	65	16	1	50	50	50
**5**	**1**/0.25 mol%	K_2CO_3/10	65	16	1	35	35	35
**6**	**1**/0.75 mol%	K_2CO_3/10	65	16	1	90	90	90
**7**	**1**/0.75 mol%	K_2CO_3/10	65	16	0.5	85	85	85

**8**	**1**/1 mol%	K_2CO_3/10	50	16	1	40	40	40

Table S4: Hydrogenation of bezanilide in presence of additives

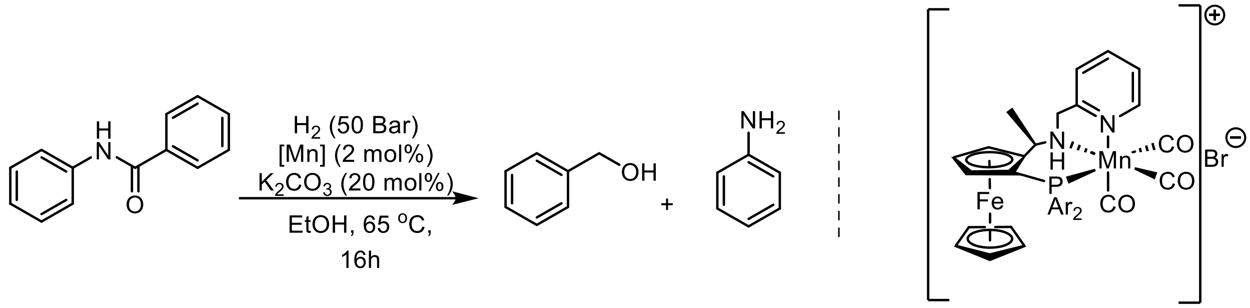

Entry	Additive (40 mol%)	Benzyl alcohol yield (%)	Aniline yield (%)
**1**	None	>99	99
**2**[a]	Aniline	83	N/A
**3**	Octylamine	74	68

[a]yield of aniline not given due to use of aniline as additive.

6.3. Hydrogenation of Amides in the presence of manganese complexes

Table S5: Hydrogenation of amides and corresponding crude yields of amines and alcohols in the presence of complex 1

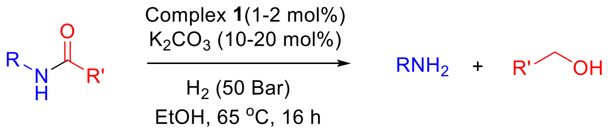

Entry	Amide	Catalyst/Base loading (mol%)	Amine yield (%)	Alcohol Yield (%)
1	Benzanilide (**A**)	1/10	86	95
2	Palmitanilide (**B**)	1/10	31	NA
3	*p*-benzanisidide (**C**)	1/10	12	16
4	4’-bromo-3’-chloroacetanilide (**D**)	1/10	31	NA
5	2’-fluoroacetanilide (E)	1/10	30	NA
6	N-methyldodecanamide (**G**)	1/10	0	0
7	Benzanilide (**A**)	2/20	95 (78)	99
8	Palmitanilide (**B**)	2/20	57	NA
9	*p*-benzanisidide (**C**)	2/20	70 (64)	75
10	4’-bromo-3’-chloroacetanilide (**D**)	2/20	99 (96)	NA
11	2’-fluoroacetanilide (**E**)	2/20	95	NA

12	N-(4-methylpyridin-2-yl)acetamide (**F**)	2/20	100 (100)	NA
13	N-methyldodecanamide (**G**)	2/20	0	0
14	N-octylbenzamide (**H**)	2/20	0	0

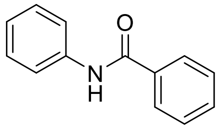

A

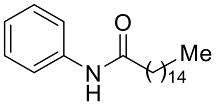

B

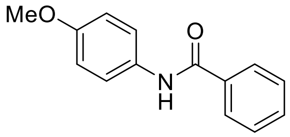

C

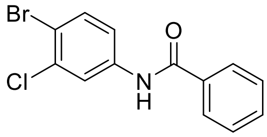

D

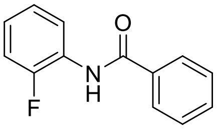

E

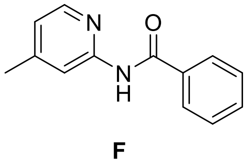

F

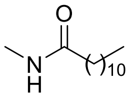

G

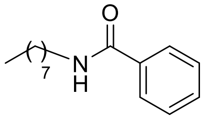

H

Table S6: Attempted hydrogenation of Kevlar®

Kevlar fabric was purchased from Amazon and cut into small pieces before adding to the reactor. The hydrogenation was conducted using the method described in section 4.1 for the hydrogenation of amides.

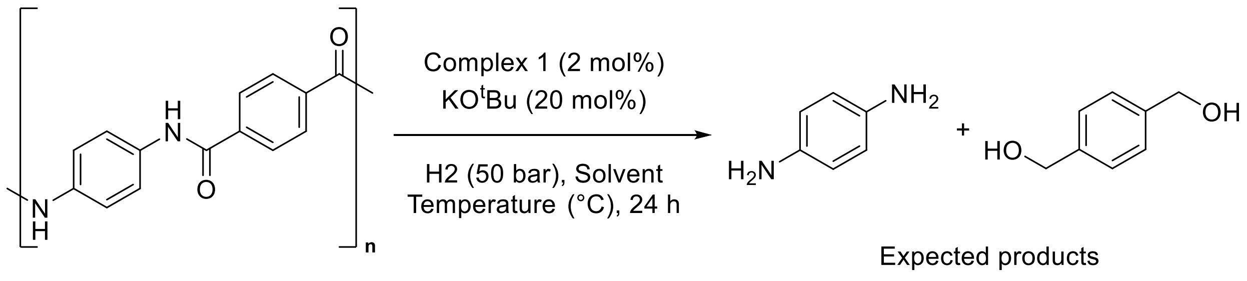

**Entry**	**Base**	**Temperature (℃)**	**Solvent**	**Yield (%)**
**1**	KO^{t}Bu	120	EtOH	0
**2**	KO^{t}Bu	150	EtOH	0
**3**	KO^{t}Bu	150	Ethylene glycol	0
**4**	KO^{t}Bu	165	EtOH	0
**5**	KO^{t}Bu	150	DMSO	0
**6**	KO^{t}Bu	150	NMP	0

6.4. Characterisation data from the hydrogenation of amides in the presence of manganese complex 1

6.4.1. NMR spectral details for the amines obtained from the hydrogenation of amides:

4-methoxy aniline

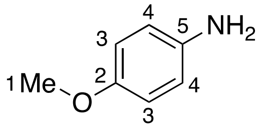

δ_H (400 MHz, $CDCl_3$) 6.75 (2H, d, *J* = 8.5 Hz, H-3), 6.66 (2H, d, *J* = 8.5 Hz, H-4), 3.75 (3H, s, H-1); δ_C (100 MHz, $CDCl_3$) 152.8 (C-2), 139.8 (C-5), 116.4 (C-4), 114.7 (C-3), 55.7 (C-1); GC-MS (EI): *m/z* 120 (M3+), 108 ($OC_6H_4NH_2$)

4-bromo-3-chloroaniline

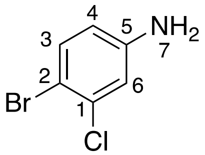

δ_H (400 MHz, $CDCl_3$) 7.31 (1H, d, *J* = 8.6 Hz, H-3), 6.77 (1H, d, *J* = 2.7 Hz, H-6), 6.43 (1H, dd, *J* = 8.6 Hz, 2.7 Hz, H-4), 3.73 (2H, s, H-7); δ_C (100 MHz, $CDCl_3$) 146.6 (C-5), 134.5 (C-3), 133.8 (C-1), 116.3 (C-6), 114.9 (C-4), 109.6 (C-2); GC-MS (EI): *m/z* 207 (M+), 126 ($C_6H_3ClNH_2$), 90 ($C_6H_3NH_2$)

2-amino-4-methylpyridine

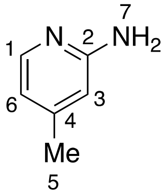

δ_H (400 MHz, $CDCl_3$) 7.88 (1H, d, *J* = 4.2 Hz, H-1), 6.43 (1H, d, *J* = 4.5 Hz, H-6), 6.28 (1H, s, H-3), 4.64 (2H, s, H-7), 2.17 (3H, s, H-5); δ_C (100 MHz, $CDCl_3$) 158.5 (C-2), 148.8 (C-4), 147.3 (C-1), 115.3 (C-6), 108.9 (C-3), 20.8 (C-5); GC-MS (EI): *m/z* 108 (M+), 80 (C_5H_6N)

Aniline

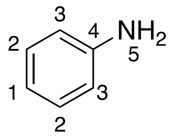

δ_H (400 MHz, $CDCl_3$) 7.19 (2H, m, H-2), 6.80 (1H, m, H-1), 6.71 (2H, m, H-3), 3.61 (2H, s, H-5); δ_C (100 MHz, $CDCl_3$) 146.5 (C-4), 129.3 (C-2), 118.6 (C-1), 115.2 (C-3); GC-MS (EI): *m/z* 93 (M+)

6.4.2. Crude ^{1}H NMR data for the hydrogenation of benzanilide in the presence of various additives

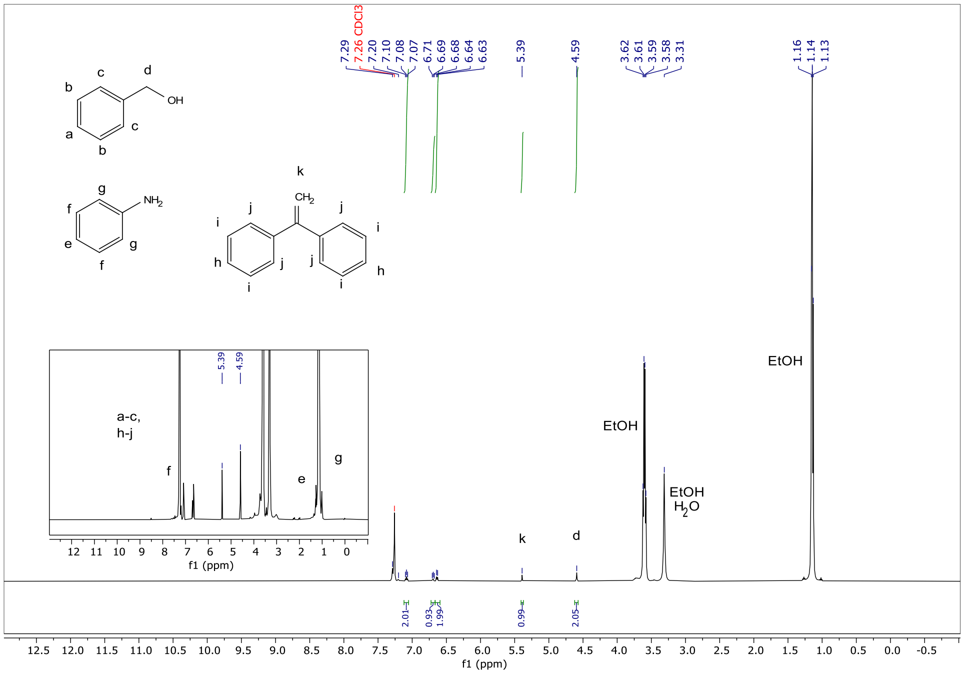

Figure S23: ^{1}H NMR spectrum for hydrogenation of benzanilide with no additive present (Table S4, entry 1).

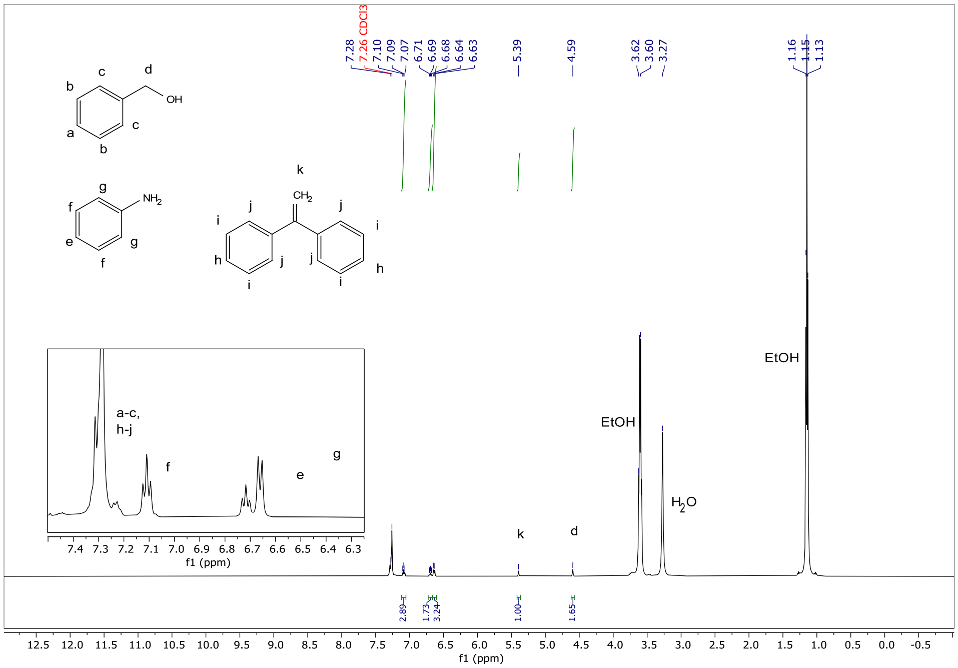

Figure S24: ^{1}H NMR spectrum for hydrogenation of benzanilide in the presence of aniline (Table S4, entry 2).

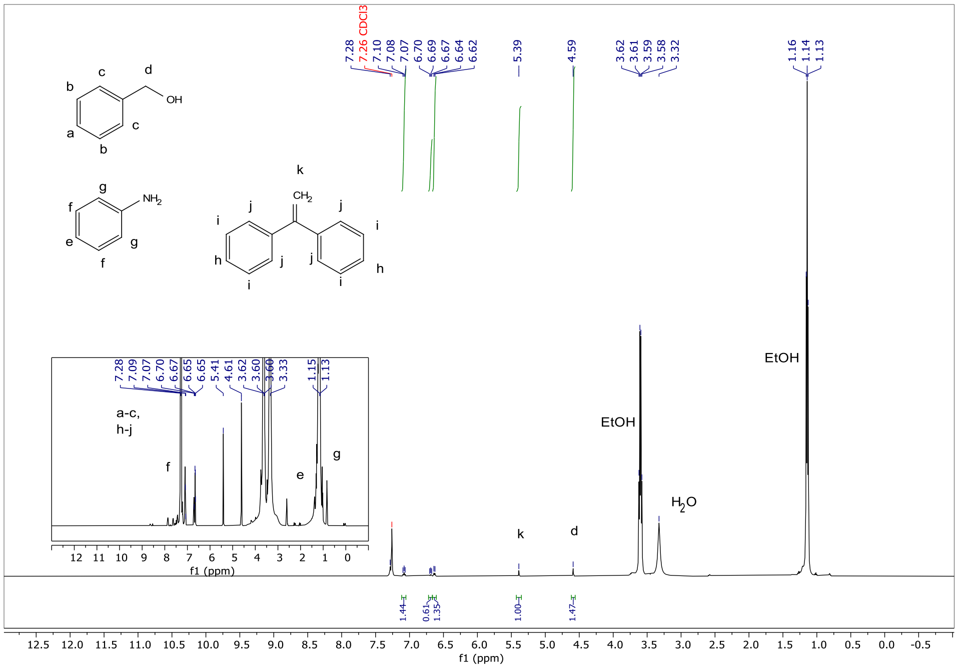

Figure S25: ^{1}H NMR spectrum for hydrogenation of benzanilide in the presence of octylamine (Table S4, entry 3).

6.4.3. Crude ^{1}H NMR data for the hydrogenation of various amides

NMR Spectra for determination of crude yield of amide hydrogenation at 1 mol% [Mn]

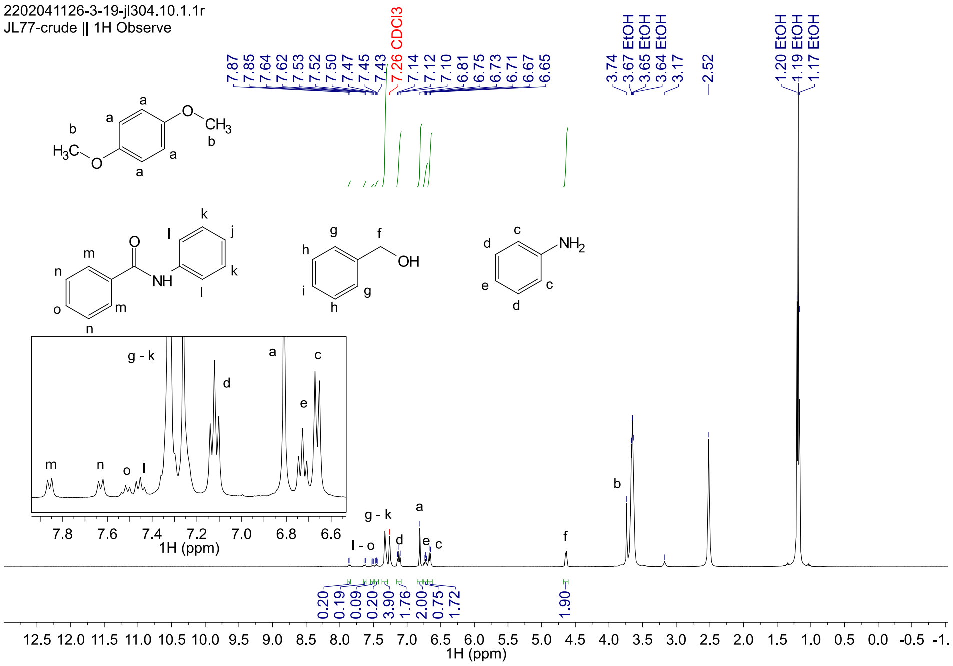

Figure S26: ^{1}H NMR spectrum ($CDCl_3$, 400 MHz, 298 K) of the reaction mixture of hydrogenation of benzanilide with the internal standard (Table S2, entry 1).

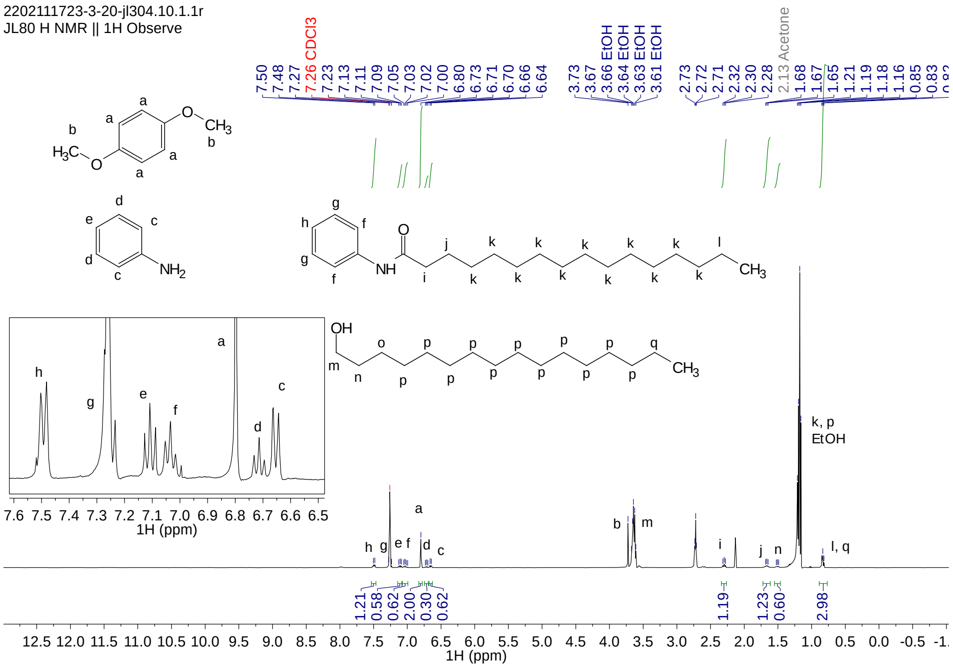

Figure S27: ^{1}H NMR spectrum ($CDCl_3$, 400 MHz, 298 K) of the reaction mixture of hydrogenation of palmitanilide with the internal standard (Table S2, entry 2).

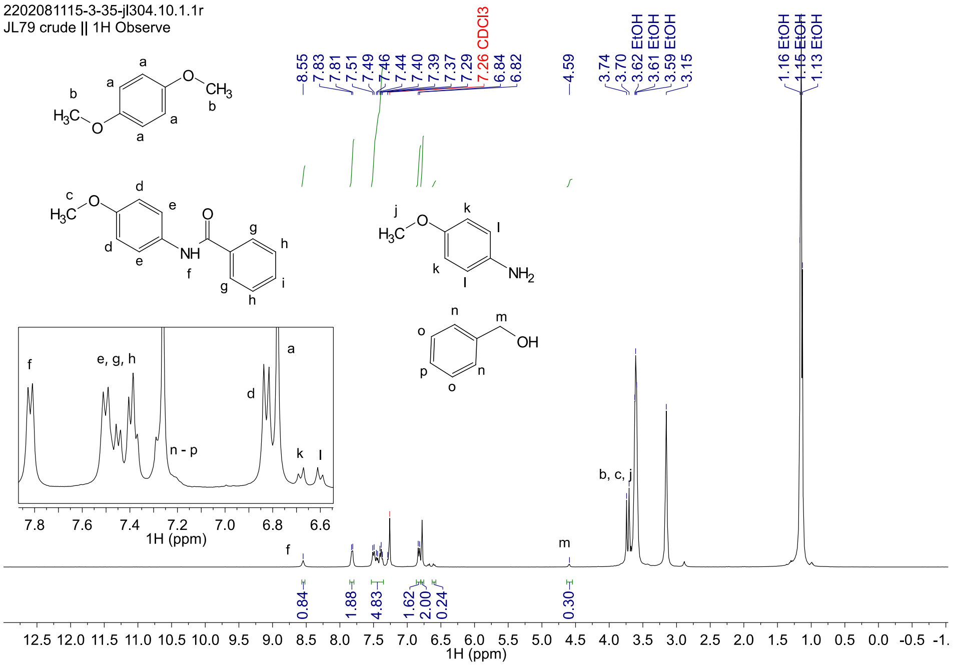

Figure S28: ^{1}H NMR spectrum ($CDCl_3$, 400 MHz, 298 K) of the reaction mixture of hydrogenation of *p*-benzanisidide with the internal standard (Table S2, entry 3).

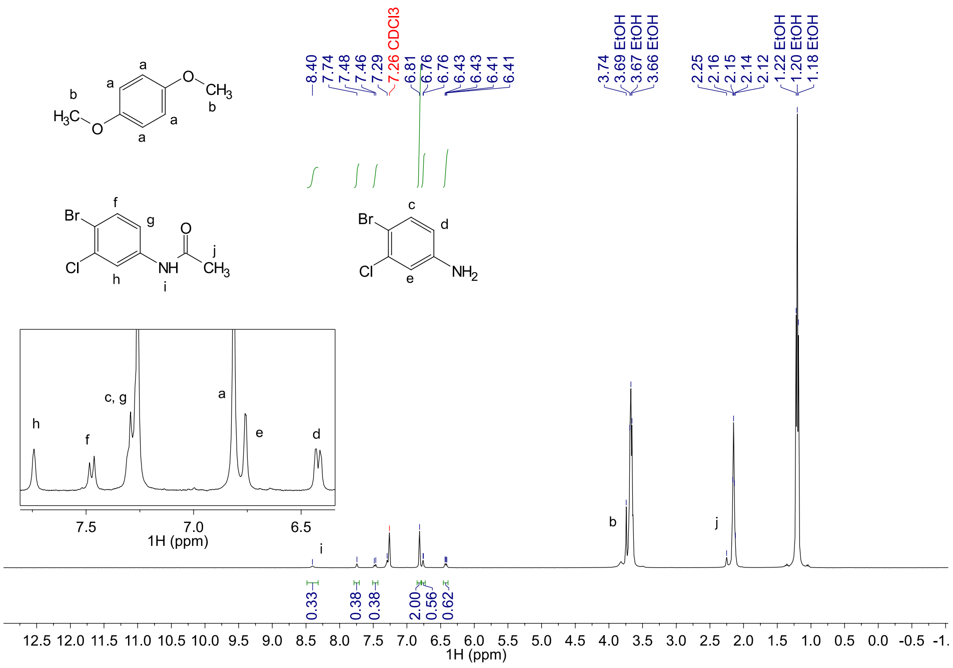

Figure S29: ^{1}H NMR spectrum ($CDCl_3$, 400 MHz, 298 K) of the reaction mixture of hydrogenation of 4'-bromo-3'-chloroacetanilide with the internal standard (Table S2, entry 4).

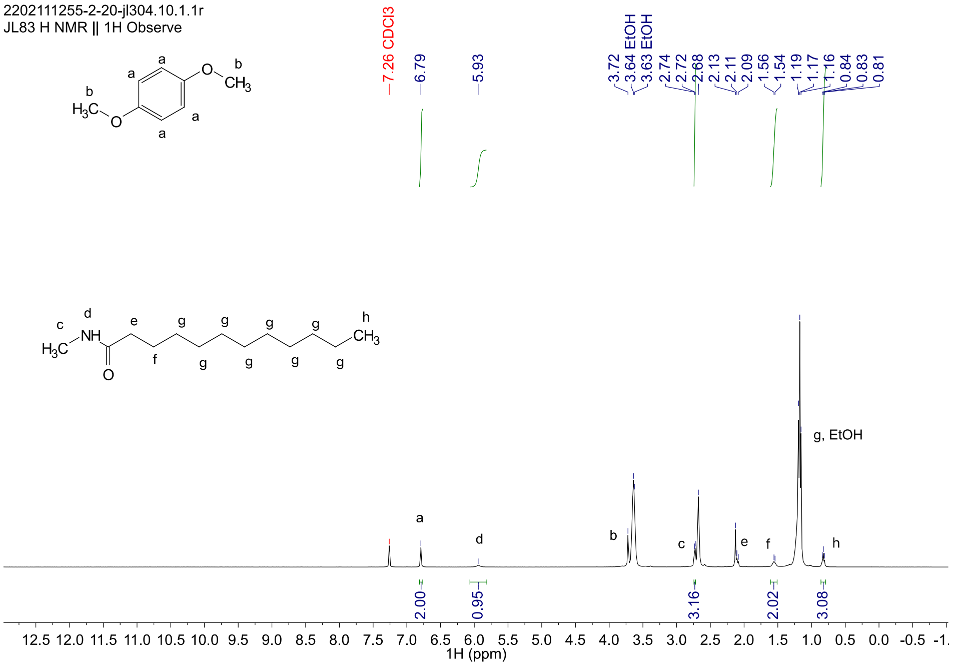

Figure S30: ^{1}H NMR spectrum ($CDCl_3$, 400 MHz, 298 K) of the reaction mixture of hydrogenation of N-methyldodecanamide with the internal standard (Table S2, entry 5).

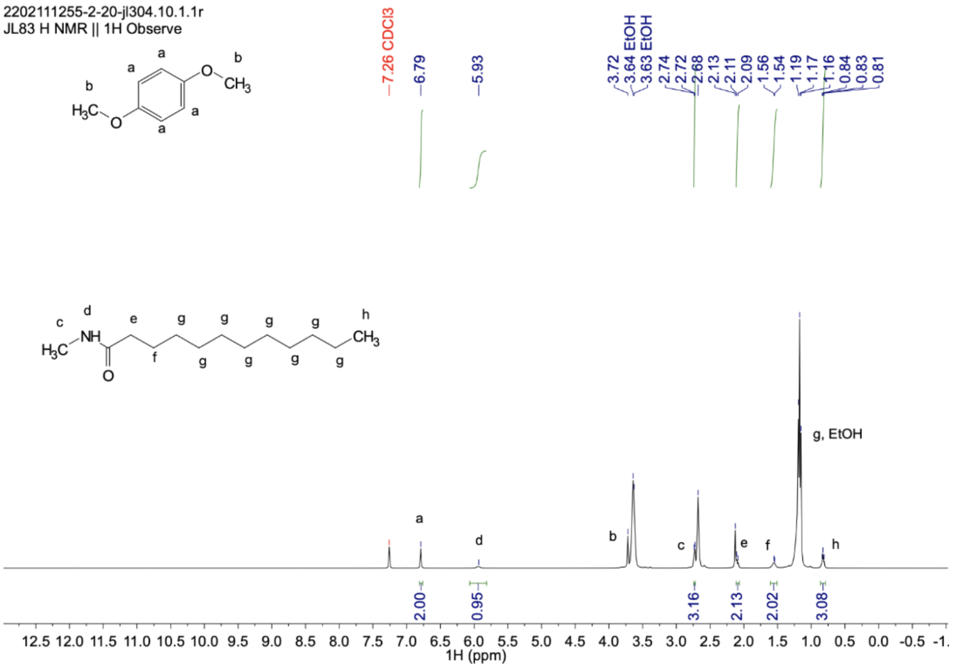

Figure S31: ^{1}H NMR spectrum ($CDCl_3$, 400 MHz, 298 K) of the reaction mixture of hydrogenation of 2'-fluoroacetanilide with the internal standard (Table S2, entry 6).

NMR Spectra for determination of crude yield of amide hydrogenation at 2 mol% [Mn]

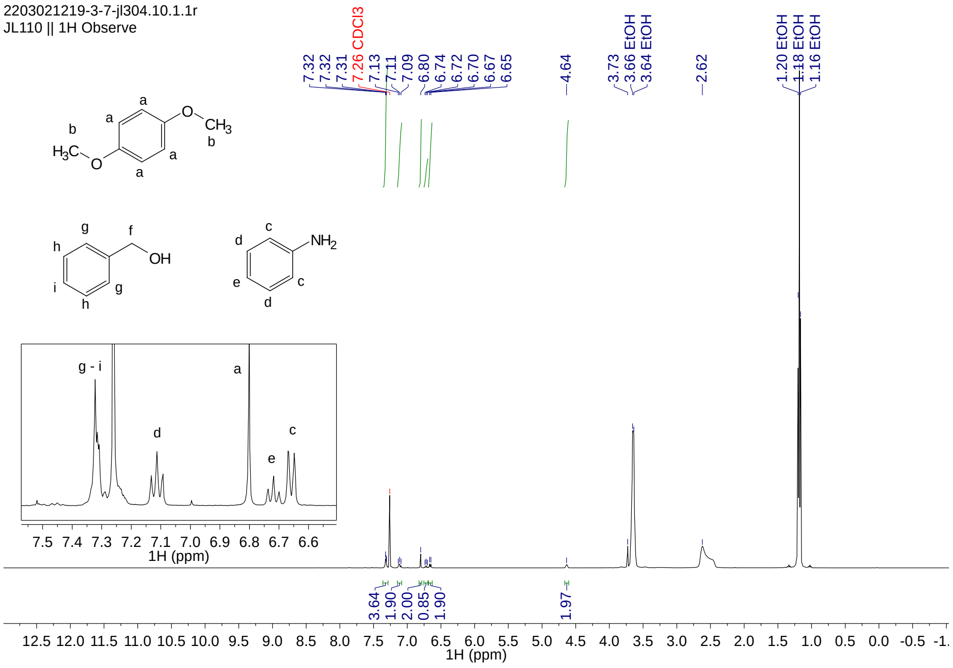

Figure S32: ^{1}H NMR spectrum ($CDCl_3$, 400 MHz, 298 K) of the reaction mixture of hydrogenation of benzanilide with the internal standard (Table S2, entry 7).

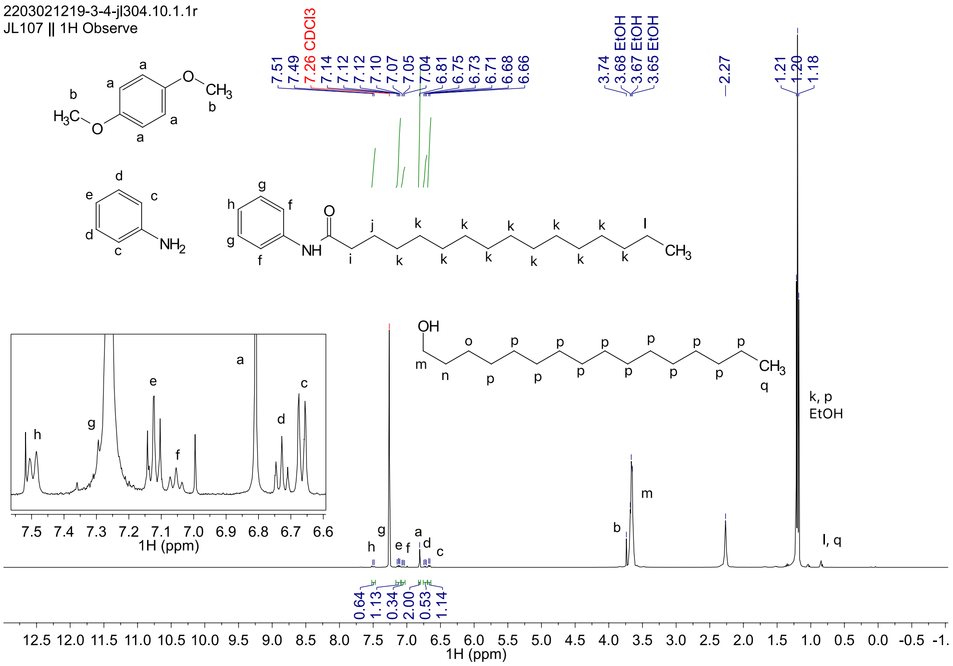

Figure S33: ^{1}H NMR spectrum ($CDCl_3$, 400 MHz, 298 K) of the reaction mixture of hydrogenation of palmitanilide with the internal standard (Table S2, entry 8).

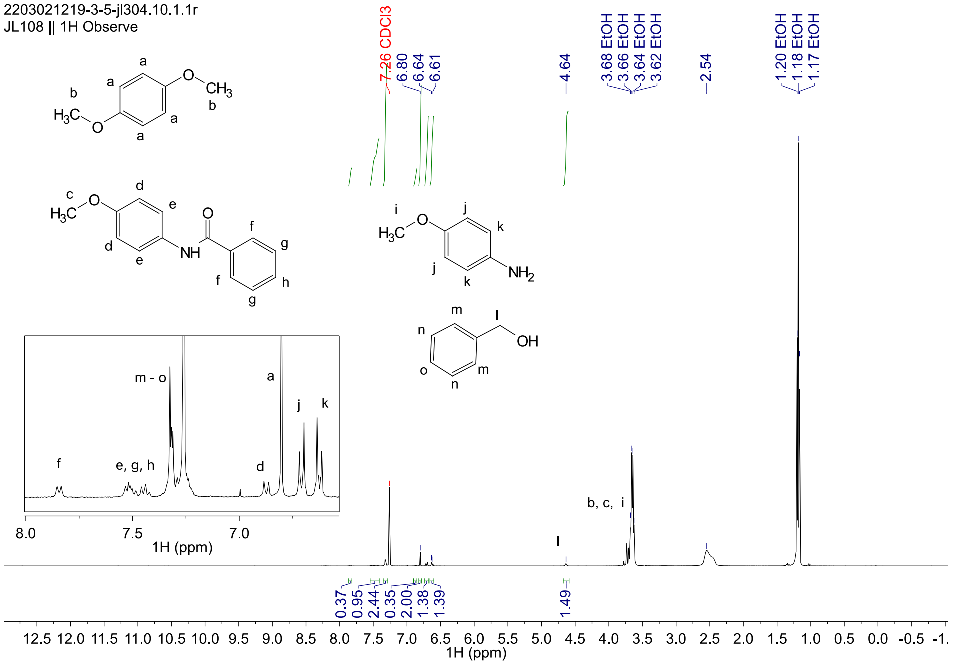

Figure S34: ^{1}H NMR spectrum ($CDCl_3$, 400 MHz, 298 K) of the reaction mixture of hydrogenation of *p*-benzanisidide with the internal standard (Table S2, entry 9).

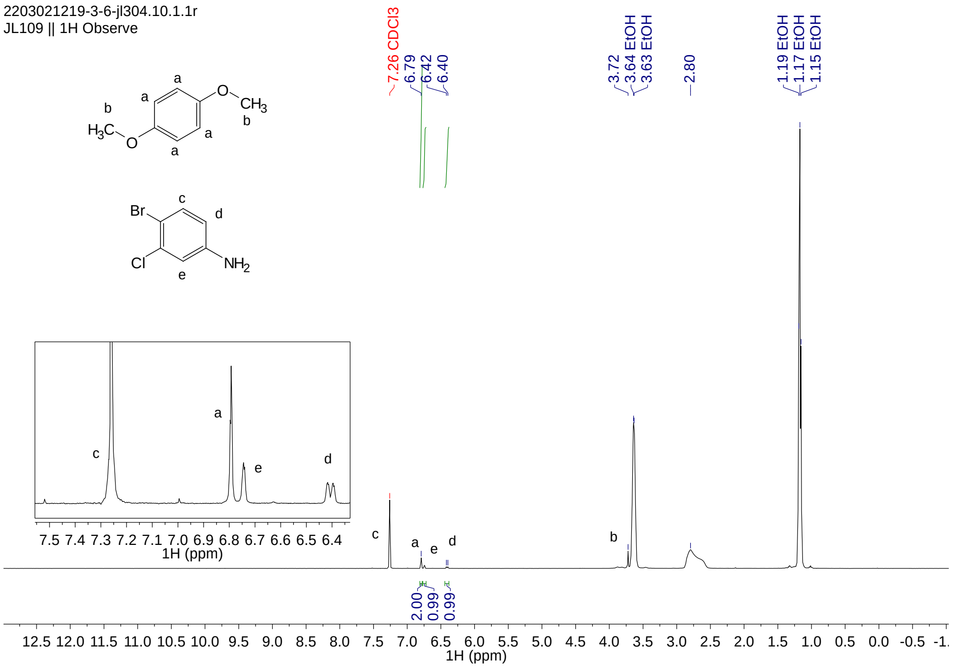

Figure S35: ^{1}H NMR spectrum ($CDCl_3$, 400 MHz, 298 K) of the reaction mixture of hydrogenation of 4’-bromo-3’-chloroacetanilide with the internal standard (Table S2, entry 10).

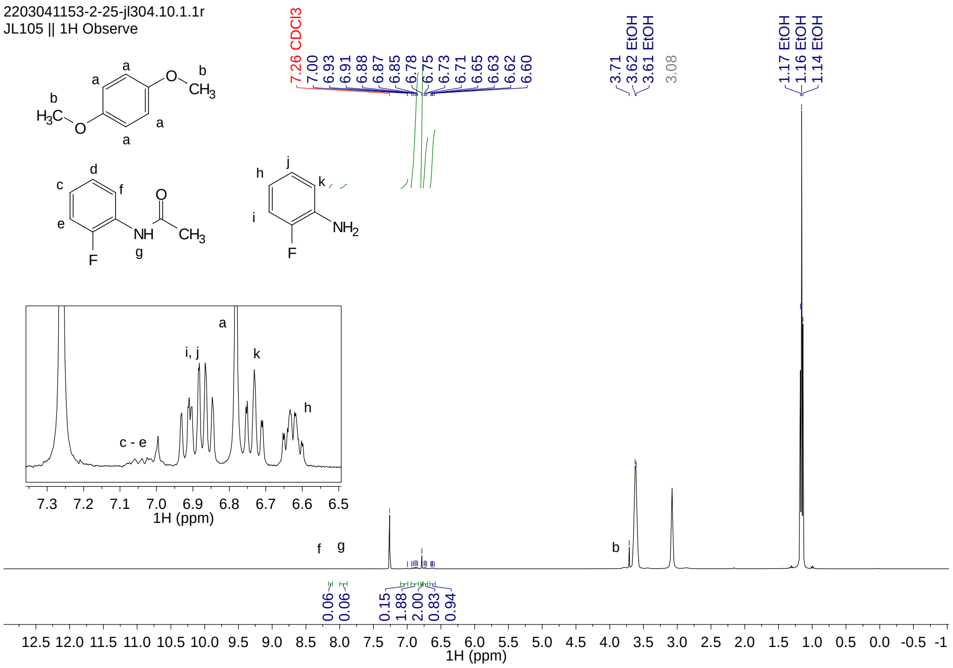

Figure S36: ^{1}H NMR spectrum ($CDCl_3$, 400 MHz, 298 K) of the reaction mixture of hydrogenation of 2’-fluoroacetanilide with the internal standard (Table S2, entry 11).

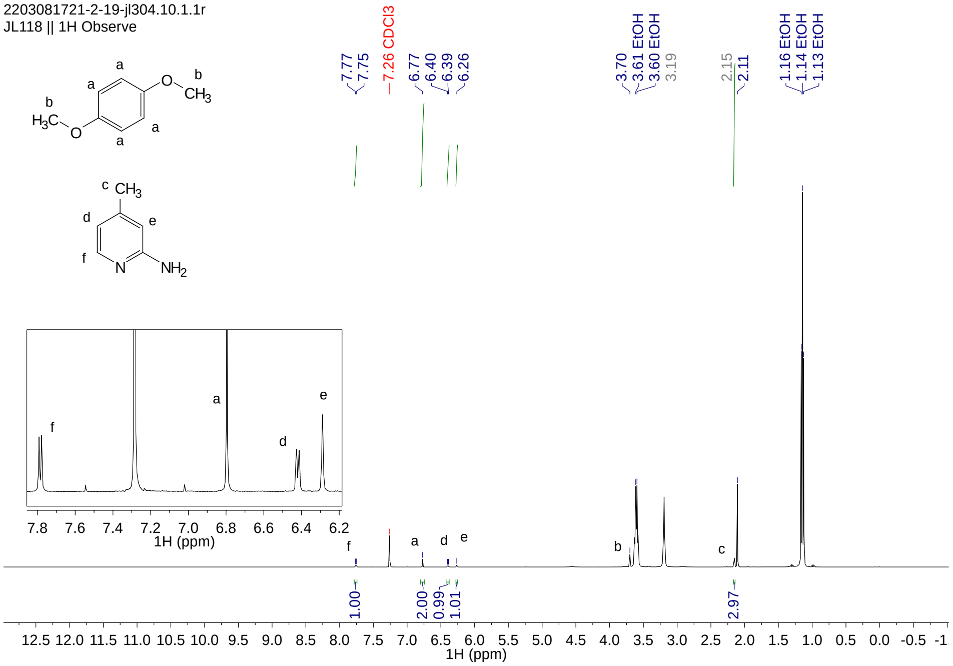

Figure S37: ^{1}H NMR spectrum ($CDCl_3$, 400 MHz, 298 K) of the reaction mixture of hydrogenation of N-(4-methylpyridin-2-yl)acetamide with the internal standard (Table S2, entry 12).

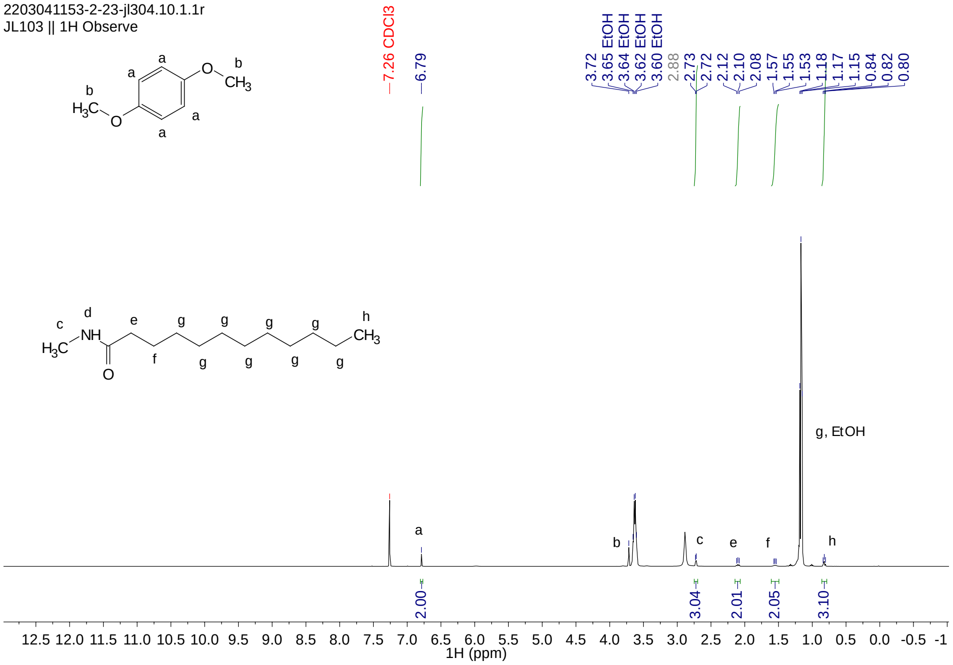

Figure S38: ^{1}H NMR spectrum ($CDCl_3$, 400 MHz, 298 K) of the reaction mixture of hydrogenation of N-methyldodecanamide with the internal standard (Table S2, entry 13).

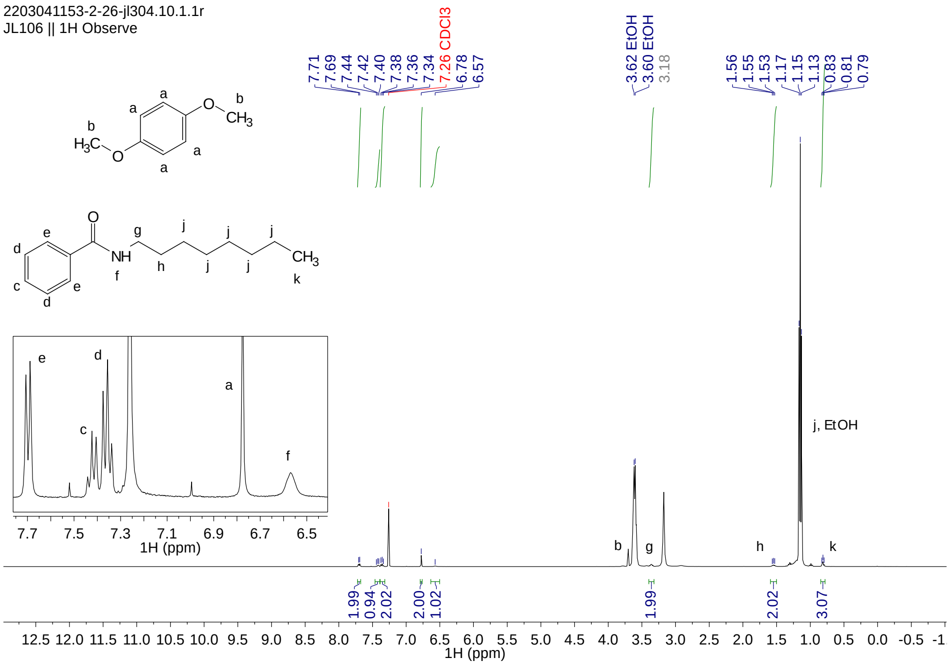

Figure S39: ^{1}H NMR spectrum ($CDCl_3$, 400 MHz, 298 K) of the reaction mixture of hydrogenation of N-octylbenzamide with the internal standard (Table S2, entry 14).

6.4.4. NMR data for the isolated amines from the hydrogenation of amides

^{1}H NMR of 4-methoxyaniline

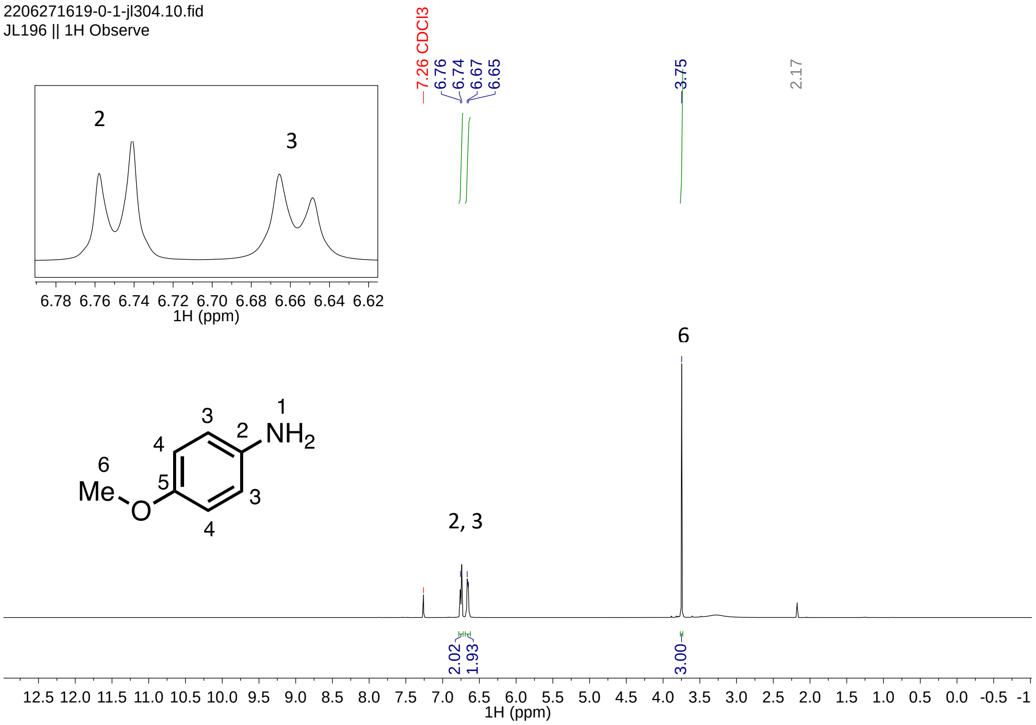

Figure S40: ^{1}H NMR spectrum ($CDCl_3$, 400 MHz, 298 K) of 4-methoxyaniline.

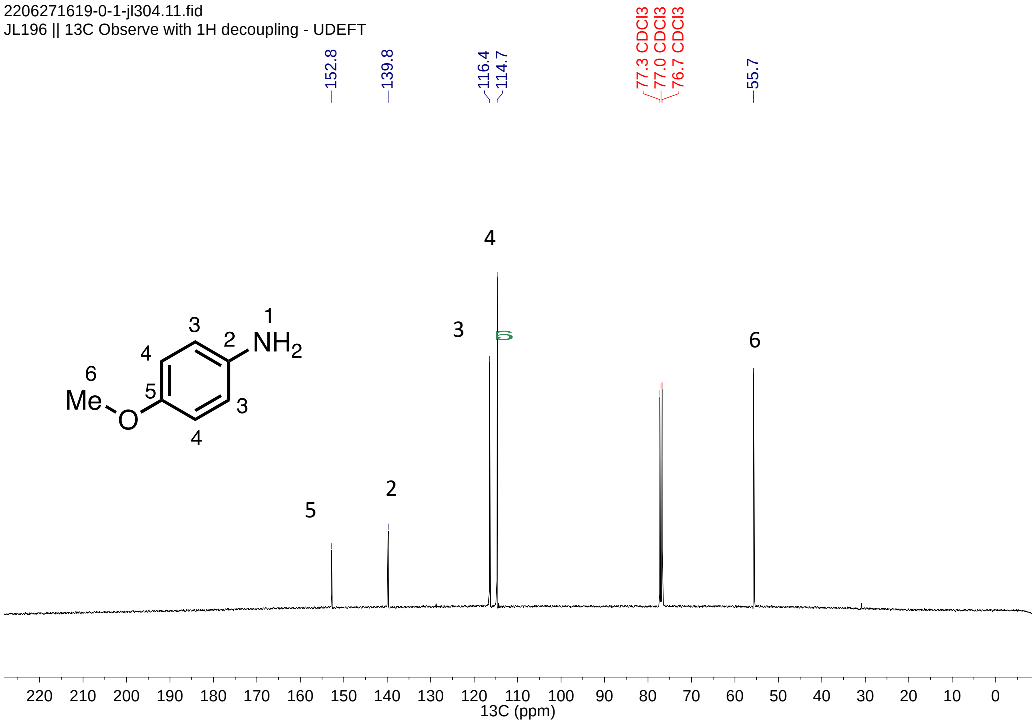

Figure S41: $^{13}C\{^1H\}$ NMR spectrum ($CDCl_3$, 100 MHz, 298 K) of 4-methoxyaniline.

NMR data for 4-bromo-3-chloroaniline

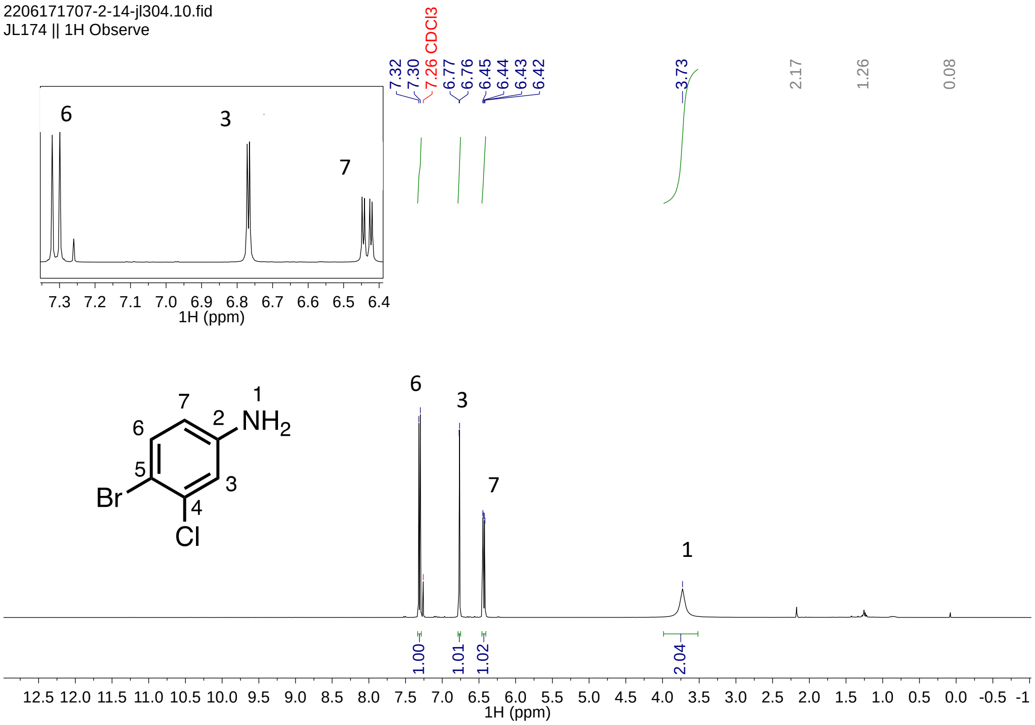

Figure S42: ^{1}H NMR spectrum ($CDCl_3$, 400 MHz, 298 K) of 4-bromo-3-chloroaniline.

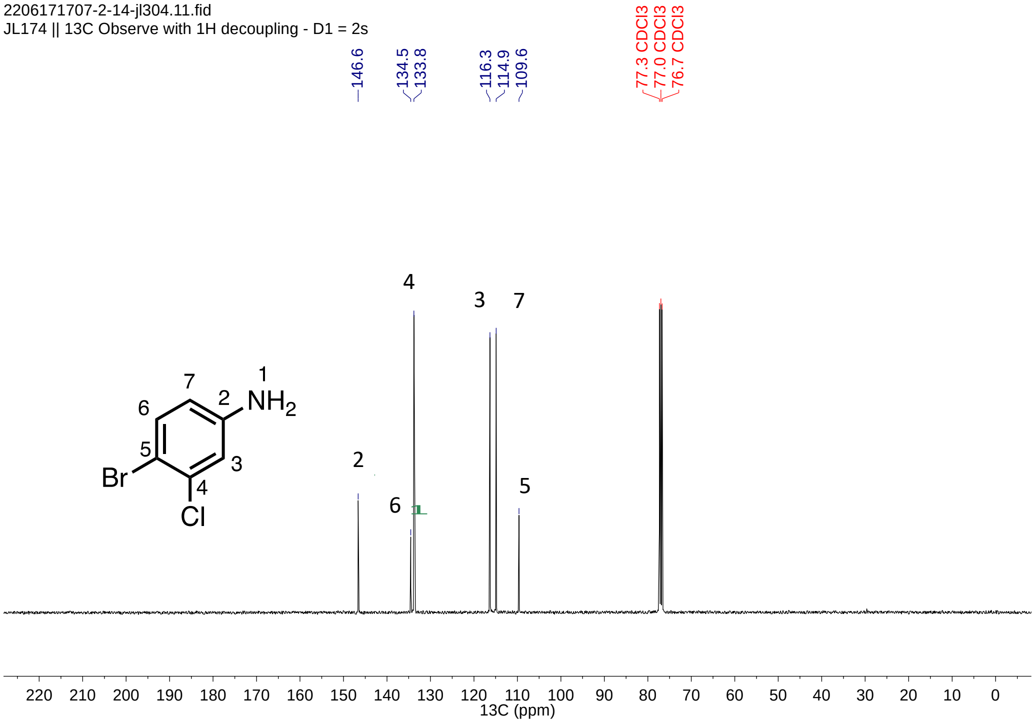

Figure S43: $^{13}C\{^{1}H\}$ NMR spectrum ($CDCl_3$, 100 MHz, 298 K) of 4-bromo-3-chloroaniline.

NMR data for 2-amino-4-methylpyridine

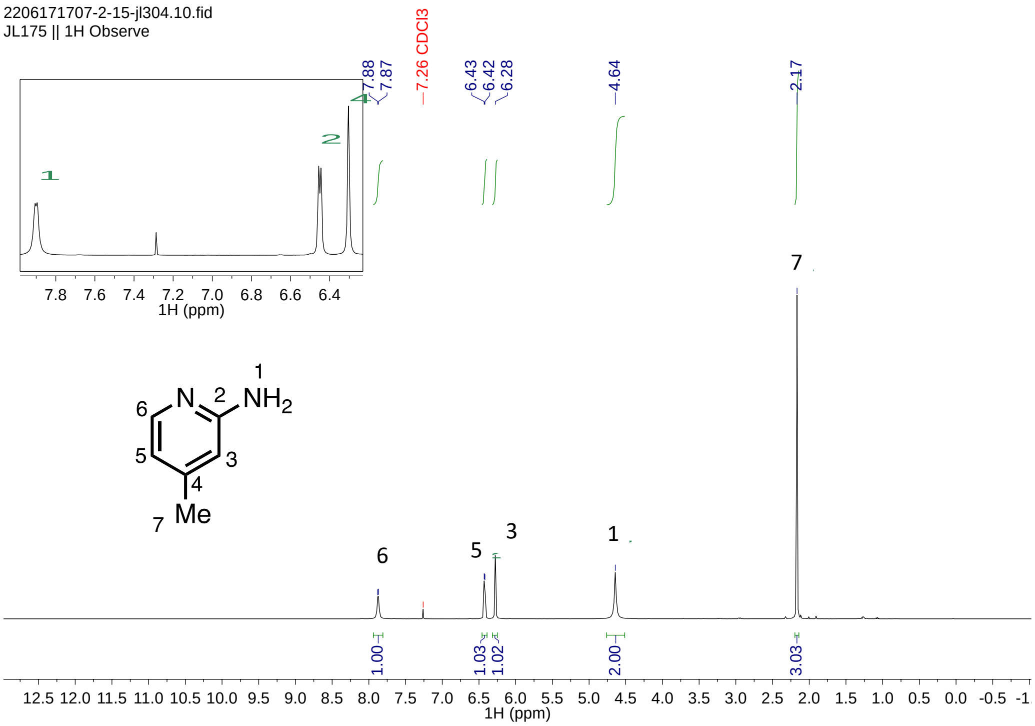

Figure S44: ^{1}H NMR spectrum ($CDCl_3$, 400 MHz, 298 K) of 2-amino-4-methylpyridine.

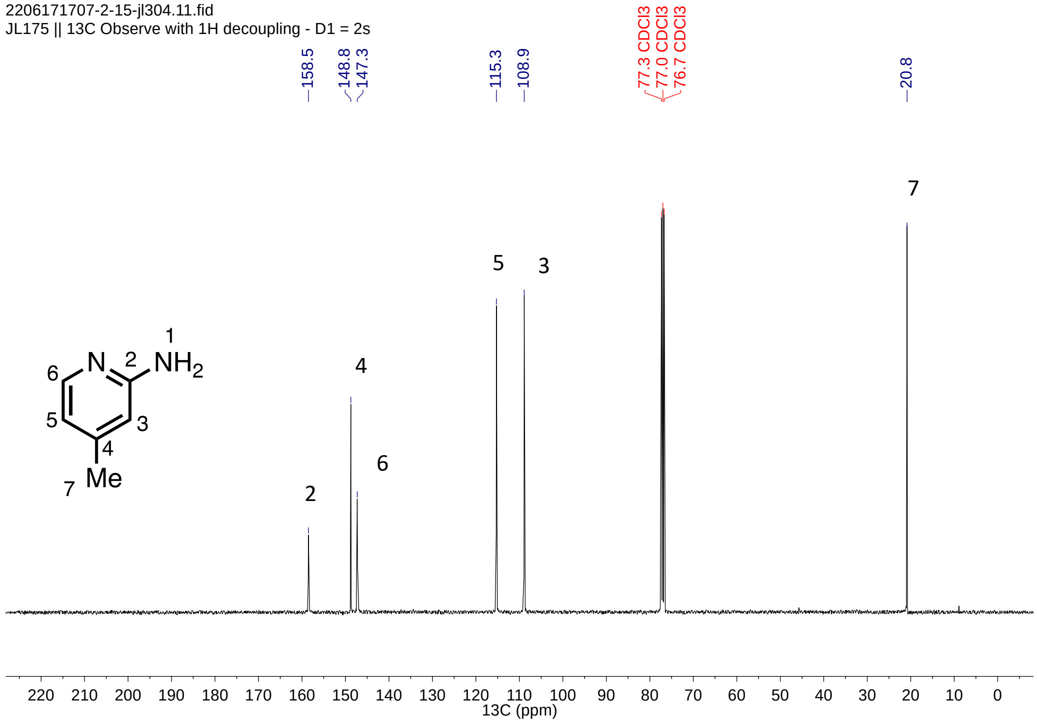

Figure S45: $^{13}C\{^{1}H\}$ NMR spectrum ($CDCl_3$, 100 MHz, 298 K) of 2-amino-4-methylpyridine.

NMR data for aniline

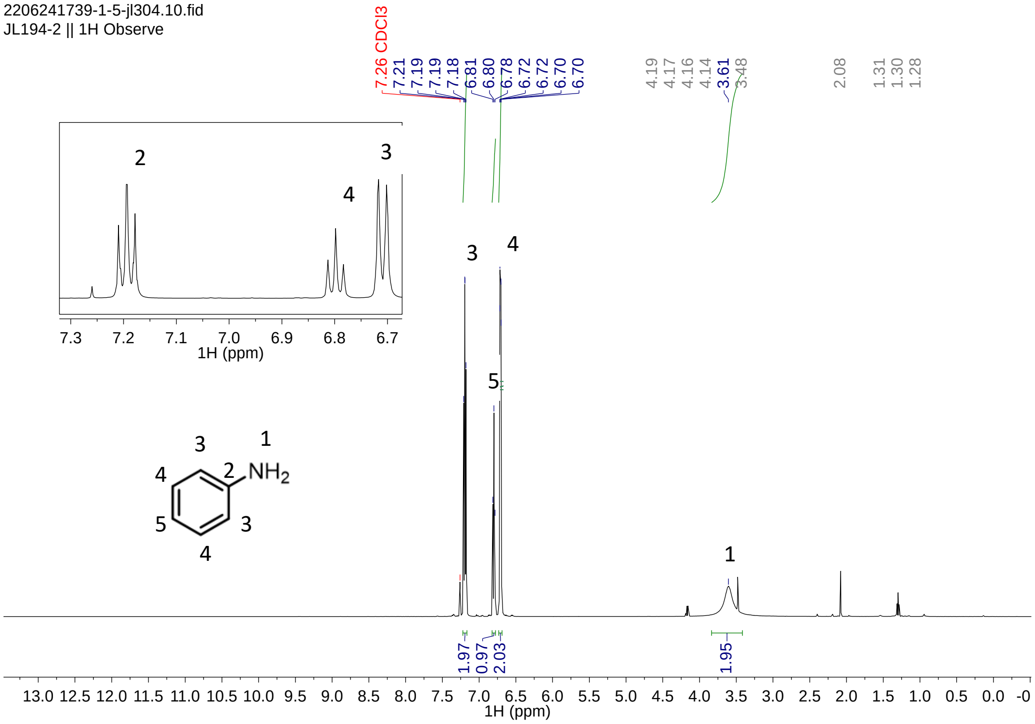

Figure S46: ^{1}H NMR spectrum ($CDCl_3$, 400 MHz, 298 K) of aniline.

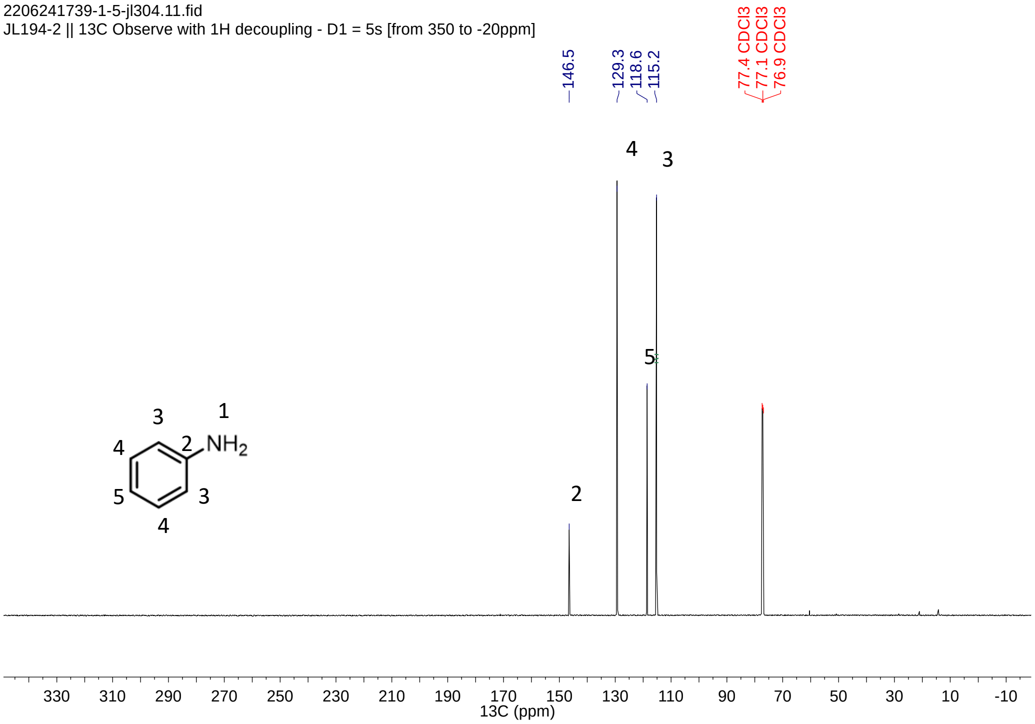

Figure S47: $^{13}C\{^{1}H\}$ NMR spectrum ($CDCl_3$, 100 MHz, 298 K) of aniline.

7. The kinetic resolution of N-aryl amides

7.1. General procedures for the kinetic resolution of N-aryl amides

Preparation of 2'-methoxy-[1,1'-binaphthalen]-2-ol [5]

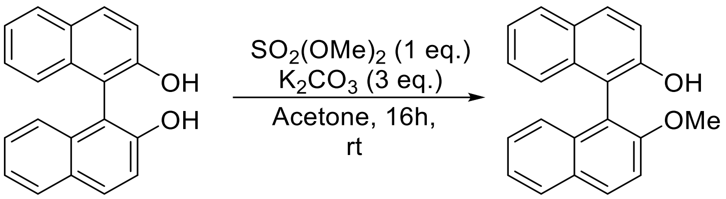

A 100 mL 2-neck flask equipped with rubber septa and charged with a magnetic stirrer was cycled between vacuum and argon atmosphere three times. A rubber septum was briefly removed to allow for the addition of Binol (1.00 g, 3.49 mmol, 1 eq.) and K_2CO_3 (1.45 g, 10.47 mmol, 3 eq.) and the flask was placed back under vacuum for 10 minutes. After cycling between vacuum and argon atmosphere three times, acetone (18 mL) was added *via* syringe followed by dimethyl sulfate (0.33 mL, 3.49 mmol, 1 eq.) also added *via* syringe. The reaction was stirred for 16 h at room temperature and the reaction mixture was filtered through celite, concentrated *in vacuuo* and purified by flash column chromatography on silica gel using CH_2Cl_2 as eluent.

Yield (3.49 mmol scale): 696 mg (67%), off-white solid.
^{1}H NMR (500 MHz, $CDCl_3$) δ 8.06 (1H, d, *J* = 9.2 Hz, Ar-H), 7.92 – 7.89 (2H, m, 2Ar-H), 7.86 (1H, d, *J* = 8.2 Hz, Ar-H), 7.49 (1H, d, *J* = 9.2 Hz, Ar-H), 7.40 – 7.34 (2H, m, 2Ar-H), 7.33 – 7.27 (2H, m, 2Ar-H), 7.24 – 7.20 (1H, m, Ar-H), 7.19 – 7.16 (1H, m, Ar-H), 7.07 – 7.03 (1H, m, Ar-H), 3.81 (3H,s, OMe). ^{13}C{^{1}H} NMR (126 MHz, $CDCl_3$) δ 156.0 (Ar-O), 151.2 (Ar-O), 134.0 (Ar-C), 133.8 (Ar-C), 131.1 (Ar-H), 129.8 (Ar-H), 129.4 (Ar-C), 129.1 (Ar-C), 128.2 (Ar-H), 128.2 (Ar-H), 127.4 (Ar-H), 126.4 (Ar-H), 124.9 (Ar-H), 124.8 (Ar-H), 124.2 (Ar-H), 123.3 (Ar-H), 117.5 (Ar-H), 115.2 (Ar-C), 115.0 (Ar-C), 113.8 (Ar-H), 56.7 (CH_3). The compound has previously been produced by the same method and the data reported here is consistent with the previously reported data.[5]
The reaction was conducted multiple times giving product in 51-67% yield.

Preparation of 2-bromopropanamide[6]

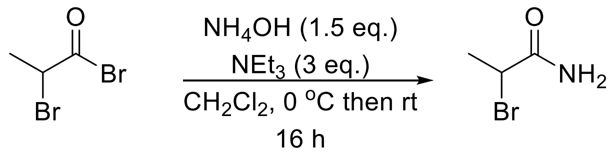

To a 100 mL round bottom flask charged with a magnetic stirrer was added NH_4OH (35% v/v in H_2O; 5.1 mL, 45 mmol, 1.5 eq.) and NEt_3 (12.5 mL, 90 mmol, 3 eq.). The reagents were dissolved in CH_2Cl_2 (60 mL) and the reaction was cooled to 0 °C. 2-bromopropanoyl bromide (3.14 mL, 30 mmol, 1 eq.) was then added dropwise and the reaction was stirred overnight, allowing the reaction to gradually reach rt. Upon this time, the reaction was washed with brine (3 x 20 mL) and concentrated *in vacuuo* to give the product without further purification.

Yield (30 mmol scale): 2.11 g (46%), brown solid.

^{1}H NMR (500 MHz, $CDCl_3$) δ 6.30 (1H, s, N**H**H'), 5.62 (1H, s, NH**H'**), 4.41 (q, *J* = 7.1 Hz, 1H), 1.90 (d, *J* = 7.0 Hz, 3H). ^{13}C NMR (126 MHz, $CDCl_3$) δ 172.1 (C=O), 44.0 (CH), 23.1 (CH_3). The compound has been prepared before and the data reported here is consistent with the previously reported data.[6]

Preparation of 2'-methoxy-[1,1'-binaphthalen]-2-aminium chloride[6]

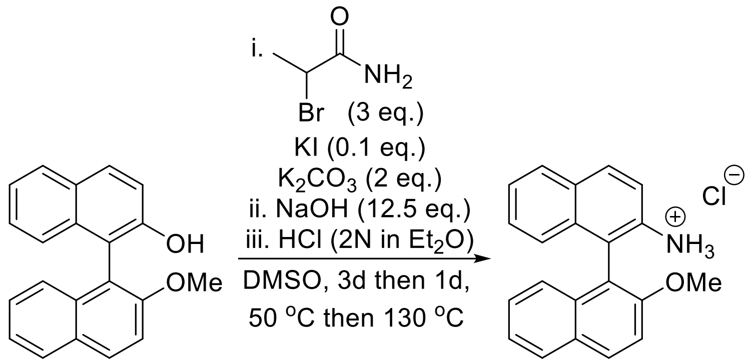

To a 50 mL screw cap vial charged with magnetic stirrer was added 2'-methoxy-[1,1'-binaphthalen]-2-ol (1.20 g, 4 mmol, 1 eq.), 2-bromopropanamide (1.52 g, 10 mmol, 2.5 eq.), KI (66.4 mg, 0.4 mmol, 0.1 eq.) and K_2CO_3 (1.38 g, 10 mmol, 2.5 eq.). The vial was capped and the reaction stirred vigorously at 50 °C for 72 h. The reaction was then allowed to cool to rt before NaOH (2.0 g, 50 mmol, 12.5 eq.) was added. The reaction was then heated to 130 °C and stirred for 24 h. The reaction was then quenched with water (50 mL) and extracted with CH_2Cl_2 (3 x 30 mL). The material was concentrated *in vacuuo* before dissolving in Et_2O and adding HCl (2N in Et_2O, 3 mL, 1.5 eq.). The precipitate was collected by filtration and washed extensively with CH_2Cl_2 to yield the product. The compound has been prepared before and the data reported here is consistent with the previously reported data.[6]

Yield (4 mmol scale): 690 mg (51%) of white solid.

^{1}H NMR (500 MHz, MeOD) δ 8.22 (1H, d, *J* = 9.2 Hz, Ar-H), 8.19 (1H, d, *J* = 8.8 Hz, Ar-H), 8.06 (1H, d, *J* = 8.3 Hz, Ar-H), 8.00 (1H, d, *J* = 8.2 Hz, Ar-H), 7.67 (1H, d, *J* = 9.2 Hz, Ar-H), 7.63 (1H, d, *J* = 8.8 Hz, Ar-H), 7.60 – 7.55 (1H, m, Ar-H), 7.41 – 7.33 (2H, m, 2Ar-H), 7.31 – 7.26 (1H, m, Ar-H), 7.08 (1H, d, *J* = 8.5 Hz, Ar-H), 6.89 (1H, d, *J* = 8.5 Hz, Ar-H), 3.86 (3H, s, OMe). ^{13}C NMR (126 MHz, MeOD) δ 156.6 (Ar-O), 135.0 (Ar-C), 134.7 (Ar-C), 134.4 (Ar-C), 133.2 (Ar-H), 131.4 (Ar-H), 130.7 (Ar-N), 129.8 (Ar-C), 129.6 (Ar-H), 129.5 (Ar-H), 128.7 (Ar-H), 128.6 (Ar-H), 128.2 (Ar-H), 127.4 (Ar-H), 125.2 (Ar-H), 125.1 (Ar-H), 121.3 (Ar-H), 115.4 (Ar-H), 114.5 (Ar-H), 56.8 (CH_3). HRMS (ESI+): Calculated for $[C_{21}H_{18}NO]$: 300.1383 Found: 300.1380.

Preparation of *N*-(2'-methoxy-[1,1'-binaphthalen]-2-yl)benzamide

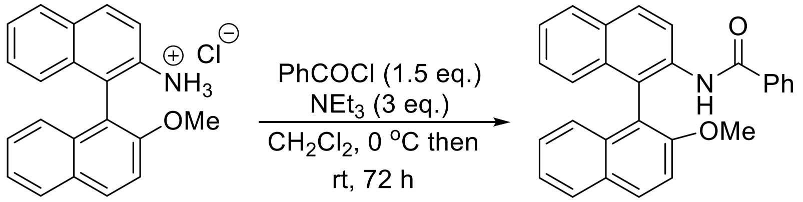

2'-methoxy-[1,1'-binaphthalen]-2-aminium chloride (808 mg, 2.7 mmol, 1 eq.) was suspended in CH_2Cl_2 (9 mL) and NEt_3 (1.13 mL, 8.1 mmol, 3 eq.) was added *via* syringe, dissolving the amine. The solution was cooled to 0 °C and PhCOCl (0.47 mL, 4.05 mmol, 1.5 eq.) was added dropwise *via* syringe. The reaction was stirred for 72 h and quenched with water (25 mL), washed with HCl (1M, 50 mL) and extracted with CH_2Cl_2 (3 x 20 mL). The combined organic phases were washed with conc. $NaHCO_3$ (50 mL), then brine (50 mL) and then dried over $MgSO_4$. The solution was filtered and then concentrated *in vacuuo*. The crude solid was recrystallised from hexane/CH_2Cl_2, the crystals were washed with hexane and MeOH and dried by a high vacuum line to give product.

Yield (2.7 mmol scale): 832 mg (76%) of pale yellow crystals.
^{1}H NMR (500 MHz, Chloroform-*d*) δ 8.87 – 8.80 (1H, m, Ar-H), 8.10 (d, *J* = 9.0 Hz, Ar-H), 8.05 (1H, d, *J* = 9.0 Hz, Ar-H), 7.93 (d, *J* = 8.3 Hz, 1H), 7.77 (1H, bs, N-H), 7.52 (1H, d, *J* = 9.0 Hz, Ar-H), 7.43 – 7.35 (3H, m, 3Ar-H), 7.30 – 7.27 (1H, m, Ar-H), 7.26 – 7.25 (1H, m, Ar-H), 7.25 – 7.22 (4H, m, 4Ar-H), 7.17 – 7.13 (2H, m, 2Ar-H), 3.78 (3H, s, OMe). ^{13}C NMR (126 MHz, $CDCl_3$) δ 165.2 (C=O), 155.3 (Ar-O), 135.1 (Ar-C), 134.7 (Ar-C), 133.6 (Ar-C), 133.1 (Ar-C), 131.6 (Ar-H), 131.2 (Ar-H), 131.1 (Ar- C), 129.4 (Ar-C), 129.0 (Ar-N), 128.7 (Ar-H), 128.3 (Ar-H), 128.3 (Ar-H), 127.7 (Ar-H), 126.8 (Ar-H), 126.5 (Ar-H), 125.9 (Ar-H), 125.0 (Ar-H), 124.9 (Ar-H), 124.5 (Ar-H), 121.6 (Ar-C), 120.4 (Ar-C), 117.1 (Ar-H), 113.7 (Ar-H), 56.8 (CH_3). HRMS (ESI+): Calculated for [$C_{28}H_{21}NO$]: 404.1645 Found: 404.1643. Compound has been prepared previously by an alternative route and the data reported here is consistent with the previously reported data.[7]

General procedure for the Kinetic Resolution of *N*-(2'-methoxy-[1,1'-binaphthalen]-2-yl)benzamide

A microwave vial charged with a magnetic stirrer bar and fitted with rubber septum was flame dried under vacuum. The vial was cooled to room temperature and then cycled between vacuum and Ar atmosphere three times. Working quickly, the rubber septum was removed then: *N*-(2'-methoxy-[1,1'-binaphthalen]-2-yl)benzamide (1 eq.), catalyst (1 mol%) and base (0.1 eq.) were added and the vial fitted with a crimp camp and septum. The vial was placed back under vacuum for a further 10 minutes. After cycling the vacuum and argon atmosphere three more times, solvent (0.1 M) was added via syringe, the septum was pierced by two 21G needles and the vial was placed directly into an autoclave which had been cycled between the vacuum and Ar atmosphere 3 times. The autoclave was sealed and pressurised to 15 Bar of H_2 which was subsequently vented, this was repeated two more times to degas the solvent. Finally, the autoclave was pressurised to 50 Bar H_2 and the autoclave was then placed in an oil bath preheated to 50 °C and stirred at 700 rpm for 18 h. Upon this time, the autoclave was cooled to rt, carefully vented in a fume cupboard and 1,4-dimethoxybenzene (0.25 eq. [relative to substrate]) was added to the sample as internal standard. The reaction was diluted in CDCl3 (1 mL) and a small aliquot was taken for ^{1}H NMR analysis to determine conversion. The reactions were then washed with brine (25 mL), extracted with CH_2Cl_2 (3 x 10 mL) and dried over Na_2SO_4. The solution was filtered and concentrated *in vacuum* prior to HPLC analysis.

Chiral analysis was performed using a Chiralcel AD-H column using hexane/^{i}PrOH (70:30) as mobile phase with a 0.5 mL/min flow rate.

7.2. NMR spectra for kinetic resolution of N-aryl amides

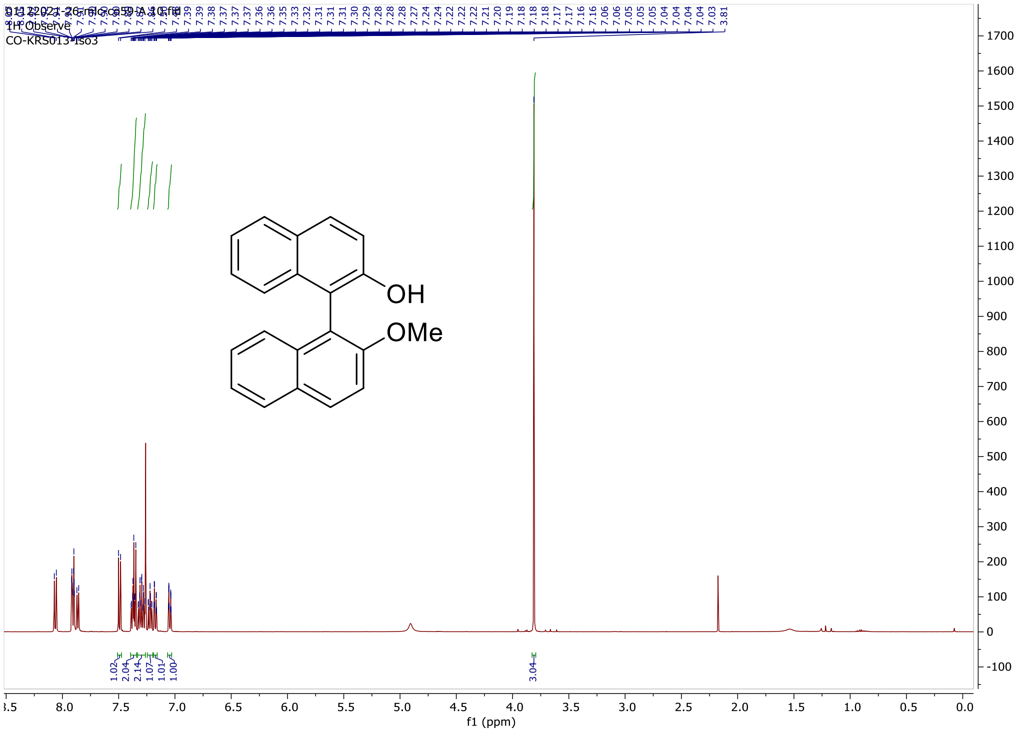

Figure S48: ^{1}H NMR spectrum ($CDCl_3$, 500 MHz, 298 K) of 2'-methoxy-[1,1'-binaphthalen]-2-ol.

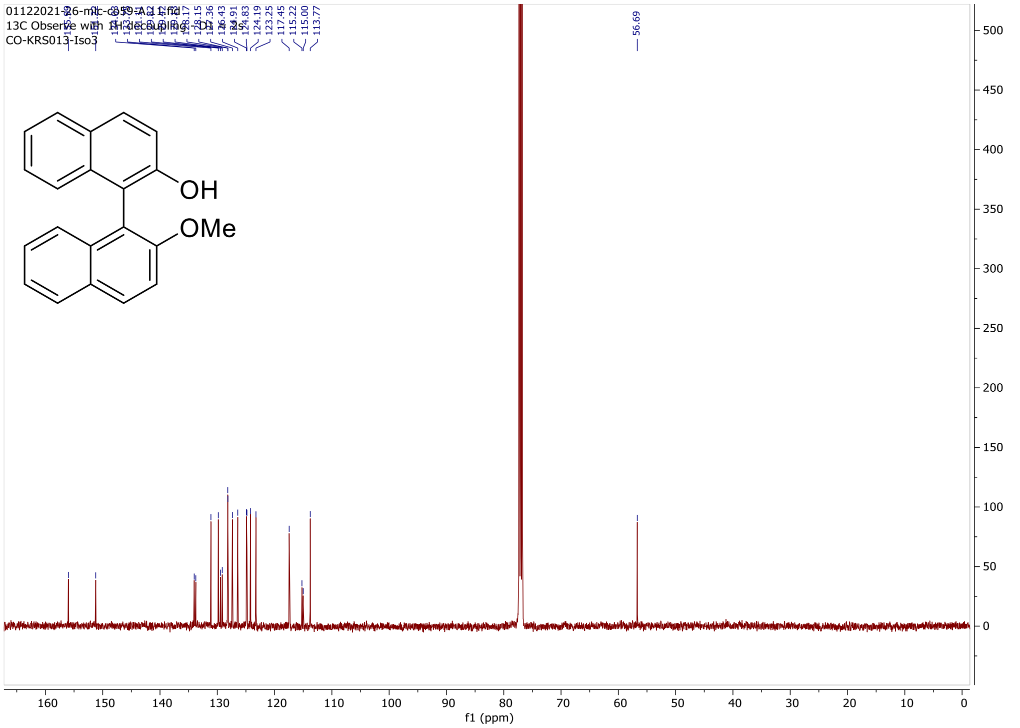

Figure S49: ^{13}C NMR spectrum ($CDCl_3$, 126 MHz, 298 K) of 2'-methoxy-[1,1'-binaphthalen]-2-ol.

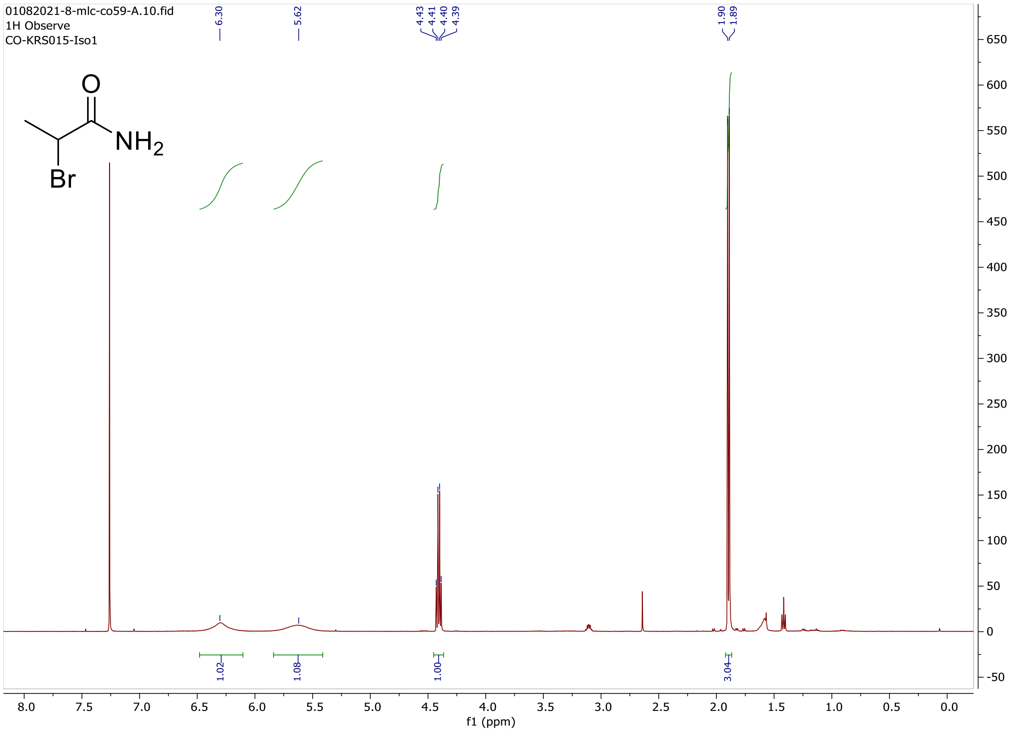

Figure S50: ^{1}H NMR spectrum ($CDCl_3$, 500 MHz, 298 K) of 2-bromopropanamide.

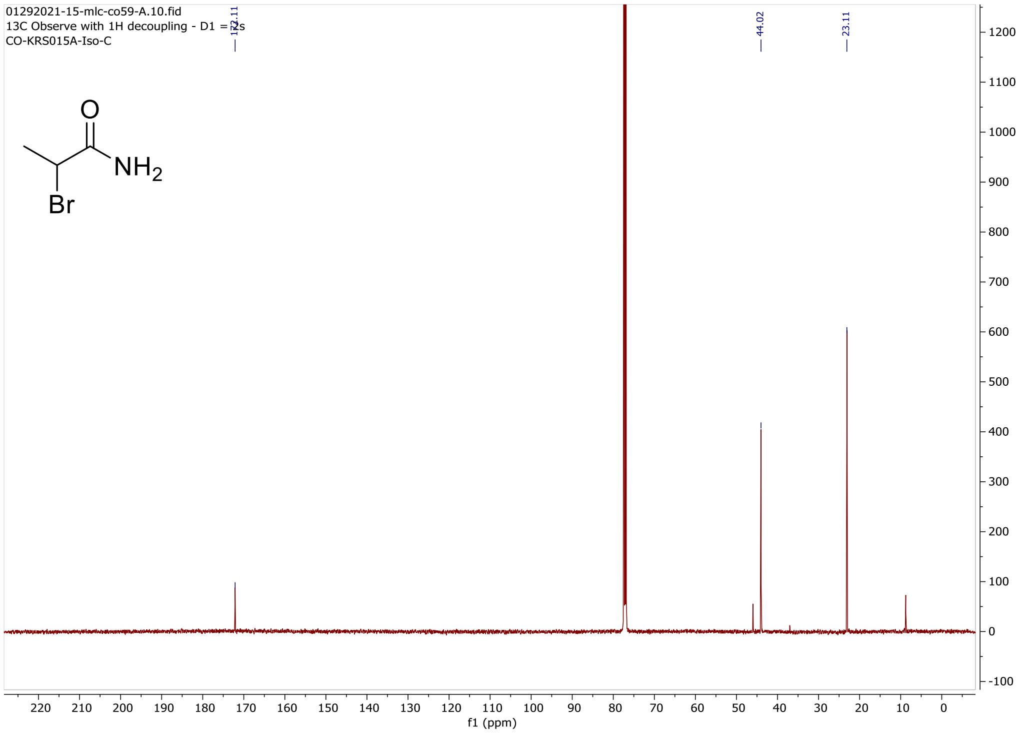

Figure S51: ^{13}C NMR spectrum ($CDCl_3$, 126 MHz, 298 K) of 2-bromopropanamide.

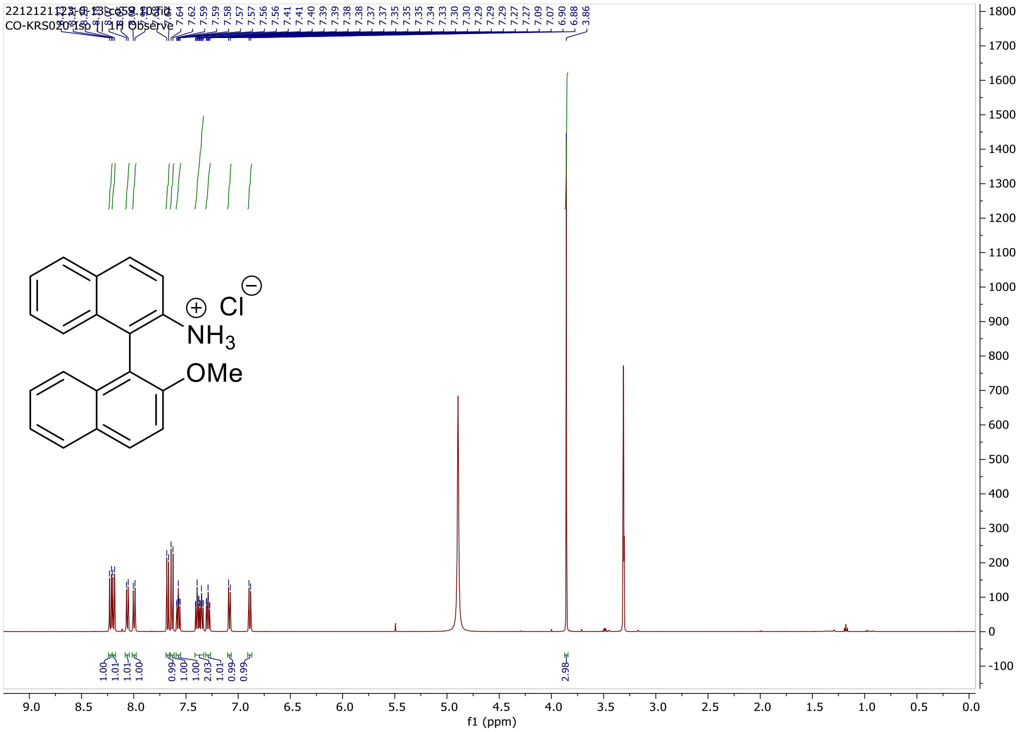

Figure S52: ^{1}H NMR spectrum (MeOD, 500 MHz, 298 K) of 2'-methoxy-[1,1'-binaphthalen]-2-aminium chloride.

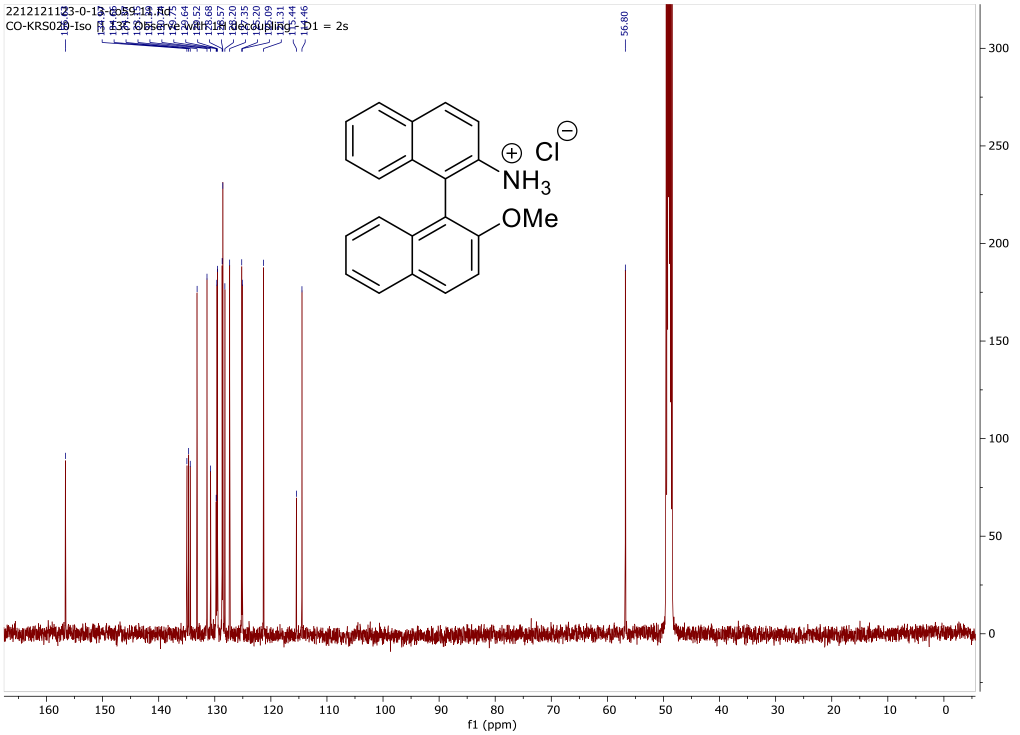

Figure S53: ^{13}C NMR spectrum (MeOD, 126 MHz, 298 K) of 2'-methoxy-[1,1'-binaphthalen]-2-aminium chloride.

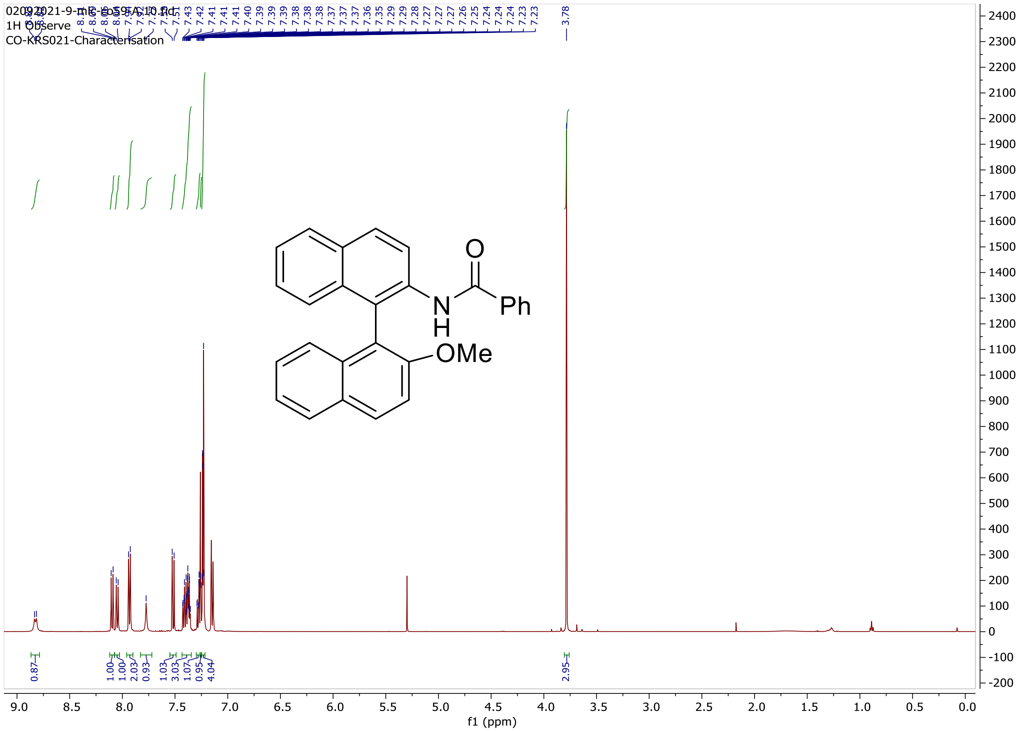

Figure S54: ^{1}H NMR spectrum ($CDCl_3$, 500 MHz, 298 K) of *N*-(2'-methoxy-[1,1'-binaphthalen]-2-yl)benzamide.

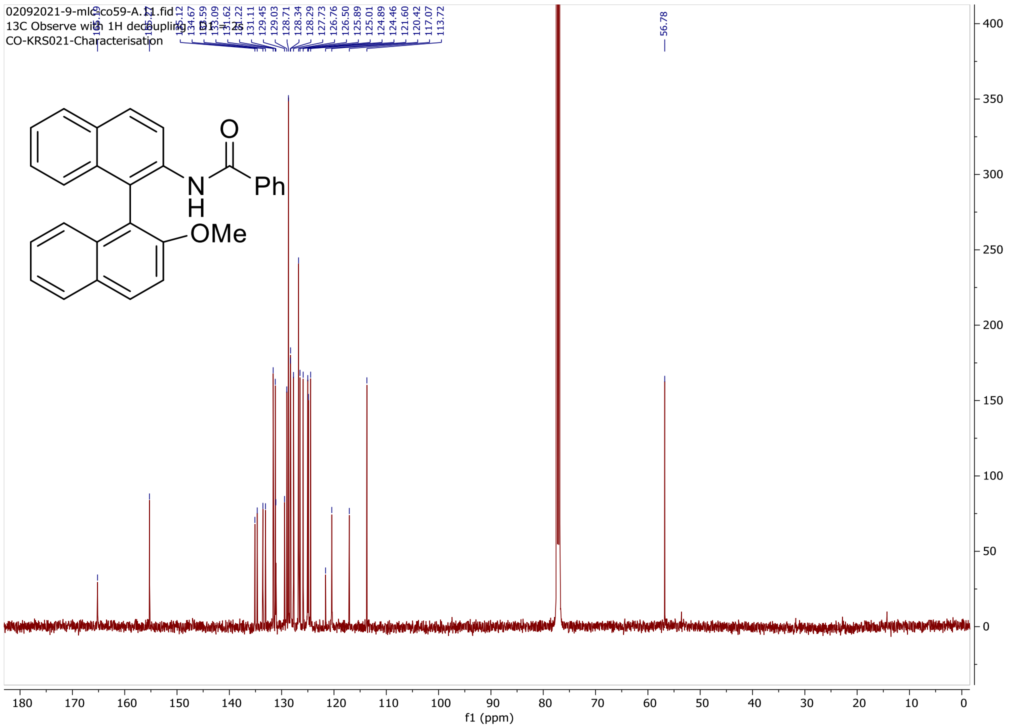

Figure S55: ^{13}C NMR spectrum ($CDCl_3$, 126 MHz, 298 K) of *N*-(2'-methoxy-[1,1'-binaphthalen]-2-yl)benzamide.

7.3. Hydrogenolysis of N-aryl amide with kinetic resolution using manganese complex 1

Table S7. Hydrogenolysis of N-aryl amide with kinetic resolution using manganese complex **1**

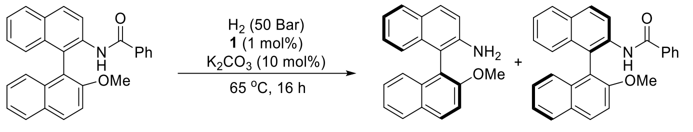

Entry	Solvent	Conc. (M)	Temp (°C)	Conversion	E.R. of A
1.	EtOH	1	65	3%	
2.	EtOH	0.5	65	7%	
3.	EtOH:Tol (90:10)	0.25	65	18%	
4.	EtOH:Tol (80:20)	0.25	65	12%	
5.	EtOH:2-MeTHF (90:10)	0.25	65	15%	
6.	EtOH:2-MeTHF (80:20)	0.25	65	24%	
7.	EtOH:2-MeTHF (50:50)	0.25	65	22%	
8.	EtOH:2-MeTHF (50:50)	0.1	65	70%	58(S-):42 (R-)
9.	EtOH:2-MeTHF (50:50)	0.1	55	20%	62 (S-): 38 (R-)
10.	EtOH:2-MeTHF (80:20)	0.1	65	17%	56 (S-): 44 (R-)
11.[b]	2-MeTHF	0.1	65	25%	64 (S-): 36 (R-)
12.[c]	2-MeTHF	0.1	65	15%	64 (S-): 36 (R-)

8. Hydrogenation of polyurethanes

8.1. General procedures for hydrogenation of polyurethanes

General procedures for synthesis of model polyurethanes

The desired diisocyanate (10 mmol) was added to a solution of the desired diol (10 mmol) in DMF (10 mL). The reaction was heated to 80 °C and stirred for 1.5 h. The precipitate was collected by filtration and washed with methanol, before being dried *in vacuo* to afford the desired product as a white solid.[8]

General procedure for hydrogenation of polyurethanes

Polymer substrate (0.25 mmol, 0.25 equivalent, weight calculated relative to the molecular weight of the monomer), potassium carbonate (6.9 mg, 0.05 mmol, 20 mol%) and Complex **1** (4.2 mg, 0.005 mmol, 2 mol%) were added to a flame-dried microwave vial before the vial was evacuated and purged with argon. THF (1 mL) was then added to the vial before the vial was pierced with two needles and added to an autoclave, which had already been purged with argon. The autoclave was charged with 50 bar of H_2 pressure, before being heated at 130 °C and allowed to stir for 16 hours. The mixture was then filtered and the solid was washed with methanol to dissolve the reaction products. Conversions of the substrate to product were then calculated using the integrals of signals attributed to the product relative to those attributed to the reactant, using 1,1-diphenylethylene (22.5 mg, 0.125 mmol) as an internal standard.

8.2. Results from the hydrogenation of polyurethanes

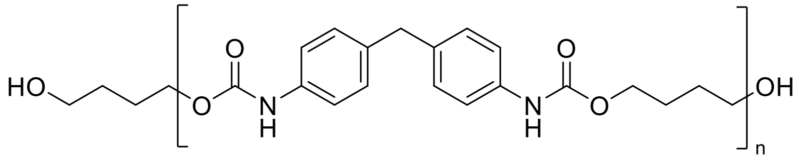

PU-1

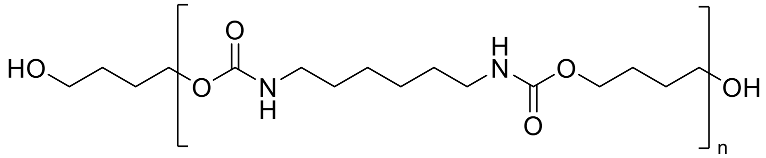

PU-2

Figure S56: Model polyurethane structures

Table S8: Hydrogenation of polyurethanes

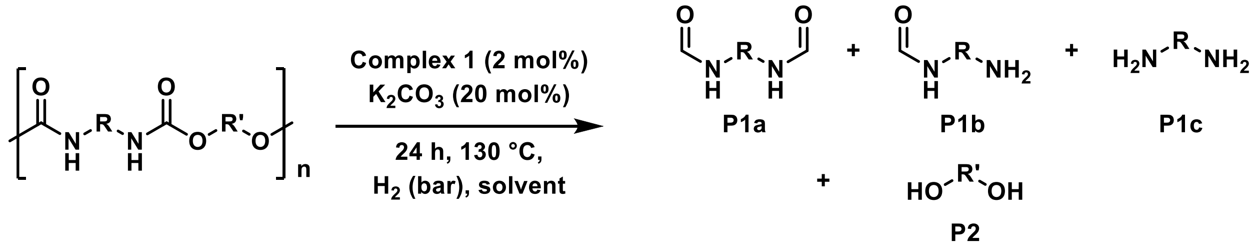

Entry	Polyurethane	Solvent	P1a, P1b, P1c, combined yield (%)	P2 yield[a]
**1**[b]	**PU-1**	EtOH	-	-
**2**[c]	**PU-1**	THF	20	20
**3**[d]	**PU-2**	THF	0	0

[a]yield is calculated by the ^{1}H NMR spectroscopy using 1,1-diphenylethylene as an internal standard.[b]Using EtOH as solvent resulted in a mixture of products hypothesized to be unethylated, monoethylated and diethylated amines so yield was not able to be determined by the ^{1}H NMR spectroscopy. [c]~75% of the polymer/oligomer was recovered by weight. [d]100% of the polymer/oligomer was recovered by weight.

8.3. NMR and IR data of model polyurethanes

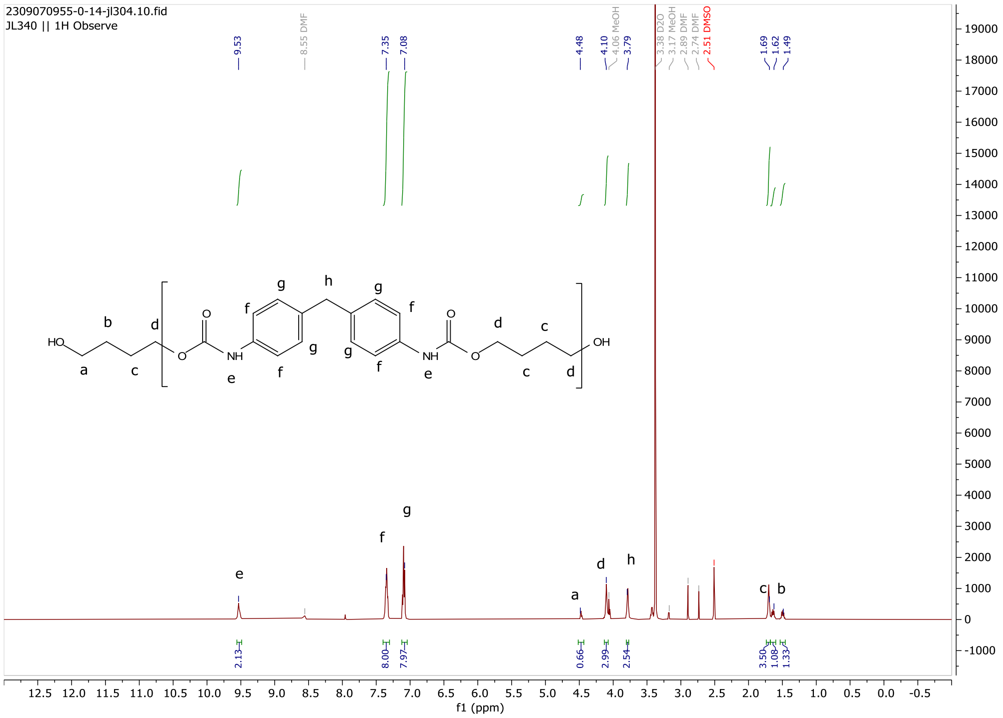

Figure S57: ^{1}H NMR spectrum (DMSO-d_6, 400 MHz, 298 K) of model polyurethane **PU-2**.

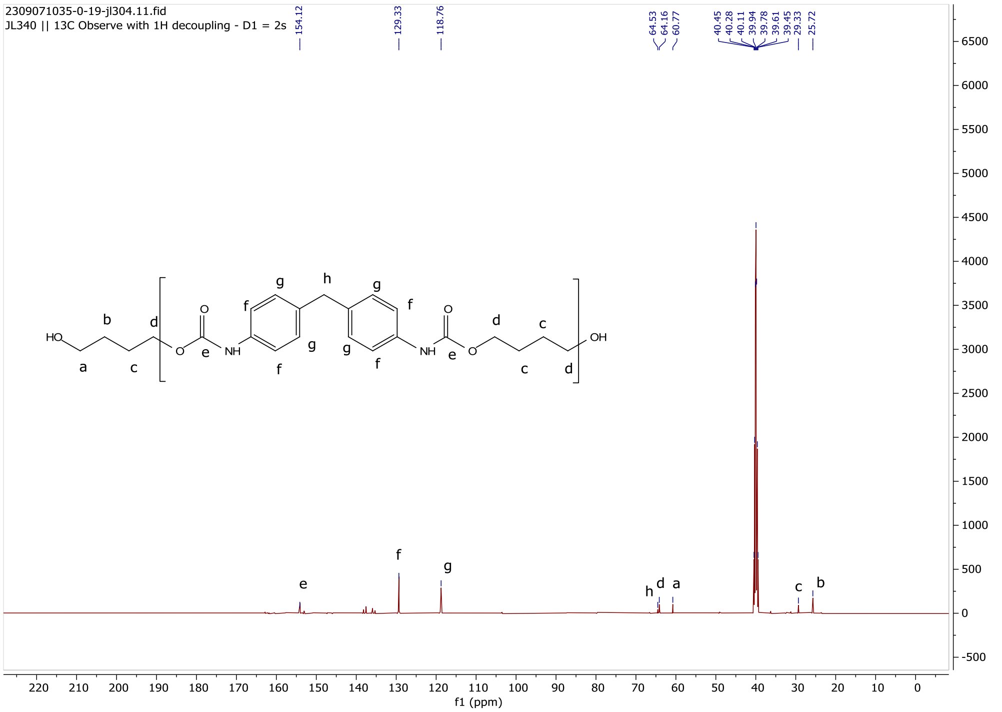

Figure S58: $^{13}C\{^{1}H\}$ NMR spectrum (DMSO-d_6, 100 MHz, 298 K) of model polyurethane **PU-2**.

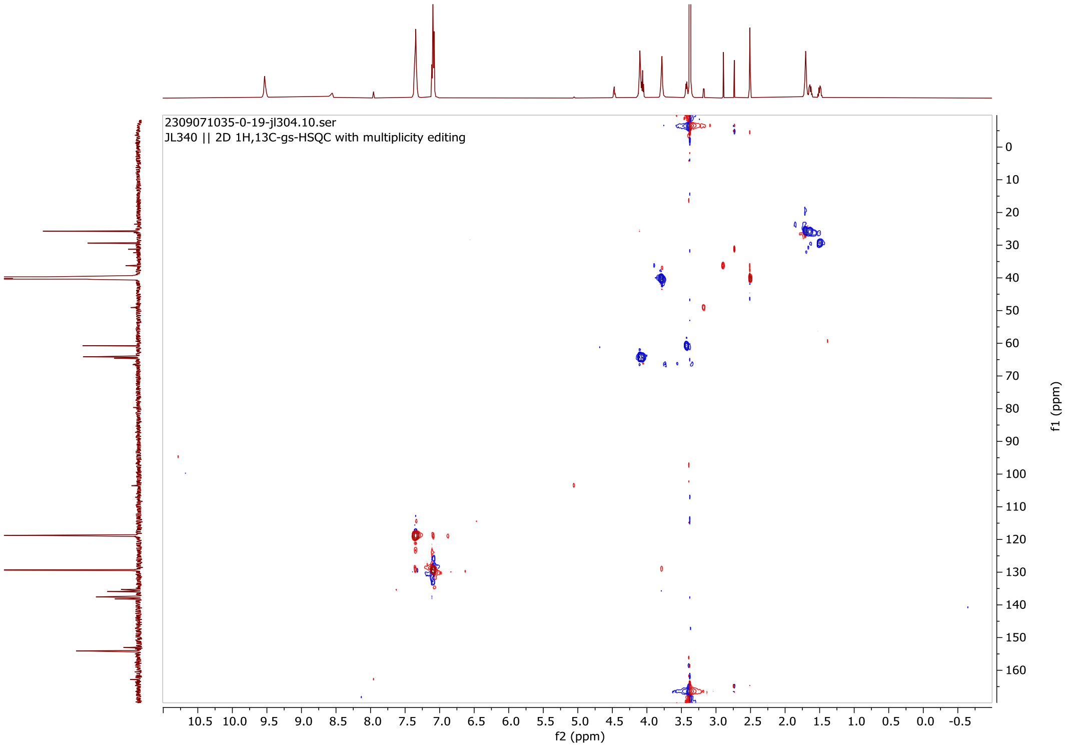

Figure S59: 2D ^{1}H, ^{13}C HSQC spectrum (DMSO-d_6, 400 MHz, 298 K) of model polyurethane **PU-2**.

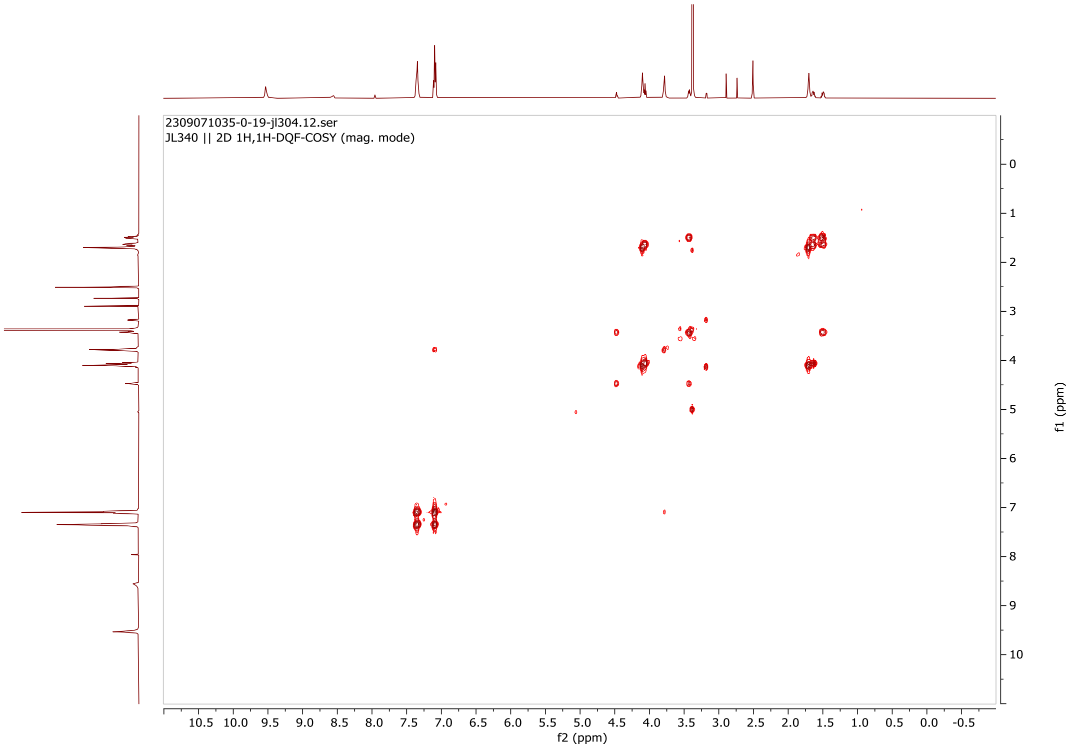

Figure S60: 2D ^{1}H, ^{1}H COSY spectrum (DMSO-d_6, 400 MHz, 298 K) of model polyurethane **PU-2**.

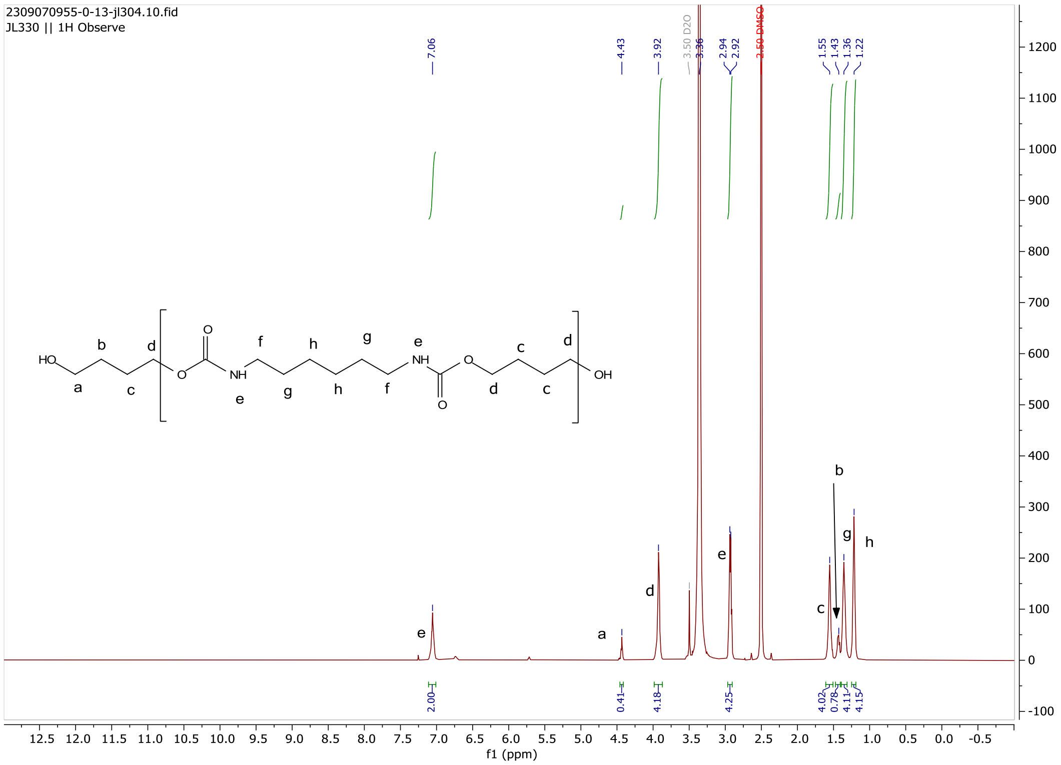

Figure S61: ^{1}H NMR spectrum (DMSO-d_6, 400 MHz, 298 K) of model polyurethane **PU-2**.

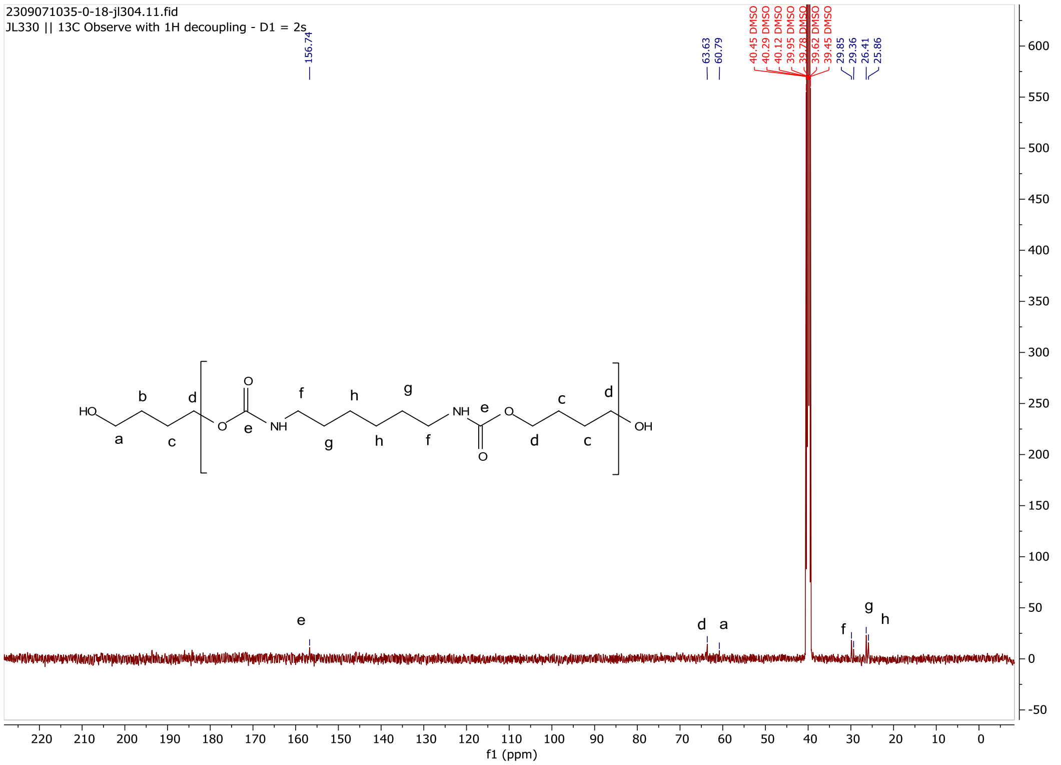

Figure S62: ^{13}C{^{1}H} NMR spectrum (DMSO-d_6, 100 MHz, 298 K) of model polyurethane **PU-2**.

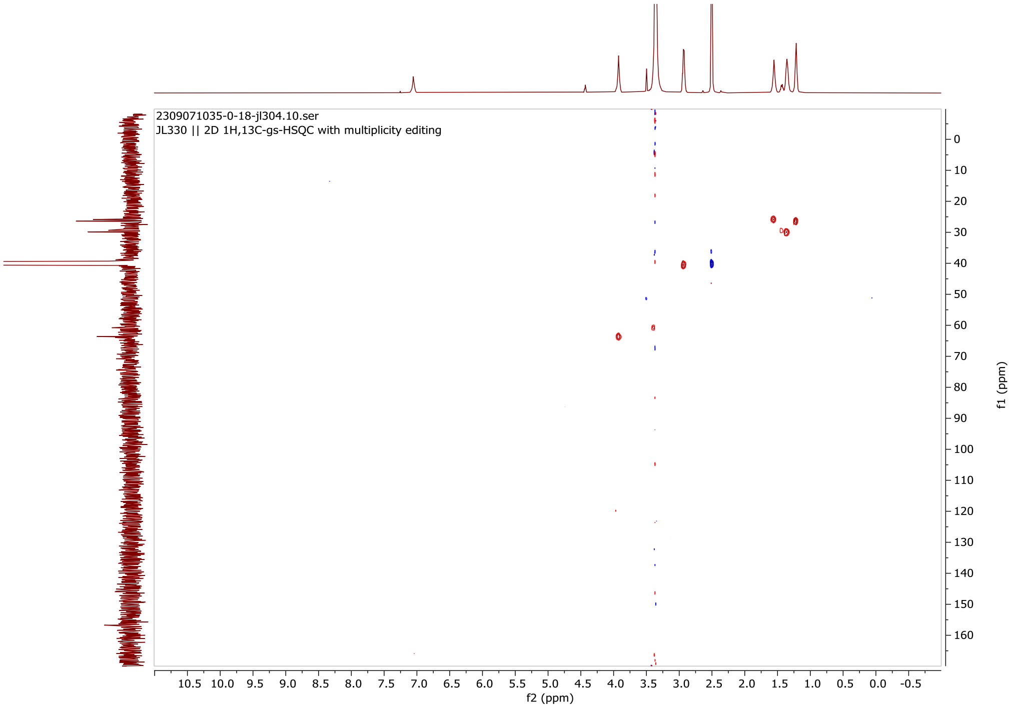

Figure S63: 2D ^{1}H,^{13}C HSQC spectrum (DMSO-d_6, 400 MHz, 298 K) of model polyurethane **PU-2**.

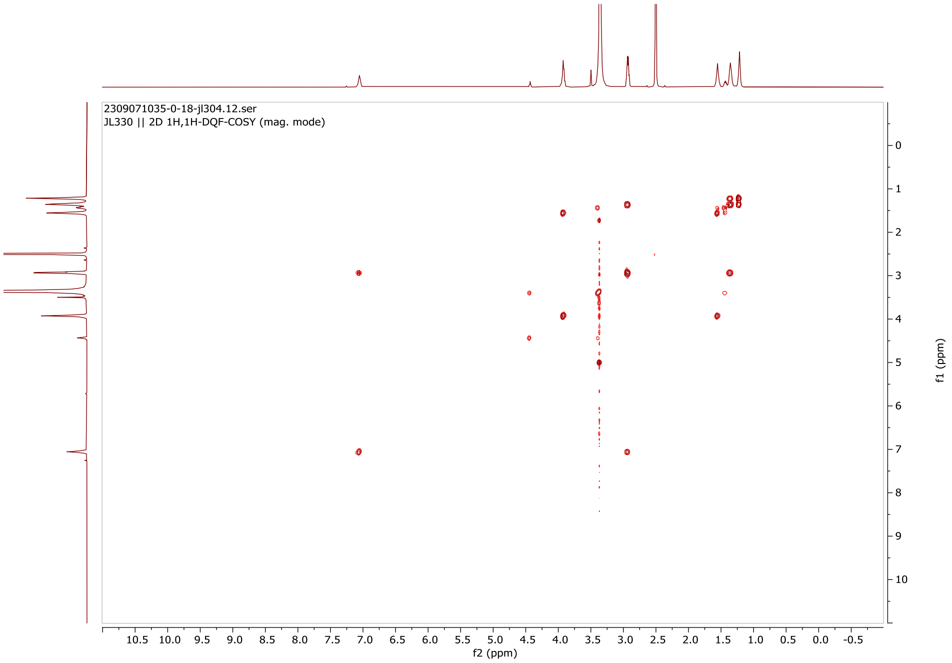

Figure S64: ^{1}H, ^{1}H COSY spectrum (DMSO-d_6, 400 MHz, 298 K) of model polyurethane **PU-2**.

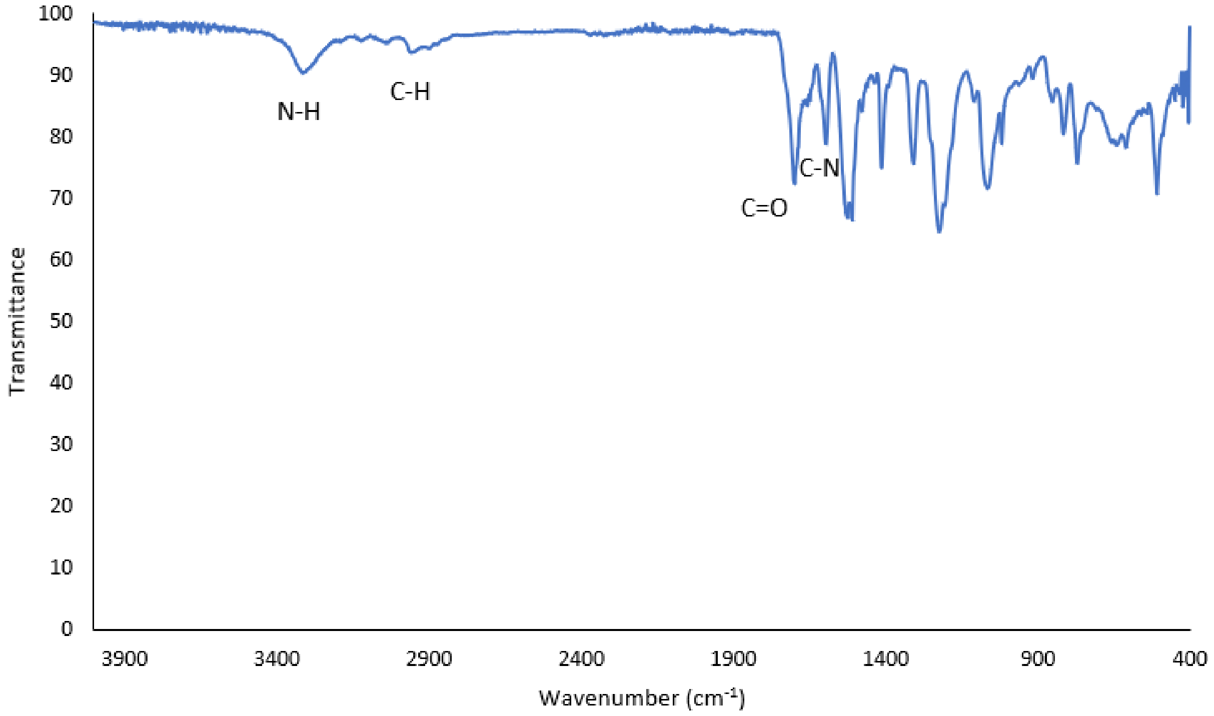

Figure S65: IR spectrum of model polyurethane **PU-1.**

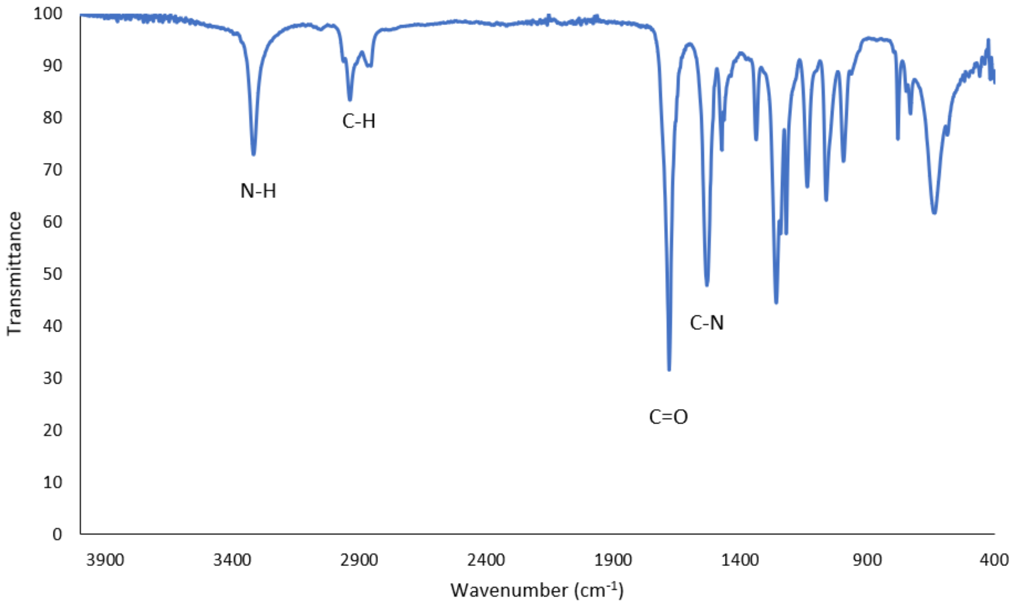

Figure S66: IR spectrum of model polyurethane **PU-2**.

8.4. NMR and GCMS data for model polyurethane hydrogenation

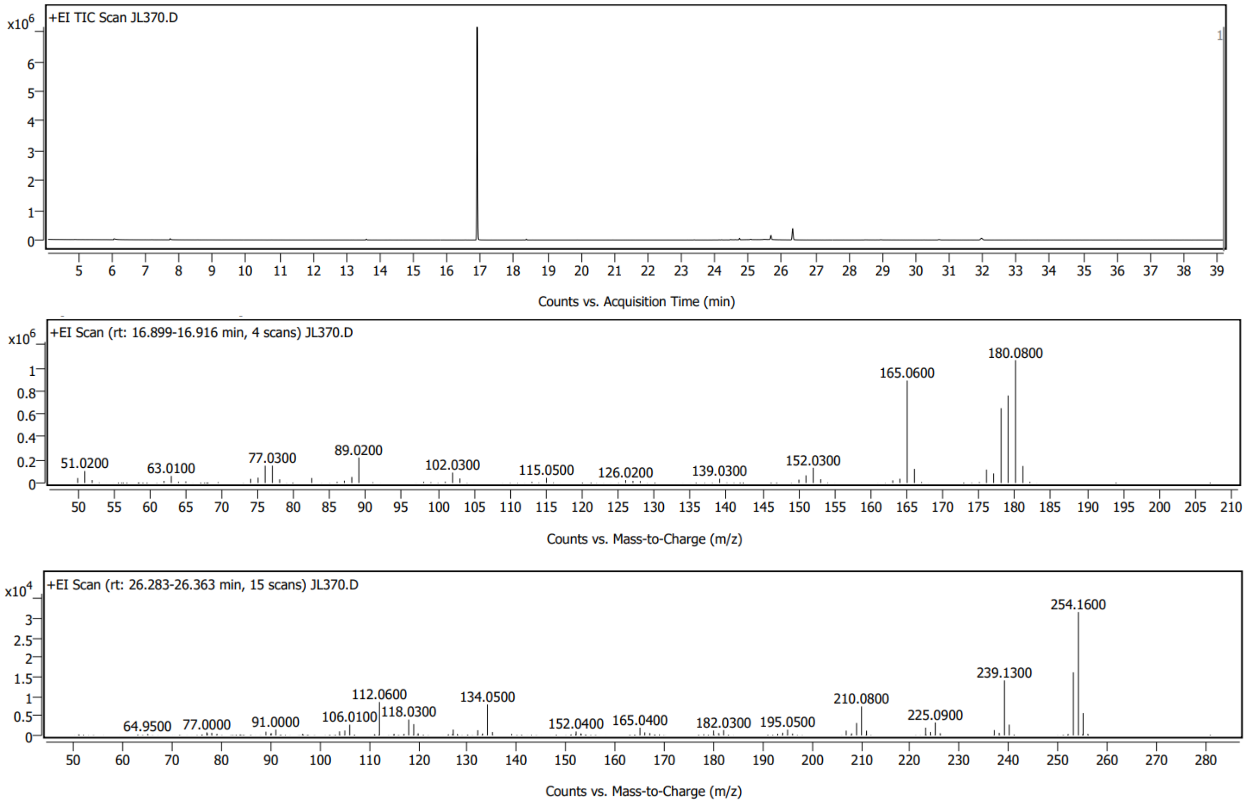

Figure S67: GCMS spectra of hydrogenation of **PU-1** in EtOH (Table S5, entry 1).

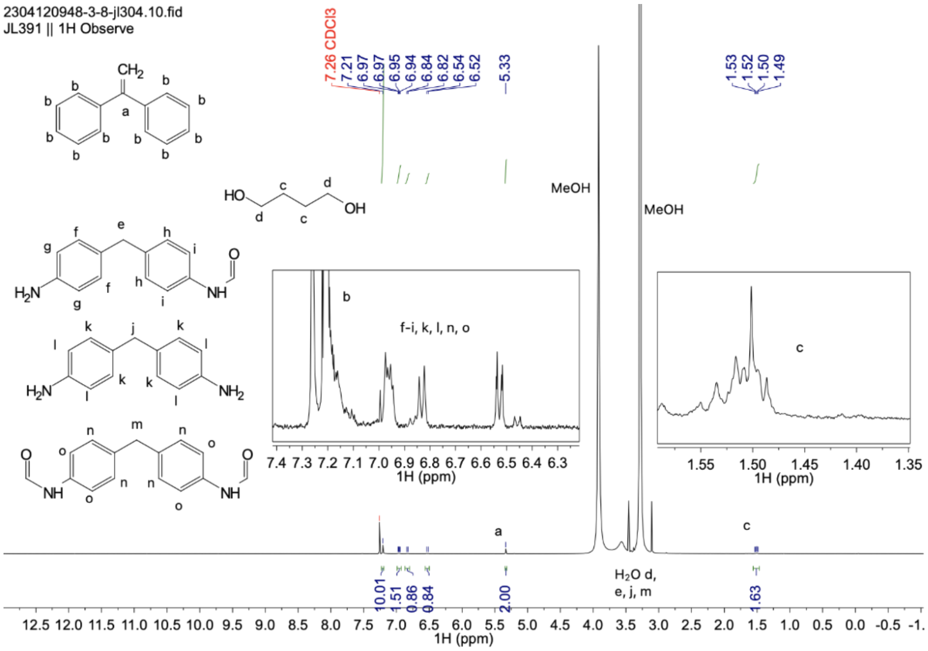

Figure S68: ^1H NMR spectrum ($CDCl_3$, 400 MHz, 298 K) of the reaction mixture of hydrogenation of **PU-1** with the internal standard 1,1-diphenylethylene (Table S5, entry 2).

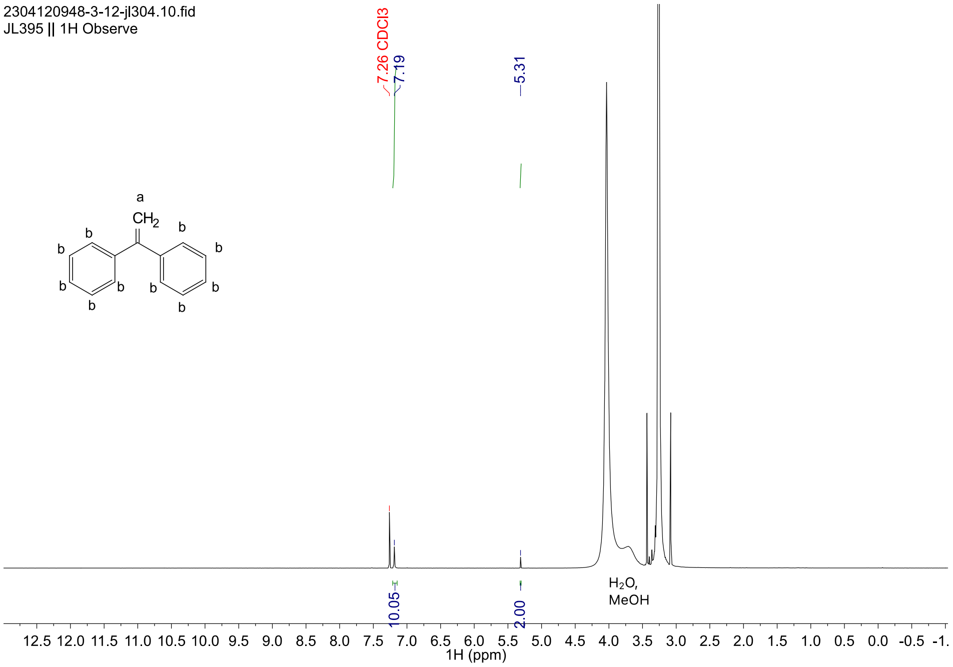

Figure S69: ^{1}H NMR spectrum ($CDCl_3$, 400 MHz, 298 K) of the reaction mixture of hydrogenation of **PU-2** with the internal standard 1,1-diphenylethylene (Table S5, entry 2).

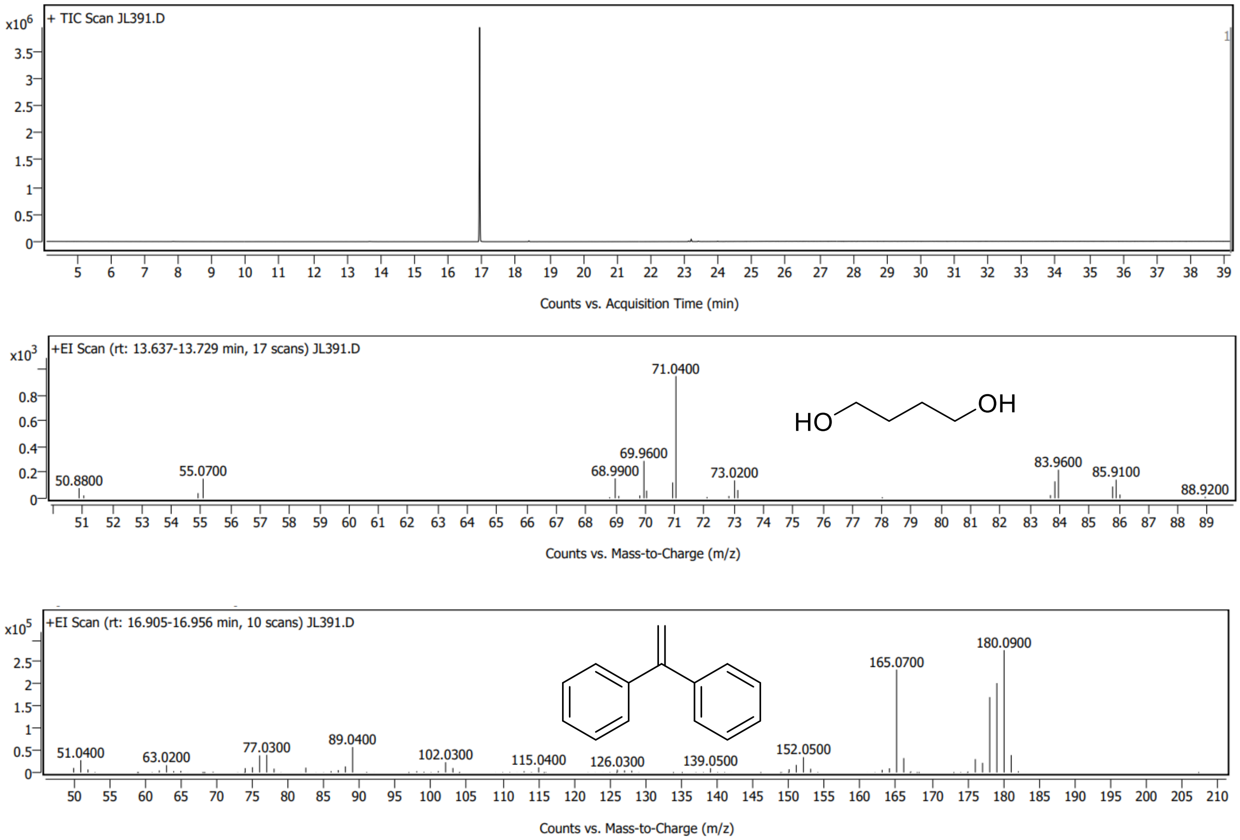

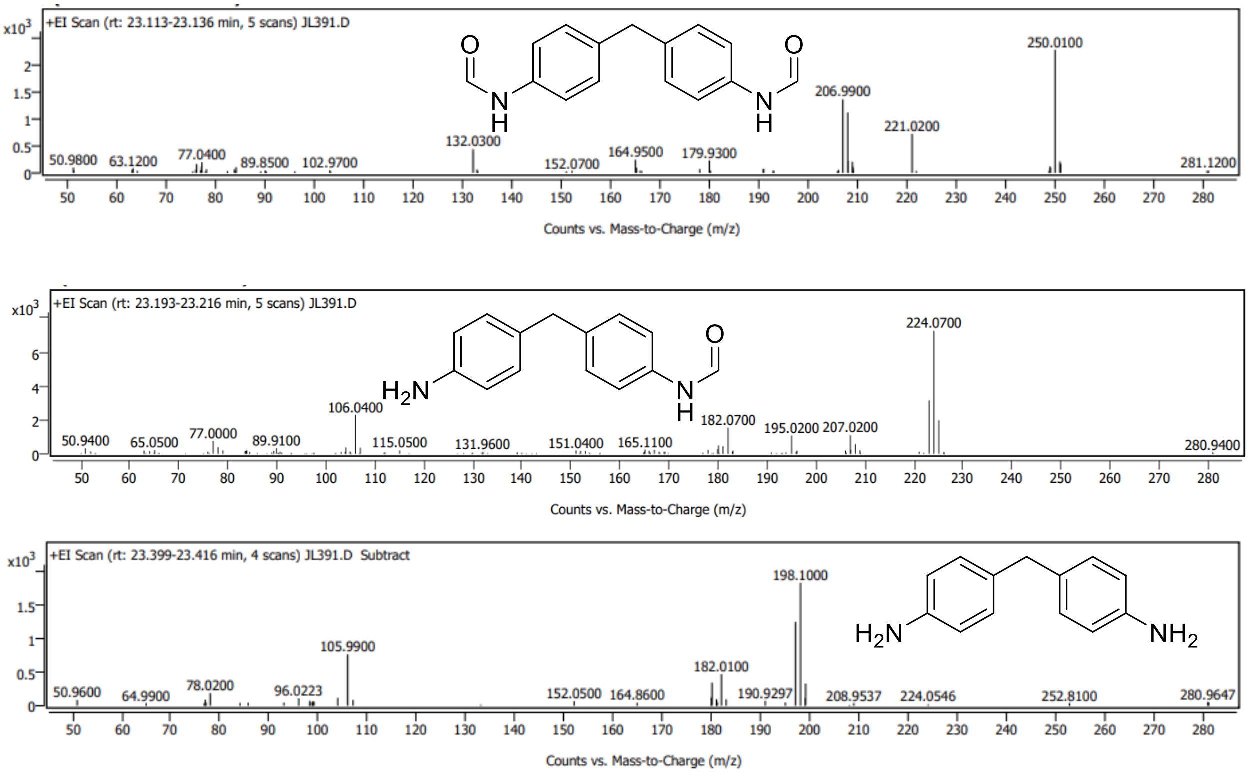

Figure S70: GCMS spectra for the reaction mixture of hydrogenation of **PU-1** with the internal standard 1,1-diphenylethylene (Table S5, entry 1).

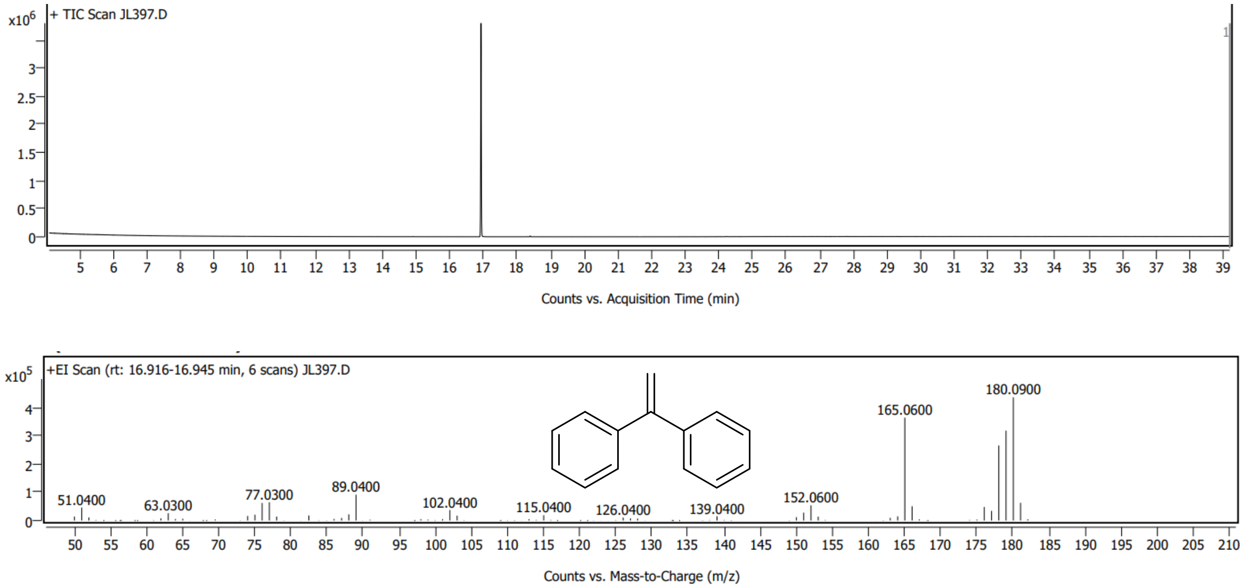

Figure S71: GCMS spectra for the reaction mixture of hydrogenation of **PU2** with the internal standard 1,1-diphenylethylene (Table S5, entry 2).

9. References

1 Widegren, M. B.; Clarke, M. L.; *Org. Lett.* **2018**, *20*, 2654– 2658.

2 Oates, C. L.; Widegren, M. B.; Clarke, M. L. *Chem. Commun.* **2020**, *56*, 8635−8638.

3 Dingwall, P.; Hembre, R. T.; Ponasik, J. A.; Tolleson, G. S.; Clarke, M. L. *Mol. Catal.* **2017**, *434*, 116-122.

4 Widegren, M. B.; Clarke, M. L.; *Catal. Sci. Technol.,* **2019**, *9*, 6047–6058.

5 US6103914A, 2000.

6 X. Chang, Q. Zhang and C. Guo, *Org. Lett.*, 2019, **21**, 4915–4918.

7 S. Shirakawa, X. Wu and K. Maruoka, *Angew. Chem. Int. Ed.*, 2013, **52**, 14200–14203.

8 Sarim, M., Alavi Nikje, M.M. & Dargahi, M.; *J. Porous. Mater.* **2023**, *30*, 227-1356.
